# Nuclear ERK1/2 signaling potentiation enhances neuroprotection and cognition via Importinα1/KPNA2

**DOI:** 10.15252/emmm.202215984

**Published:** 2023-10-04

**Authors:** Marzia Indrigo, Ilaria Morella, Daniel Orellana, Raffaele d'Isa, Alessandro Papale, Riccardo Parra, Antonia Gurgone, Daniela Lecca, Anna Cavaccini, Cezar M Tigaret, Alfredo Cagnotto, Kimberley Jones, Simon Brooks, Gian Michele Ratto, Nicholas D Allen, Mariah J Lelos, Silvia Middei, Maurizio Giustetto, Anna R Carta, Raffaella Tonini, Mario Salmona, Jeremy Hall, Kerrie Thomas, Riccardo Brambilla, Stefania Fasano

**Affiliations:** ^1^ Institute of Experimental Neurology (INSPE), IRCCS San Raffaele Scientific Institute Milano Italy; ^2^ Neuroscience and Mental Health Innovation Institute, School of Biosciences Cardiff University Cardiff UK; ^3^ NEST, Istituto Nanoscienze CNR, and Scuola Normale Superiore Pisa Italy; ^4^ Department of Neuroscience University of Torino Torino Italy; ^5^ Department of Biomedical Sciences University of Cagliari Cagliari Italy; ^6^ Neuromodulation of Cortical and Subcortical Circuits Laboratory Fondazione Istituto Italiano di Tecnologia Genova Italy; ^7^ Neuroscience and Mental Health Research Institute, School of Medicine Cardiff University Cardiff UK; ^8^ Dipartimento di Biochimica e Farmacologia Molecolare Istituto di Ricerche Farmacologiche Mario Negri‐IRCCS Milano Italy; ^9^ School of Biosciences Cardiff University Cardiff UK; ^10^ Institute of Cell Biology and Neurobiology CNR Roma Italy; ^11^ National Institute of Neuroscience Torino Italy; ^12^ Dipartimento di Biologia e Biotecnologie “Lazzaro Spallanzani” Università degli Studi di Pavia Pavia Italy

**Keywords:** cell penetrating peptide therapeutics, cognitive enhancement, ERK signaling, KPNA2, neuroprotection, Neuroscience

## Abstract

Cell signaling is central to neuronal activity and its dysregulation may lead to neurodegeneration and cognitive decline. Here, we show that selective genetic potentiation of neuronal ERK signaling prevents cell death *in vitro* and *in vivo* in the mouse brain, while attenuation of ERK signaling does the opposite. This neuroprotective effect mediated by an enhanced nuclear ERK activity can also be induced by the novel cell penetrating peptide RB5. *In vitro* administration of RB5 disrupts the preferential interaction of ERK1 MAP kinase with importinα1/KPNA2 over ERK2, facilitates ERK1/2 nuclear translocation, and enhances global ERK activity. Importantly, RB5 treatment *in vivo* promotes neuroprotection in mouse models of Huntington's (HD), Alzheimer's (AD), and Parkinson's (PD) disease, and enhances ERK signaling in a human cellular model of HD. Additionally, RB5‐mediated potentiation of ERK nuclear signaling facilitates synaptic plasticity, enhances cognition in healthy rodents, and rescues cognitive impairments in AD and HD models. The reported molecular mechanism shared across multiple neurodegenerative disorders reveals a potential new therapeutic target approach based on the modulation of KPNA2‐ERK1/2 interactions.

## Graphical abstract

Shared mechanisms governing both neuronal cell survival and neuroplasticity have not yet been explored therapeutically. This study demonstrates that modulation of ERK1 and KPNA2 interactions strengthens nuclear ERK signaling in the brain, promotes cognitive enhancement, and delays neurodegeneration.

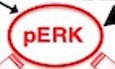

**The paper explained**

**Problem**

Neurodegenerative disorders, such as Parkinson's disease (PD), Alzheimer's disease (AD), and Huntington's disease (HS) are progressive and disabling conditions characterized by the loss of neurons in several brain areas, resulting in cognitive deficits and other behavioral symptoms. At present, known molecular mechanisms underpinning neuroprotection and cognitive enhancement, such as the ERK signaling cascade, have not yet been considered for the development of effective therapeutic strategies.

**Results**

We have demonstrated that nuclear ERK signaling potentiation via either genetic or pharmacological means can effectively prevent neuronal loss in different models of neurodegenerative disorders and rescue mild cognitive impairments. We have also found that enhanced ERK nuclear translocation and signaling are modulated by a novel mechanism based on the differential binding of ERK1 and ERK2 kinases with importinα1/KPNA2. Facilitation of nuclear ERK signaling by disrupting KPNA2 interactions also enhances memory formation in normal animals.

**Impact**

Our results provide compelling evidence that a novel mechanism of nuclear signaling based on the modulation of ERK1/KPNA2 interactions may be the basis for a novel therapeutic approach. These findings open new venues for drug development, not only for preventing neuronal loss and cognitive deficits in neurodegenerative disorders but also for cognitive enhancement in the aging population.

## Introduction

The ERK signaling pathway plays a central role in a variety of brain functions, from cell survival to synaptic plasticity and memory formation. Pharmacological and genetic evidence has shown that blockade of ERK signaling impairs long‐term memory formation and alters other forms of behavioral plasticity, while the behavioral effect of genetic manipulations of this cascade may depend on the cellular context (Fasano & Brambilla, 2011; More *et al*, 2020). On the contrary, the role of ERK signaling in neuronal survival, apoptosis, and neurodegenerative processes remains more controversial despite a clear involvement of this cascade in the mechanisms downstream to survival and plasticity‐promoting factors such as BDNF (Subramaniam & Unsicker, 2010; Numakawa *et al*, 2018).

ERK signaling results from the cytoplasmic activation of both ERK1 and ERK2 kinases, followed by their translocation into the nucleus to modulate chromatin remodeling and gene transcription. Importantly, previous work indicates that ERK1 and ERK2 play nonredundant roles. In contrast to ERK2 KO mice, which are embryonic lethal, ERK1 KO mice showed subtle phenotypes, most notably memory and synaptic plasticity improvements, that are consistent with a repressing role of ERK1 on ERK2 (Mazzucchelli *et al*, 2002; Silingardi *et al*, 2011). At the cellular level, the unique N‐terminal domain of ERK1 is responsible for the differences in the rate of nuclear shuttling, the effects on cell proliferation in culture cells, and neuronal cell signaling of the two isoforms (Vantaggiato *et al*, 2006; Marchi *et al*, 2008; Indrigo *et al*, 2010; Orellana *et al*, 2012). ERK1 and ERK2 lack a canonical nuclear localization signal (NLS), but they are phosphorylated on two Ser residues within a unique nuclear translocation signal (NTS) by Protein Kinase CK2, allowing binding to importin7/RanBP7, a member of importin β family, and subsequent penetration via nuclear pores (Maik‐Rachline *et al*, 2019; Plotnikov *et al*, 2019). However, the latter mechanism does not explain the differential rate of nuclear translocation of the two kinases. Nevertheless, altered expression of specific members of the importinα family, including importinα1/KPNA2, has been linked to alterations of ERK activity in forms of cancer (Han & Wang, 2020). In this context, the importance of nuclear translocation mechanisms to neurodegeneration has been very recently suggested, but the specific importins and proteins involved with the nuclear pore complex (NPC) remain unresolved (Hutten & Dormann, 2020; Ding & Sepehrimanesh, 2021).

In the present study, we demonstrated a novel mechanism implicating a differential interaction of ERK1 and ERK2 kinases with importinα1/KPNA2, that controls global nuclear ERK signaling and may provide a unique therapeutic target opportunity for treating neurodegenerative conditions. We designed a cell‐permeable peptide able to interfere with ERK1, ERK2, and KPNA2 interactions, thus selectively potentiating nuclear ERK activation, attenuating neuronal cell loss in models of neurodegeneration, improving cognition in healthy rodents, and rescuing cognitive deficits in Alzheimer's (AD) and Huntington's (HD) disease mouse models. Hence, our results suggest that enhancing ERK signaling is an attractive target for neurodegenerative diseases and cognitive enhancement.

## Results

### Altering ERK1/ERK2 ratio affects neuronal cell survival

**Figure 1:**
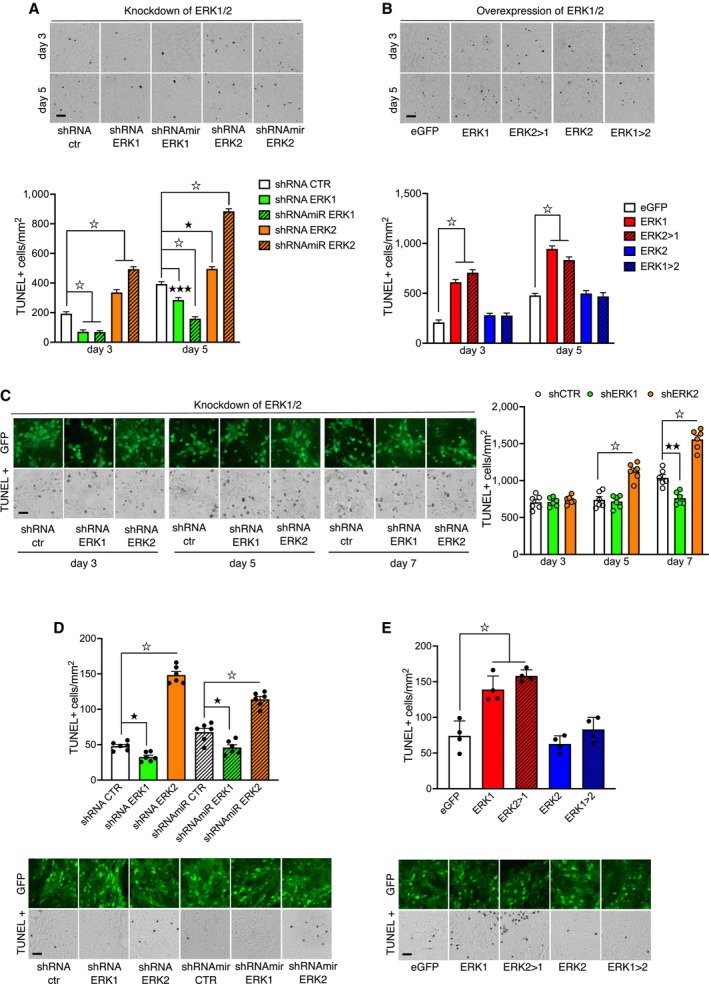
ERK1/ERK2 MAP Kinase ratio controls cell survival (Top panel) Representative images of the TUNEL assay in MEFs after knockdown of ERK1 or ERK2 (bottom panel) shRNA ERK1 and shRNAmir‐ERK1 decreased apoptosis, whereas shRNA or shRNAmir for ERK2 increased cell death at days 3 and 5 (*n* = 14–25 slides per group).(Top panel) Representative images of the TUNEL assay in MEFs overexpressing ERK1, ERK2, or their chimeric constructs (bottom panel). Overexpression of ERK1 or ERK2 > 1 increased apoptosis at days 3 and 5. However, overexpression of ERK2 or ERK1 > 2 did not alter cell survival compared to controls (*n* = 9–12 slides per group).(Left panel) Representative images of the TUNEL assay in cortical embryonic cultures after knockdown of ERK1 or ERK2 (right panel). The shRNA ERK2 group increased cell death at days 5 and 7. In contrast, shRNA ERK1 reduced apoptosis only at day 7 (*n* = 6 slides per group).TUNEL assay performed on striatal slices of adult mice after knockdown of ERK1 or ERK2. ERK2 knockdown enhanced cell death, while ERK1 knockdown slightly reduced apoptosis (*n* = 4 mice per group).TUNEL assay performed on striatal slices of adult mice overexpressing ERK1, ERK2, or their chimeric constructs. Striatal overexpression of ERK1 or ERK2 > 1 enhanced apoptosis. In contrast, mice overexpressing ERK2 or ERK1 > 2 showed levels of apoptosis comparable to eGFP controls (*n* = 6 mice per group). Data information: Results show mean ± s.e.m. ^☆^ *P* < 0.0001, ^★★★^ *P* < 0.001, ^★★^ *P* < 0.01, ^★^ *P* < 0.05. A full statistical analysis is reported in Appendix Table S1. Scale bars: 50 μm. Source data are available online for this figure.

The exact role of ERK1/2 in cell survival remains unclear; both pro‐survival and pro‐apoptotic mechanisms have been suggested. In order to clarify this matter, we selectively knocked down ERK1 or ERK2 in proliferating mouse embryo fibroblasts (MEFs) using previously *in vitro* validated lentiviral vectors (LV) expressing either shRNA or shRNAmir for both ERK1 and ERK2 (Indrigo *et al*, 2010). ERK1 knockdown promoted cell survival, while ERK2 knockdown increased apoptosis (Fig 1A). Conversely, cell death was induced by the overexpression of either ERK1 or a chimeric protein in which the N‐terminus of ERK1 was fused to ERK2 (termed ERK2 > 1; Orellana *et al*, 2012). Overexpression of ERK2, or ERK1 with its N‐terminus removed (termed ERK1 > 2), had little effect on cell survival (Fig 1B). In neuronal embryonic cultures, ERK1 knockdown similarly promoted survival, while ERK2 knockdown increased apoptosis (Fig 1C), confirming that this mechanism is also acting in postmitotic neurons (Papale *et al*, 2009; Orellana *et al*, 2012). Our results are consistent with the opposing roles of the two proteins in cell survival and with the critical role of the N‐terminus of ERK1, thought to be responsible for the functional differences between the two isoforms (Vantaggiato *et al*, 2006; Marchi *et al*, 2008).

**Figure EV1:**
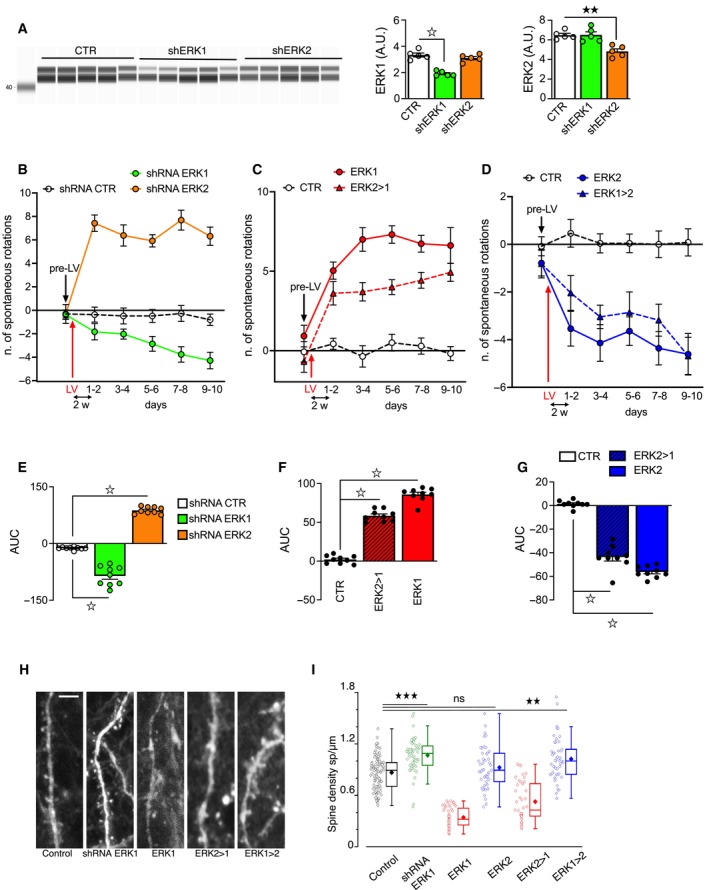
ERK1/ERK2 MAP kinase ratio controls rotational behavior and striatal spine density AWestern blot analysis of striatal extracts obtained from adult mice after knockdown of ERK1 or ERK2. shRNA ERK1 and shRNA ERK2 specifically reduced ERK1 and ERK2 protein levels, respectively (*n* = 5 mice per group).B–GSpontaneous rotational behavior measured after knockdown or overexpression of ERK1 and ERK2, or their chimeric constructs, 2 weeks (2w) post‐LV injection (*n* = 13–29 mice per group). (B) Control mice (bilateral striatal injection of ctr shRNA) showed an equal number of 180° rotations to either side. Unilateral expression of shRNA ERK1 (combined with contralateral ctr shRNA injection) induced net contralateral rotations (negative values), whereas shRNA ERK2 induced increased ipsilateral rotations (positive values). (E) Area under the curve (AUC) analysis over 10 days. (C) Mice unilaterally overexpressing ERK1 or ERK2 > 1 showed increased ipsilateral rotations (positive values). (F) AUC analysis over 10 days. (D) Mice overexpressing ERK2 or ERK1 > 2 showed net contralateral rotations (negative values) compared to control mice (G) AUC analysis over 10 days.HRepresentative images of dendritic spines on striatal neurons after knockdown or overexpression of ERK1, ERK2, or their chimeric constructs.INeurons of mice overexpressing ERK1 or ERK2 > 1 showed decreased spine density, while mice overexpressing ERK1 > 2 and mice with ERK1 downregulated showed increased spine density. Data were obtained from 3 to 9 mice per group. The mean for each group is represented by the solid diamonds, the median by the horizontal bars in the box plot, the box upper and lower edges are the 75% limits and the whiskers are the 90% limits. Data information: Results show mean ± s.e.m. ^☆^ *P* < 0.0001, ^★★★^ *P* < 0.001, ^★★^ *P* < 0.01, ^★^ *P* < 0.05. A full statistical analysis is reported in Appendix Table S1. Scale bar: 5 μm. Source data are available online for this figure.

To test whether ERK manipulation causes similar effects *in vivo*, we bilaterally injected the LVs into the dorsal striatum of adult mice and assessed the number of apoptotic cells 2 weeks later. Consistent with the *in vitro* data, *in vivo* dorsal striatal injection of LV expressing shRNA for ERK1 did cause an overall reduction of p44 ERK1 of 42.6%, while the corresponding LV expressing shRNA for ERK2 selectively reduced p42 ERK2 of 25% (Fig EV1A), in accordance with previous reports (Indrigo *et al*, 2010; Bido *et al*, 2015). Confirming the results of *in vitro* experiments, we found that ERK2 knockdown promoted cell death while ERK1 ablation slightly reduced basal levels of apoptosis (Fig 1D). Moreover, apoptotic cells were significantly increased in the dorsal striatum of mice overexpressing ERK1 or ERK2 > 1. In contrast, overexpression of ERK2 or ERK1 > 2 had no effect (Fig 1E), as in neuronal cultures.

**Figure 2:**
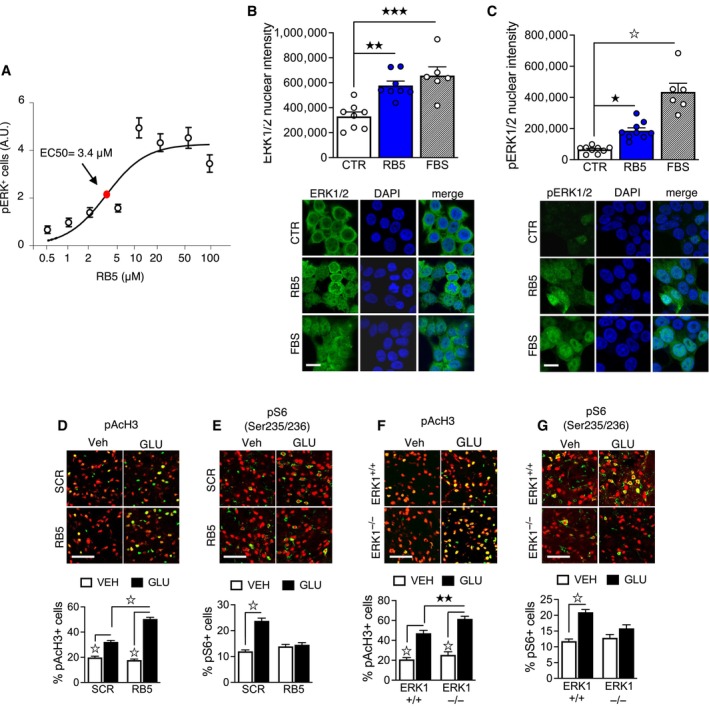
RB5 specifically activates nuclear ERK signaling in both HEK293 cells and acute striatal slices ALogarithmic dose‐response curve of RB5 activation of ERK (expressed as arbitrary units, AU) in fresh striatal slices from adult mice treated with different doses of RB5. RB5 enhanced pERK with an EC50 of 3.4 μM. Data were obtained from *n* = 8–12 slices per group.B, CHEK293 cells were treated with 50 μM RB5, FBS 20% (positive control) for 15 min or untreated (CTR, negative control). Cells were processed for ERK1/2 (B) and phospho‐ERK1/2 immunofluorescence (C). Data have been obtained from two independent experiments (*n* = 6–9 slides per group) (B, top panel). Both RB5 and FBS induced ERK1/2 translocation into the nucleus. (B, bottom panel) Representative images of ERK1/2 immunofluorescence. (C, top panel) Both RB5 and FBS induced phospho‐ERK1/2 translocation into the nucleus. (C, bottom panel) Representative images of phospho‐ERK1/2 immunofluorescence. Scale bar: 20 μm.D, E(top panels) Representative merged images showing histone AcH3 phosphorylation (D) and ribosomal S6 protein phosphorylation (E) in *ex vivo* acute slices pretreated for 1 h with 50 μM Scramble (SCR) or RB5 peptide and stimulated for 10 min with glutamate (GLU, 100 μM) or Vehicle (Veh). Red: NeuN. Green: pAcH3, pS6. Scale bar: 50 μm. (D, bottom panel) RB5 enhanced glutamate‐induced AcH3 phosphorylation but (E, bottom panel) prevented glutamate‐mediated S6 activation. Data were obtained from two independent experiments (*n* = 9–19 slices per group).F, G(top panels) Representative merged images showing pAcH3 (F), and pS6 (G) on slices from ERK1^−/−^ mice pretreated *ex‐vivo* for 1 h with SCR or RB5 peptide (50 μM) and stimulated with glutamate or vehicle for 10 min. Red: NeuN. Green: pAcH3, pS6. Scale bar: 50 μm. (F, bottom panel) In response to glutamate, pAcH3 is enhanced in ERK1^−/−^ slices (two independent experiments, *n* = 11 slices) (G, bottom panel). Glutamate‐mediated pS6 enhancement is prevented in ERK1^−/−^ mice (two independent experiments, *n* = 8–15 slices). Data information: Results show mean ± s.e.m. ^☆^ *P* < 0.0001, ^★★★^ *P* < 0.001, ^★★^ *P* < 0.01, ^★^ *P* < 0.05. A full statistical analysis is reported in Appendix Table S1. Source data are available online for this figure.

The striatum is a key structure within the basal ganglia circuits involved in motor control; its dysfunction is associated with neurodegenerative disorders, notably Parkinson's Disease (PD) and HD. To gain insights into the long‐term effects of ERK1/2 manipulations at the circuit level, we examined the behavior of mice unilaterally injected with the above validated LVs into the dorsal striatum, considering the major role of ERK signaling in the control of asymmetric behavior (Cai *et al*, 2000). Unilateral reduction of striatal ERK1 induced contralateral spontaneous rotations, while reduction of ERK2 induced ipsilateral spontaneous rotations (Fig EV1B–E). Accordingly, unilateral overexpression of ERK1 and ERK2 > 1 caused ipsilateral rotations (Fig EV1C–F) while unilateral overexpression of ERK2 and ERK1 > 2 induced contralateral rotations (Fig EV1D–G). These results clearly indicate that ERK1 has a role in depressing motor behavior, while ERK2 has the opposite role of promoting motor behavior.

Enhanced ERK signaling resulting in contralateral turning behavior may be associated with changes in structural plasticity and spine formation, two processes highly dependent on ERK activity (Cerovic *et al*, 2013). Indeed, we found that altering the ERK2/ERK1 ratio resulted in spine density changes on the spiny projection neurons (SPNs). While a reduction of ERK1 increased spine formation, ERK1 (or ERK2 > 1) overexpression decreased it. In contrast, overexpression of ERK2 (or ERK1 > 2) did not significantly modify spine density (Fig EV1H and I). Thus, a reduced ERK2/ERK1 ratio is detrimental for both cell survival and spine stability. Specifically, an increased ERK2/ERK1 ratio increased spine density, likely providing a structural and neurophysiological basis for the behavioral asymmetry towards increased motor behaviors. The decreased ERK2/ERK1 ratio may also increase striatal apoptosis, raising the hypothesis that potentiation of ERK2‐mediated gene regulation could have therapeutic implications for disorders of the basal ganglia.

### RB5, a cell penetrating peptide, enhances nuclear ERK signaling by mimicking ERK1 deficiency

**Figure EV2:**
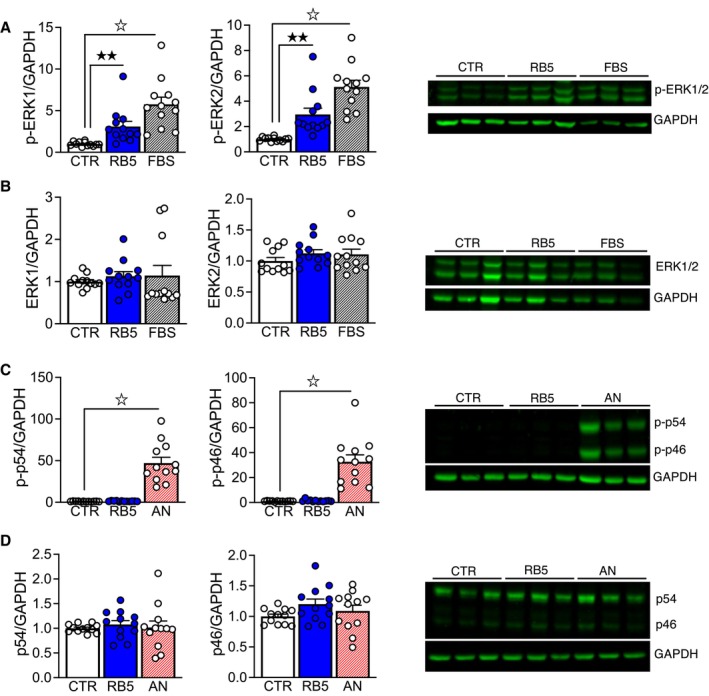
RB5 specifically activates ERK1 and ERK2 in HEK293 cells A, BHEK293 cells were treated with RB5 50 μM, or FBS 20% (positive control) for 15 min, or untreated (CTR, negative control). Data were obtained from two independent experiments (*n* = 12 independent samples per group). RB5 treatment significantly increased ERK1 and ERK2 phosphorylation (A, left panel) with no effect on total ERK1 and ERK2 levels (B, left panel). (A, B, right panels) Representative Western blots of pERK1/2 and total ERK1/2.C, DHEK293 cells were treated with RB5 50 μM or anisomycin 5 μM (AN) as a positive control for 30 min or untreated cells (CTR, negative control). Data were obtained from two independent experiments (*n* = 12 independent samples per group). RB5 treatment did not affect either phospho‐JNK p54 and p46 (C, left panel) or total JNK p54 and p46 levels (D, left panel). (C, D, right panels) Representative Western blots of phospho‐JNKs and total JNKs. Data information: Results show mean ± s.e.m. ^☆^ *P* < 0.0001, ^★★^ *P* < 0.01. A full statistical analysis is reported in Appendix Table S1. Source data are available online for this figure.

The N‐terminus of ERK1, which affects nuclear‐cytoplasmic shuttling (Marchi *et al*, 2008), is crucial for the observed phenotypes. To interfere with ERK1 function, we designed a cell‐penetrating peptide named RB5, corresponding to amino acids 7–38 of Human MAPK3 (ERK1) sequence, with the aim of providing a pharmacological tool to displace p44 ERK1 from binding partners, which may be responsible for the delayed nuclear‐cytoplasmic shuttling. In serum‐starved HEK 293 cells, the application of 50 μM RB5 for 15 min sustained a strong p44 ERK1 and p42 ERK2 phosphorylation, comparable to 20% fetal bovine serum (FBS), without affecting p44/p42 protein levels (Fig EV2A and B). Importantly, this effect was specific to ERK signaling since the closely related p54 and p46 JNK kinases were not stimulated by RB5 treatment (Fig EV2C and D). Accordingly, in acute striatal slices pretreated with increasing concentrations of RB5, the peptide selectively promoted ERK phosphorylation (Fig 2A), with an estimated EC50 of 3.4 μM.

To determine whether RB5 facilitates ERK1/2 nuclear translocation, HEK293 cells were stimulated with either 50 μM RB5 or 20% FBS and stained with p44/p42 and phospho‐p44/p42‐specific antibodies. RB5 rapidly enhanced nuclear signals of both total and phosphorylated ERK1/2 (Fig 2B and C). To further confirm the enhancing effect of RB5 on nuclear ERK1/2, acute striatal slices obtained from adult mice were pretreated with RB5 and then challenged for 10 min with 100 μM glutamate. RB5 potentiated glutamate‐mediated phosphorylation of a nuclear ERK1/2 target (Acetyl‐Histone H3) but blunted the phosphorylation of the cytoplasmic ERK1/2 target ribosomal protein S6 (Ser 235/236; Fig 2D and E). Significantly, the enhancing and inhibitory effects of RB5 on pAcH3‐and S6‐glutamate‐induced phosphorylation, respectively, were similar to those induced by ERK1 ablation in ex‐vivo slices obtained from ERK1 KO mice (Fig 2F and G). Altogether, these results strongly suggest that RB5 targets the ERK1 protein to modulate global ERK1/2 signaling.

**Figure 3:**
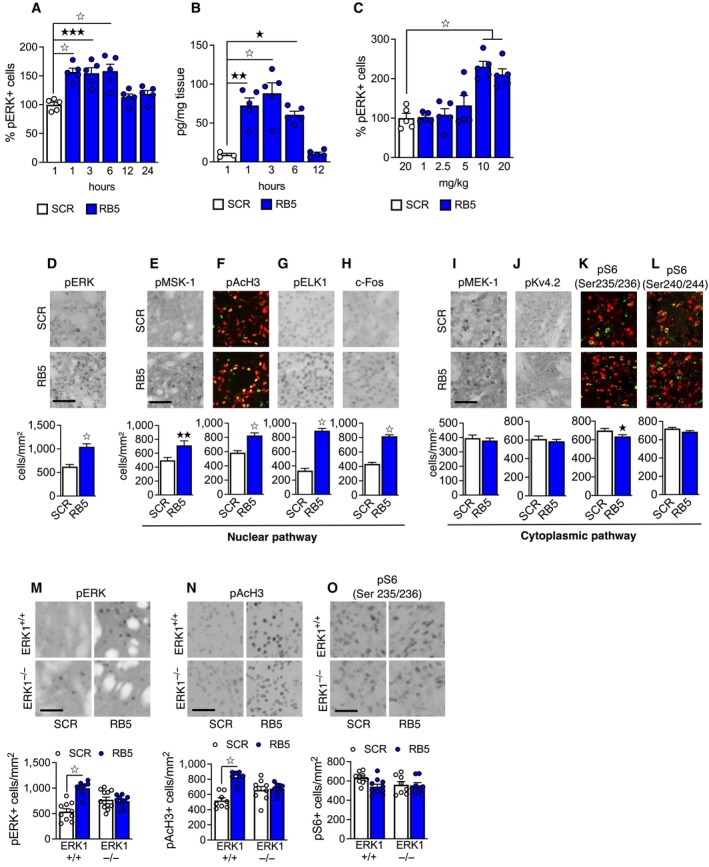
RB5 selectively enhances striatal nuclear ERK signaling *in vivo* Ap‐ERK‐positive cells in dorsal striatum induced by a single dose of RB5 (20 mg/kg, i.p.) at different time points. Scramble (SCR)‐injected mice (20 mg/kg, i.p.) were perfused after 1 h. RB5‐mediated enhancement of ERK phosphorylation lasted up to 6 h post injection (*n* = 5 mice per group).BRB5 brain levels measured with mass spectrometry at different time points after a single administration (20 mg/kg i.p.). High levels of RB5 were detected up to 6 h (*n* = 3–5 mice per group).Cp‐ERK‐positive cells in the dorsal striatum induced by increasing doses of RB5 (1.5, 2.5, 5, 10, and 20 mg/kg, i.p.) 1 h after administration. SCR peptide was injected at 20 mg/kg, i.p. All mice were perfused after 1 h. RB5 caused an increase in ERK phosphorylation at both 10 and 20 mg/kg.D–L(top panels) Representative images showing p‐ERK, nuclear ERK‐dependent markers, and cytoplasmic markers in the striata of mice 1 h after a single injection with RB5 or SCR peptide (20 mg/kg, i.p.). Red: NeuN. Green: pAcH3, pS6 (Ser235/236), pS6 (Ser240/244). (bottom panels) RB5 increased ERK phosphorylation (D) and promoted a selective activation of nuclear ERK‐dependent markers pMSK‐1 (E), pAcH3 (F), pELK (G), and c‐Fos (H). Differently, RB5 did not affect the phosphorylation of cytoplasmic proteins MEK‐1 (I) or Kv4.2 (J). RB5 slightly reduced ERK‐dependent phosphorylation of S6 (Ser235/236) (K), but had no effect on mTOR/TORC1‐dependent phosphorylation of S6 (Ser240/244) (L). Data were obtained from *n* = 14–16 mice per group.M–O(top panels). Representative images of phosphorylation of ERK, AcH3, and S6 in the striata of ERK1^−/−^ mice after a single RB5 injection (20 mg/kg, i.p.) (M, bottom panel). RB5 increased ERK phosphorylation in WT but not in ERK1^−/−^ mice. (N, bottom panel) RB5 increased pAcH3 in WT mice. (O, bottom panel) RB5 treatment did not affect S6 (Ser235/236) phosphorylation in both WT and ERK1^−/−^ mice (*n* = 8–10 mice per group). Data information: Scale bars: 50 μm. Results show mean ± s.e.m. ^☆^ *P* < 0.0001, ^★★★^ *P* < 0.001, ^★★^ *P* < 0.01, ^★^ *P* < 0.05. A full statistical analysis is reported in Appendix Table S1. Source data are available online for this figure.

We characterized the basic pharmacokinetics and pharmacodynamics of RB5, an essential step before using it *in vivo*. ERK phosphorylation in the striatum was sustained up to 6 h after acute systemic administration of RB5, suggesting its half‐life is 6–12 h (Fig 3A). Mass spectrometry confirmed brain bioavailability. RB5 was detectable in up to 6 h postinjection (Fig 3B). In a dose response study, both 10 and 20 mg/kg i.p. resulted in significant striatal ERK activation *in vivo* 1 h later (Fig 3C). At this time point, the highest dose of RB5 greatly enhanced striatal ERK phosphorylation (Fig 3D) and selectively activated nuclear ERK‐dependent signaling (pMSK, pAcH3, pELK, c‐Fos; Fig 3E–H). RB5 did not influence the phosphorylation of two other cytoplasmic ERK targets: MEK‐1 (Fig 3I) and voltage‐gated K^+^ (Kv4.2) channel (Fig 3J). Moreover, ERK‐dependent phosphorylation of S6 (Ser235/236) was slightly reduced (Fig 3K), as *in vitro*. Importantly, there was no effect on TORC1‐dependent phosphorylation of S6 (Ser240/244; Fig 3L) highlighting the selectivity of RB5 on nuclear ERK signaling.

The effects of RB5 resemble in part the behavior of ERK1‐deficient cells, in which the stimulus‐dependent activation of ERK2 is enhanced (Mazzucchelli *et al*, 2002; see also Fig 2F and G). To confirm *in vivo* the requirement of p44 ERK1 for RB5 activity, we administered it to ERK1 KO mice and found that a single administration of RB5 enhanced pERK and pAcH3 in WT but not in ERK1 KO mice (Fig 3M and N). Phosphorylation of cytoplasmic S6 (Ser235/236) was comparable in both genotypes, confirming that RB5 preferentially activates nuclear signaling (Fig 3O). Overall, RB5 can be considered a pharmacological model of ERK1 deficiency by mimicking the increase of the ERK2/ERK1 ratio and by selectively stimulating processes closely associated with ERK‐mediated gene expression and epigenetic remodeling in the brain.

### Importinα1/KPNA2 is the target of RB5 action and differentially binds ERK1 and ERK2

**Figure 4:**
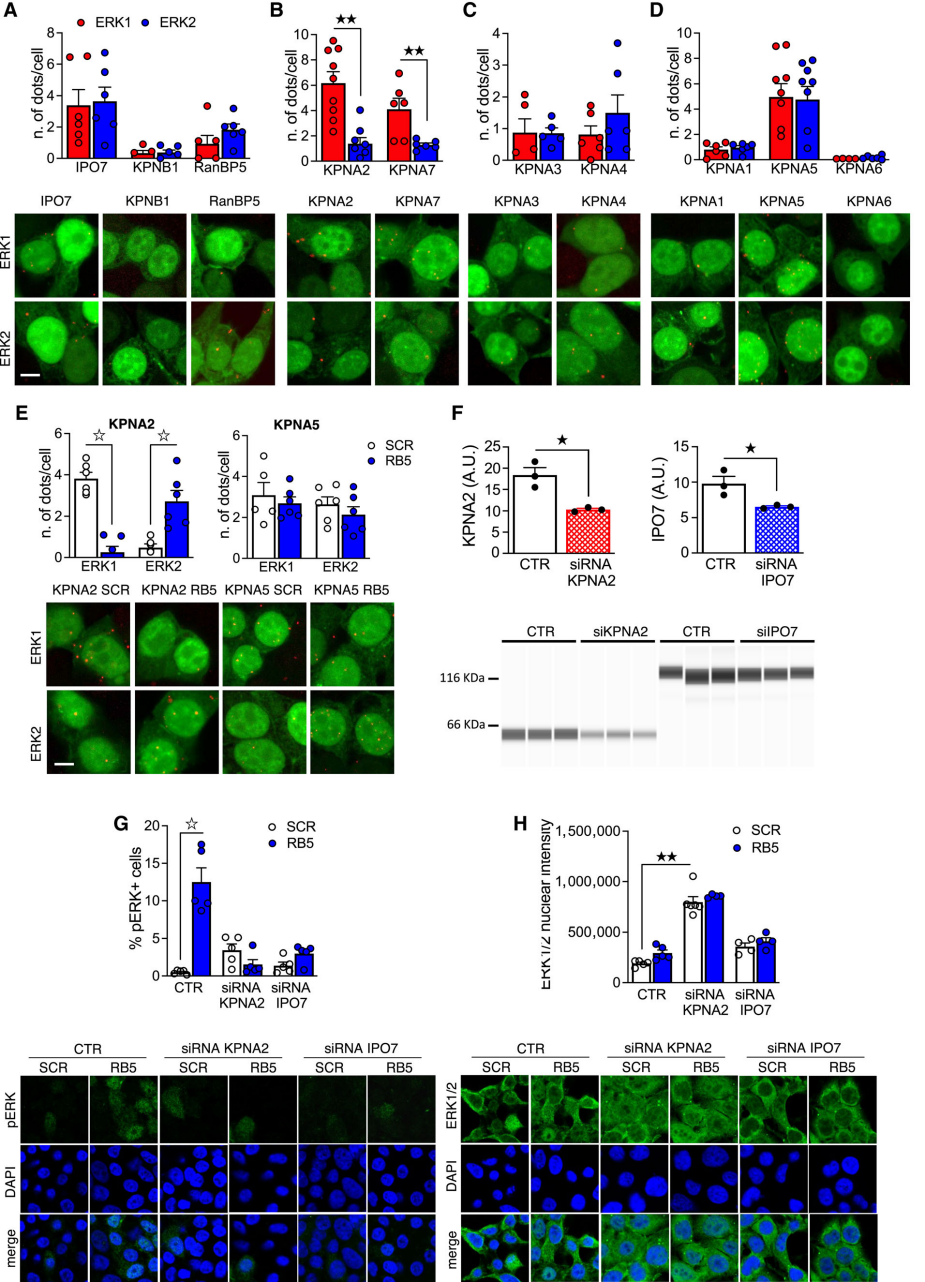
KPNA2 and KPNA7 preferentially interact with ERK1 A–EHEK293 cells were transfected with tagged importins together with GFP, ERK1‐GFP, or ERK2‐GFP and processed for PLA. Data were obtained from 2 to 3 independent experiments (*n* = 5–9 slides per condition). Data are shown as number of dots/cells in ERK1‐or ERK2 transfected cells—number of dots/cells in GFP transfected cells. In all experiments, few aspecific interactions occurred between the importins and the GFP alone, but the number of interactions in ERK1 and ERK2‐transfected cells was significantly higher. (A–E) (bottom panels) Representative images of PLA (green: GFP, red: PLA interactions). (A, top panel) IPO7, KPNB1, and RanBP5 did not preferentially interact with ERK1 or ERK2. (B, top panel) KPNA2 and KPNA7 preferentially interacted with ERK1. (C, top panel) KPNA3 and KPNA4 did not show a preferential interaction with ERK isoforms. (D, top panel) KPNA1, KPNA5, and KPNA6 did not show a preferential interaction with ERK isoforms. (E) RB5 treatment (50 μM for 1 h) specifically prevented the interaction of KPNA2 with ERK1, while promoting the binding with ERK2. The interaction of KPNA5 with ERK1 and ERK2 was not affected by RB5 treatment.FHEK293 cells were treated with siRNA KPNA2, siRNA IPO7 or not treated (CTR). Western blot analysis revealed an effective downregulation of KPNA2 and IPO7 induced by the respective siRNAs (*n* = 3 independent samples per group).G–HImmunofluorescence analysis of HEK293 pretreated with siRNA KPNA2 and siRNA IPO7 (200 nM for 24 h) or not treated (CTR) and subsequently stimulated with RB5 (50 μM for 15 min). (G, top panel) RB5 failed to induce ERK1/2 activation upon KPNA2 or IPO7 downregulation. (H, top panel) Downregulation of KPNA2 enhanced ERK1/2 nuclear translocation. However, RB5 treatment did not further enhance ERK1/2 nuclear translocation (*n* = 4–6 slides per group). Data information: Scale bars: 10 μm. Results show mean ± s.e.m. ^☆^ *P* < 0.0001, ^★★^ *P* < 0.01, ^★^ *P* < 0.05. A full statistical analysis is reported in Appendix Table S1. Source data are available online for this figure.

**Figure EV3:**
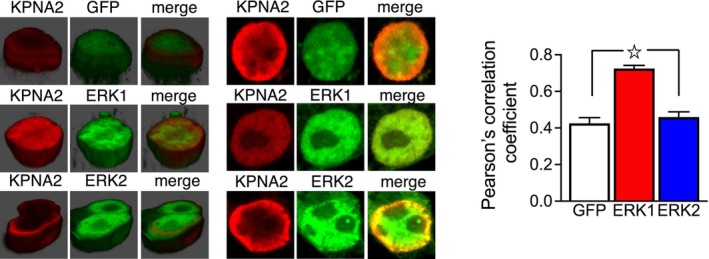
KPNA2 preferentially colocalizes with ERK1 HEK293 cells were transfected with GFP, ERK1‐GFP, or ERK2‐GFP together with KPNA2‐T7 (left panel). Representative tridimensional and bidimensional images (right panel). Pearson's correlation coefficient was increased in ERK1‐transfected cells. Data were obtained from *n* = 83–90 cells per group. Results show a mean ± s.e.m. ^☆^ *P* < 0.0001. A full statistical analysis is reported in Appendix Table S1.Source data are available online for this figure.

To identify the direct molecular target of the RB5 sequence, we focused our attention on the nuclear‐cytoplasmic transport machinery. Previous work indicated that importin 7 (IPO7/RanBP7), a member of the Importin β family, mediates the nuclear localization of ERK1 and ERK2 (despite lacking a canonical nuclear localization signal) in mammalian cells and in *Drosophila* (Plotnikov *et al*, 2015; Li *et al*, 2016). However, it is not known whether ERK1 and ERK2 differentially bind to IPO7/RanBP7 and whether the RB5 sequence modulates ERK‐IPO7/RanBP7 interactions. To address these gaps, we took advantage of the proximity ligation assay (PLA), a sensitive quantitative method to assess protein–protein interactions (Hegazy *et al*, 2020). GFP‐tagged ERK1 and ERK2 kinases were co‐transfected in HEK293 cells with epitope‐tagged IPO7/RanBP7. As additional controls we also investigated the interaction of ERK1 or ERK2 with two other major Importin β family members, Ipo5/RanBP5 and Ipo1/KPNB1. Both ERK1 and ERK2 strongly bind to IPO7, while the binding to the other two β Importins was less pronounced (Fig 4A). In any case, we did not observe any preferential binding of β Importins to p44 ERK1. The Importin α family consists of seven members and through specific interactions with Importin β, mediates nuclear‐cytoplasmic transport via the nuclear pore complex (NPC; Oka & Yoneda, 2018). We systematically assessed ERK1 and ERK2 binding to all Importin α members using PLA. Two more closely related KPNA2 and KPNA7 Importin α (α1 subclass) showed significantly higher binding of ERK1 than ERK2 (Fig 4B). In contrast, KPNA3, KPNA4, KPNA1, KPNA5, and KPNA6 did not show this selective interaction (Fig 4C and D). Since KPNA2 is the best characterized member of the KPNA2/7 subgroup and is highly expressed in the adult brain, we characterized its interactions with ERK1 and ERK2 further. To test the hypothesis that the N‐terminal domain/RB5 target sequence of ERK1 may be the structural element responsible for its differential interaction with KPNA2, we performed an ERK1‐KPNA2 competition assay by pretreating HEK293 cells with RB5 50 μM for 1 h before PLA (Fig 4E). RB5 treatment caused a 10‐fold decrease in ERK1 binding to KPNA2, but unexpectedly, RB5 concomitantly induced a 6‐fold increase in ERK2 binding (Fig 4E, left panel). As a control, ERK1 or ERK2 binding to KPNA5 was unaffected by RB5 treatment (Fig 4E, right panel). This evidence suggests a complex interplay between KPNA2, ERK1, and ERK2 but importantly supports a central role of RB5 sequence in modulating their interactions. To further gain insights on the functional relevance of ERK1‐KPNA2 binding, we used 2D and 3D (STED) confocal microscopy and found that KPNA2 preferentially colocalizes with ERK1 under basal conditions (Fig EV3). Moreover, the overexpression of ERK1, but not ERK2, seemed to alter KPNA2 cellular distribution from a perinuclear to a nuclear localization. To confirm the involvement of KPNA2 in the cellular localization and activation of ERK1 and ERK2, we also took a complementary approach by knocking down endogenous KPNA2 in HEK293 cells using small interfering RNA technology (siRNA). IPO7 was also downregulated, to provide a positive control. Two hundred nanometers of siRNA‐KPNA2 and siRNA‐IPO7 efficiently reduced the corresponding protein levels by 37.9 and 33.2%, respectively (Fig 4F). Quite surprisingly, we observed different responses induced by siRNA‐KPNA2 and siRNA‐IPO7 in terms of total ERK1/2 nuclear phosphorylation and total nuclear protein accumulation when starved HEK293 cells were stimulated with RB5. Both KPNA2 and IPO7 knockdowns prevented ERK1/2 nuclear phosphorylation, indicating that both importins are essential for nuclear‐cytoplasmic shuttling and nuclear activation (Fig 4G). No differences were detected by administering RB5, confirming that its mode of action requires the integrity of the ERK1/2 importin system. However, the mechanism controlling nuclear ERK1/2 activation appears to be radically different for the two importins. In fact, while IPO7 knockdown does not alter ERK1/2 nuclear localization, KPNA2 downregulation significantly increases ERK1/2 nuclear accumulation in a RB5‐independent manner (Fig 4H). Together, these data suggest that RB5 may selectively enhance ERK‐mediated nuclear events by increasing ERK2 binding to KPNA2, thus leading to ERK2 nuclear accumulation. Our data also suggest that KPNA2 is essential for an efficient nuclear exit of ERK proteins, which in physiological conditions occurs when ERK1 and ERK2 become dephosphorylated and inactive (Costa *et al*, 2006).

### RB5‐mediated facilitation of nuclear ERK activity prevents neuronal cell loss in models of neurodegeneration

**Figure 5:**
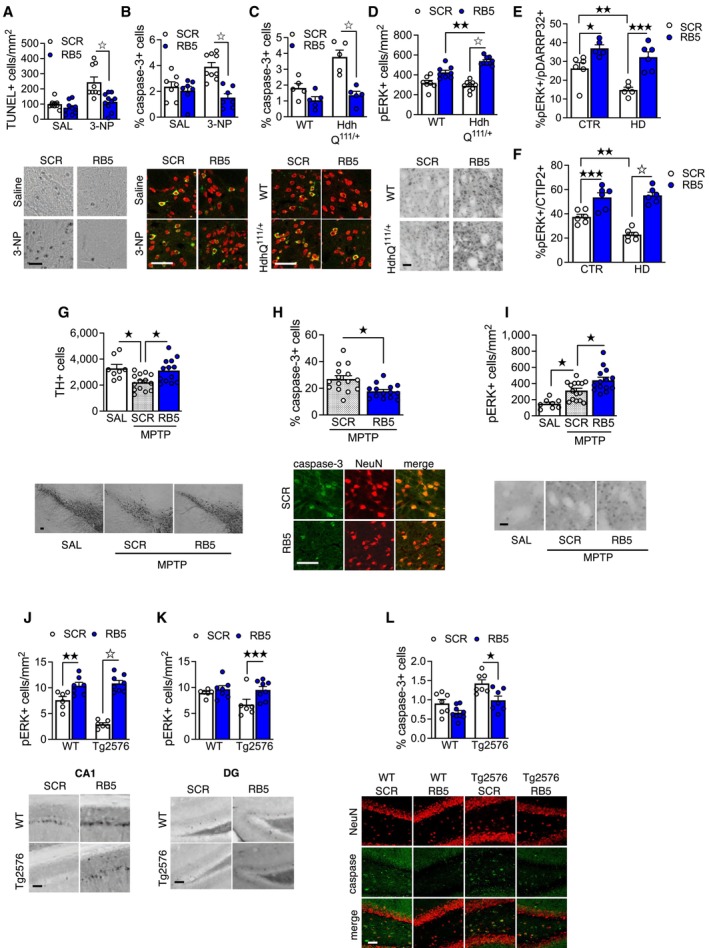
RB5 shows neuroprotective effects in HD, PD and AD mouse models A, B(Bottom panels) Representative images of the TUNEL assay and cleaved caspase‐3 immunofluorescence (red: NeuN, green: cleaved caspase‐3) in the striatum of mice injected either with SCR or RB5 peptide (20 mg/kg, i.p., twice a day, 12 h interval) or with saline or 3‐NP (50 mg/kg, i.p., once a day) for seven consecutive days (top panels). Mice co‐treated with RB5 and 3‐NP showed reduced apoptotic levels (A) and diminished cleaved caspase‐3 levels (B). Data were obtained from *n* = 7–10 mice per group.C, D(Bottom panels) Representative images of cleaved caspase‐3 and pERK immunofluorescence in WT and Hdh^Q111/+^ mice injected with RB5 or SCR (20 mg/kg i.p.) for 8 days (top panels). Hdh^Q111/+^ mice treated with RB5 showed diminished caspase‐3 levels (C) and a greater enhancement of ERK phosphorylation compared to WT (D). Data were obtained from 5 to 9 mice per group.E, FRB5 rescued ERK activation in iPSCs‐derived HD MSNs. HD cells and isogenic controls (CTR) underwent differentiation and were treated with RB5 or SCR 50 μM for 30 min. Cells were processed for immunofluorescence for p‐ERK1/2 and CTIP2 (F) or DARPP‐32 (E). Data were obtained from two HD and two isogenic control lines (*n* = 5–6 slides per condition). (E) p‐ERK1/2 was upregulated in the presence of RB5 in both HD and control DARPP‐32+ neurons. pERK1/2 levels were reduced in HD neurons, which was rescued by RB5 treatment (F) RB5 induced ERK activation in both control and HD CTIP2+ neurons. p‐ERK1/2 levels were reduced in HD lines but were rescued by RB5 treatment.G–I(Bottom panels) Representative images showing TH, TUNEL, and cleaved caspase‐3 after co‐treatment with MPTP (20 mg/kg i.p.) and RB5 or SCR (20 mg/kg i.p.) for 4 days. RB5 showed a protective effect on DA cells of the SN from MPTP toxicity (G, top panel) and a concomitant decrease of cleaved caspase‐3‐positive cells (H, top panel). MTPT caused an increase in ERK activation in both SCR and RB5‐treated mice in the dorsal striatum (I, top panel) that was further enhanced by RB5 treatment (*n* = 8–15 mice per group).J–L(Bottom panels) Representative images showing pERK in CA1 and DG and cleaved caspase‐3 in DG of Tg2576 mice treated either with RB5 or SCR (20 mg/kg i.p.) for 7 days. RB5 enhanced ERK phosphorylation in both CA1 (J, top panel) and DG (K, top panel). In addition, RB5 reduced cleaved caspase‐3 levels in Tg2576 mice (L, top panel). Data were obtained from *n* = 6–9 mice per group. Data information: Scale bars: 50 μm. Results show mean ± s.e.m. ^☆^ *P* < 0.0001, ^★★★^ *P* < 0.001, ^★★^ *P* < 0.01, ^★^ *P* < 0.05. A full statistical analysis is reported in Appendix Table S1. Source data are available online for this figure.

**Figure EV4:**
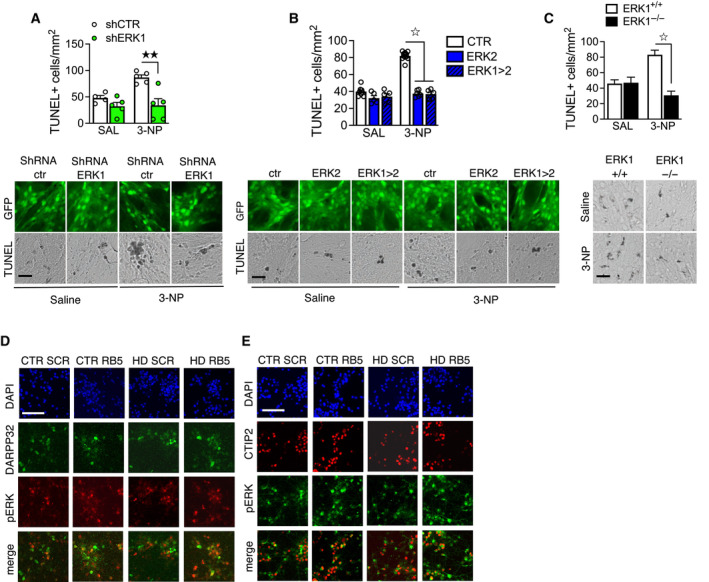
ERK1 loss and ERK2 overexpression protect striatal neurons from 3‐NP‐induced apoptosis A, B(Bottom panels) Representative images of the TUNEL assay in mice with *in vivo* striatal knockdown of ERK1 (A) or overexpression of ERK2 and ERK1 > 2 (B). (top panels) TUNEL assay performed after 21 days of sub chronic treatment with 3‐NP (50 mg/kg, i.p. once a day) or saline. shRNA ERK1 protected striatal neurons from 3‐NP‐induced apoptosis (A, top panel). Similarly, ERK2 or ERK1 > 2 overexpression protected against 3‐NP‐induced apoptosis (B, top panel). Data were obtained from *n* = 4–10 mice per group.C(Bottom panel) Representative images of the TUNEL assay in the striatum of ERK1^−/−^ mice injected either with saline or 3‐NP (50 mg/kg, i.p. once a day) for 21 days. (top panel) ERK1^−/−^ mice were protected against the 3‐NP‐induced striatal neurotoxicity. Data were obtained from *n* = 9–13 mice per group.DRepresentative images of pERK1/2 and DARRP‐32 immunofluorescence in HD lines and isogenic controls.ERepresentative images of pERK1/2 and CTIP‐2 in HD lines and isogenic controls. Data information: Scale bars: 50 μm. Results show a mean ± s.e.m. ^☆^ *P* < 0.0001, ^★★^ *P* < 0.01. A full statistical analysis is reported in Appendix Table S1. Source data are available online for this figure.

Having identified a mechanism facilitating ERK signaling *in vivo*, we tested the possibility that a selective enhancement of nuclear ERK activity could be neuroprotective in experimental models of neurodegenerative diseases. First, we examined two mouse models of striatal degeneration and HD. Mice with bilateral ERK1 knockdown in the striatum by shRNA (Fig EV4A), bilateral overexpression of ERK2 or ERK1 > 2 in the striatum (Fig EV4B) or ERK1 KO mice (Fig EV4C) were chronically treated with the neurotoxin 3‐nitropropionic acid (3‐NP; Fernagut *et al*, 2002). 3‐NP‐induced apoptosis, measured with TUNEL, was reduced in all groups and comparable with saline‐treated animals. Importantly, WT animals co‐treated with RB5 and 3‐NP had significantly reduced neuronal death (Fig 5A and B). In 10 month‐old Hdh^Q111/+^ mice, a late‐onset model of HD (Wheeler *et al*, 2000), RB5 also attenuated striatal neuronal degeneration (Fig 5C) while concomitantly enhancing ERK activity (Fig 5D).

To confirm the role of ERK1/2 signaling in modulating striatal neurodegeneration in a human model of HD, we took advantage of an induced pluripotent stem cell (IPSCs) line differentiated into SPNs (Figs 5E and F, and EV4D and E). Remarkably, using two distinct markers of striatal differentiation, DARPP‐32 (Figs 5E and EV4D) and CTIP2 (Figs 5F and EV4E) to identify SPNs, we found that pERK1/2 levels were significantly reduced in HD SPNs in comparison with an isogenic WT line. Importantly, RB5 was able to enhance pERK1/2 levels both in WT and HD striatal cells, suggesting that the neuroprotective effect of RB5 via enhanced ERK signaling was conserved between mouse models and human cells.

We further investigated whether the neuroprotective effect induced by ERK potentiation in models of HD could also apply to other neurodegenerative disorders. We found that RB5 prevented tyrosine hydroxylase (TH) loss in the substantia nigra pars compact (SNc, Fig 5G) and counteracted apoptotic cell death (Fig 5H) in the 1‐methyl‐4‐phenyl‐1,2,3,6‐tetrahydropyridine (MPTP) mouse model of PD (Carta *et al*, 2013; Lecca *et al*, 2015). Interestingly, when we measured the signaling consequences, we observed an upregulation of pERK1/2 in the dorsal striatum of MPTP‐treated animals vs saline, further enhanced by the RB5 treatment (Fig 5I). This response is not entirely surprising since it is likely to be a reactive response to the neurotoxic insult, as previously reported in nonhuman primate MPTP models (Bezard *et al*, 2005).

Finally, in the early stages (7 months old) of disease progression in the Tg2576 model of AD, a 7‐day RB5 treatment restored ERK phosphorylation in the hippocampal CA1 and dentate gyrus (Fig 5J and K) and significantly reduced the preapoptotic phenotype measured by caspase‐3 (Fig 5L).

In conclusion, a general enhancement of ERK activity in the brain results in neuroprotection across different models of neurodegeneration.

### RB5 enhances cognition in normal mice and in models of neurodegeneration

ERK‐dependent signaling also plays a major role in cognition. ERK2 augmentation positively modulates synaptic plasticity and memory (Mazzucchelli *et al*, 2002; Silingardi *et al*, 2011), but the evidence is limited to a single mouse model in which ERK1 has been ablated from embryogenesis with consequent confounding developmental effects. Therefore, we tested whether pharmacological potentiation of nuclear ERK signaling in adults causes significant synaptic and behavioral changes associated with cognitive enhancement in WT mice and disease models.

**Figure 6:**
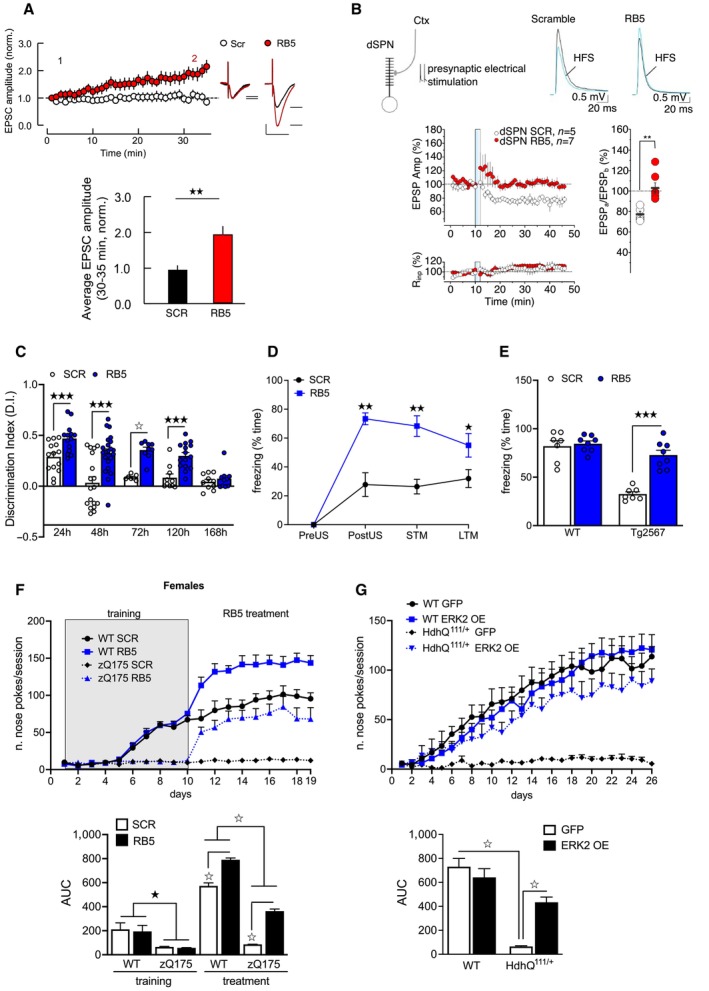
ERK enhancement facilitates both synaptic plasticity and memory and ameliorates cognitive deterioration Intracellular dialysis of the RB5 peptide increased evoked synaptic currents at SC‐CA1 synapses, whereas the control peptide (SCR) had no effect. (Top panel) Time course of minute‐average EPSC amplitude at SC‐CA1 synapses in whole‐cell patch clamp recordings with RB5 (50 μM) or SCR peptide (50 μM) – (values normalized to the mean EPSC amplitude during the first minute of recording) (insets). Representative current traces from 3 min (black, 1) and 33 min time points (red, 2). (bottom panel) Average EPSC amplitude at 30–35 min, normalized to the first minute of recording. RB5 (*n* = 12 cells, four animals), SCR (*n* = 8 cells, four animals). Scale bars: 40 pA and 50 ms.(Top left) Schematic representation of electrically stimulated glutamatergic cortical inputs to dSPNs. (Top right) Superimposed averaged recordings (10 traces) before and after the delivery of the HFS protocol. (bottom left) The HFS protocol (vertical bar) failed to induce plasticity in dSPNs when the RB5 peptide (50 μM) was applied intracellularly. The specificity of action of RB5 was confirmed because the control peptide (50 μM) had no effect on plasticity induction. Data are shown as a time course (mean ± s.e.m.) of normalized EPSP amplitudes and normalized Rinp. (bottom right) Scatterplot summary of the ratios of synaptic responses after (a; 20–30 min) and before (b; 5 min) the HFS.RB5 enhanced NOR memory formation and maintenance for up to 5 days. The Discrimination Index (D.I.) of the RB5 group (20 mg/kg, i.p.) was higher than that of SCR group at 24 h. At 48 h D.I. of the SCR group dropped below chance level, while D.I. of the RB5 group was greatly above it. At 72 h, D.I. of the RB5 group remained higher than the SCR and above chance level. At 120 h, D.I. of RB5 group was still higher than the SCR and above the chance level. By 168 h, D.I. of the RB5 group reached chance level, indistinguishable from that of the SCR group (*n* = 7–22 mice per group).RB5 enhanced acquisition of contextual fear memory in rats. Awake rats were infused bilaterally via an indwelling steel cannula aimed at the dorsal CA1 with either 2 mg/ml SCR peptide (*n* = 6) or RB5 (*n* = 5) 20 min prior to conditioning. STM and LTM were assessed by measuring the conditioned freezing behavior during a 2 min recall test 3 h and 2 days after training, respectively. RB5 administration had a profound effect on the freezing behavior of the rats. The freezing behavior of RB5 rats was increased compared to SCR rats in the Post US, STM, and LTM tests.E) RB5 enhanced the acquisition of contextual fear memory in the Tg2576 mouse model of Alzheimer's Disease. 7 ‐month‐old Tg2576 mice and WT mice were treated for 1 h with either SCR (*n* = 7) or RB5 (20 mg/kg, i.p., *n* = 8) before contextual fear conditioning and tested for memory retention 24 h later. Contextual fear memory impairment in Tg2576 mice was fully rescued by RB5 treatment.(Top panel) WT and zQ175 mice underwent daily 20‐min nose poke training for 10 consecutive days (days 1–10) on a simple fixed ratio (FR1) schedule of reinforcement. Starting from day 11, mice received one administration of RB5 (20 mg/kg. i.p.) 1 h before the nose poke training. RB5 treatment improved performance in both WT and zQ175 females. zQ175 RB5 (*n* = 7) zQ175 SCR (*n* = 8) WT RB5 (*n* = 7) WT SCR (*n* = 8). (Bottom panel) Area under the curve (AUC) analysis.(top panel) WT and Hdh^Q111/+^ mice injected at 2 months of age with either LV‐GFP or LV‐overexpressing ERK2 (ERK2 OE) underwent nose poke training at 18 months of age. Untreated Hdh^Q111/+^ mice differed from the other three groups and poorly performed in this task, while Hdh^Q111/+^‐treated mice were indistinguishable from the WT controls. Data were obtained from WT GFP (*n* = 10), WT ERK2 OE (*n* = 9), Hdh^Q111/+^ GFP (*n* = 9), and Hdh^Q111/+^ ERK2 OE (*n* = 8). (bottom panel) AUC analysis over 26 days. Data information: Results show a mean ± s.e.m. ^☆^ *P* < 0.0001, ^★★★^ *P* < 0.001, ^★★^ *P* < 0.01, ^★^ *P* < 0.05. A full statistical analysis is reported in Appendix Table S1. Source data are available online for this figure.

Using whole‐cell patch clamp recordings in acute slices at Schaffer collateral (SC)‐CA1 synapses, the effect of RB5 on synaptic transmission, in which ERK signaling plays a prominent role (English & Sweatt, 1997), was investigated. Intracellular dialysis of RB5 induced a gradual but sustained increase in the amplitude of excitatory postsynaptic currents (EPSCs) evoked in CA1 pyramidal neurons by stimulation in the *stratum radiatum* (Fig 6A). RB5 is therefore able to positively modulate synaptic efficacy in a tetanus‐independent manner in the hippocampus.

ERK signaling is strongly implicated in forms of striatal long‐term potentiation (LTP) and long‐term depression (LTD; Cerovic *et al*, 2015; Trusel *et al*, 2015). High‐frequency stimulation (HFS) of somatosensory cortex layer V induces adenosine A1 receptor (A1R)‐mediated LTD at corticostriatal synapses on direct spiny projection neurons (sSPNs) that is suppressed upon concurrent activation of postsynaptic dopamine D1R or downstream signaling cascades, including ERK (Trusel *et al*, 2015; Spigolon *et al*, 2018). When RB5 peptide was administered via a patch pipette to the dSPNs, the stimulation protocol failed to induce LTD (Fig 6B). These data indicate that selective potentiation of nuclear ERK signaling is sufficient to modify synaptic efficacy in two distinct brain circuitries.

The clear effect of RB5 on synaptic plasticity in brain slices prompted us to investigate whether cognition could be improved in normal rodents. Remarkably, a single systemic administration of RB5 in mice enhanced performance in the NOR test (d'Isa *et al*, 2014), with NOR memory persisting for over 120 h compared to < 48 h in controls (Fig 6C) as evidence of augmented consolidation. Using a contextual fear conditioning protocol in rats that has been used to successfully distinguish hippocampal‐dependent short‐term and long‐term memory (Lee *et al*, 2004), a single bilateral infusion of RB5 into the dorsal hippocampus 20 min prior to conditioning markedly enhanced fear memory acquisition (post US), short‐term memory (3 h) and long‐term memory (48 h; Fig 6D). These data confirm that a single administration of a selective nuclear ERK‐enhancing drug improves the acquisition and retainment of short‐ and long‐term memory in both normal mice and rats.

Early cognitive impairments are often observed at the prodromal stages of most neurodegenerative disorders, including AD and HD. We investigated whether a single systemic RB5 injection could improve early cognitive deficits in the Tg2576 mouse model (D'Amelio *et al*, 2011). Indeed, RB5 fully prevented the memory impairment in contextual fear conditioning at 7 months of age (Fig 6E). This confirms the potential therapeutic validity of selective enhancement of nuclear ERK to prevent early memory deficits. Importantly, it is worth noting that the observed rapid, RB5‐ mediated, cognitive amelioration in the Tg2576 mouse is unlikely to be linked to the anti‐apoptotic action described in Fig 5L, but rather to a direct synaptic effect of the drug.

**Figure EV5:**
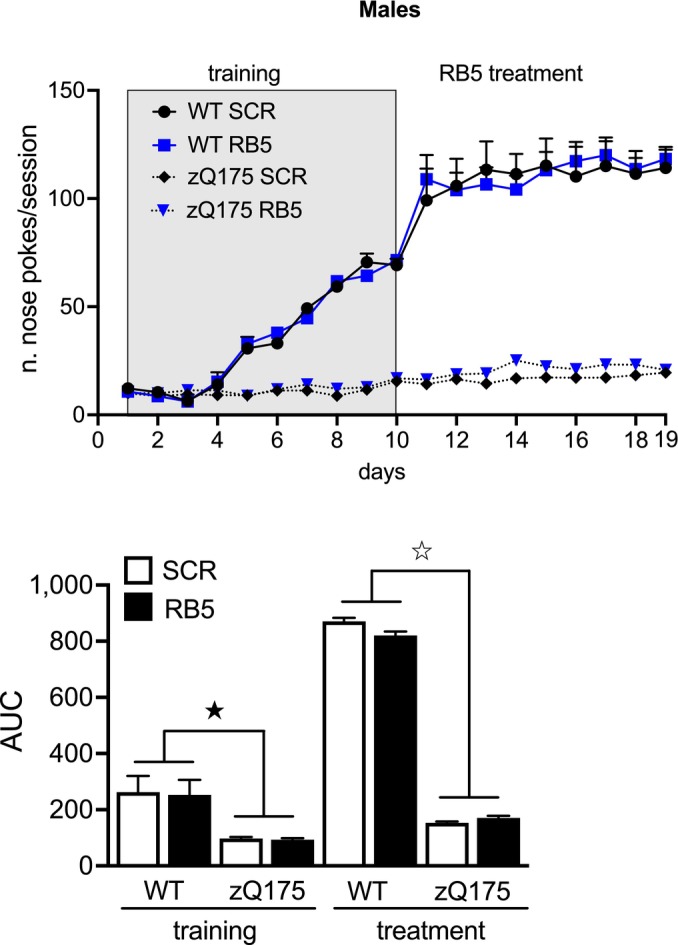
RB5 administration does not counteract learning deficits in zQ175 males (Top panel) A clear impairment in zQ175 male mice was detected from day 1 to day 10 of the FR1 training. However, RB5 administration did not show any effect on learning. (bottom panel) AUC analysis. WT SCR *n* = 8; WT RB5 *n* = 8; zQ175 SCR *n* = 10; zQ175 RB5 *n* = 9. Data information: Results show a mean ± s.e.m. ^☆^ *P* < 0.0001, ^★^ *P* < 0.05. A full statistical analysis is reported in Appendix Table S1. Source data are available online for this figure.

To investigate whether RB5 could also improve procedural learning deficits associated with HD, we used the relatively early onset zQ175 model (Heikkinen *et al*, 2012; Menalled *et al*, 2012). Seven‐month‐old animals were trained in a procedural task (FR1 operant conditioning schedule of reinforcement) for 10 days (Yhnell *et al*, 2016a), then injected daily with either RB5 or scrambled peptide 1 h before testing on subsequent days (Fig 6F). After training, both male and female zQ175 showed deficits in nose poke responses. However, 9 days of RB5 treatment significantly enhanced responses in both WT and zQ175 females. Remarkably, the RB5‐treated zQ175 females performed at the same level as scrambled peptide‐treated WT mice. In contrast, RB5 treatment did not alter responses in zQ175 males (Fig EV5).

Thus, RB5 enhanced cognition and rescued procedural learning deficits selectively in zQ175 females, providing an acute beneficial effect that is independent from the neuroprotective effect. We next investigated whether a long‐term enhancement of ERK signaling in the striatum could prevent cognitive decline in HD mice in a late‐onset HD mouse model, Hdh^Q111^ (Wheeler *et al*, 2002; Yhnell *et al*, 2016a). 2‐month‐old, presymptomatic mice were bilaterally injected with a LV expressing ERK2 or GFP‐alone control into the dorsal striatum to sustain enhancement of signaling over 16 months. At 18 months of age, mice were trained on the same operant FR1 schedule of reinforcement. WT controls and Hdh^Q111/+^ mice expressing ERK2 learned the task, while GFP‐treated HdhQ^111/+^ mice did not (Fig 6G).

Together, these data indicate that pharmacogenetic potentiation of brain ERK signaling can improve cognition in at least certain learning and memory tests and in distinct mouse models of neurodegenerative disorders.

### ERK signaling potentiation prevents neurodegeneration in models of Huntington's disease

**Figure 7:**
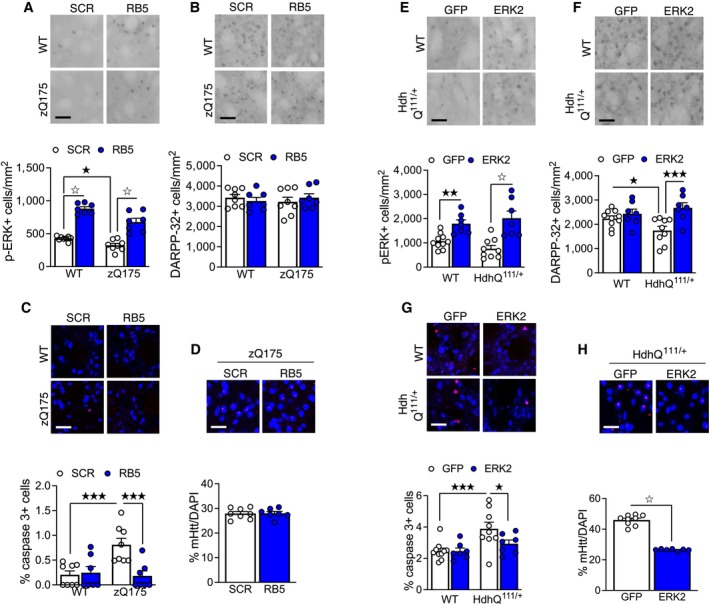
Effect of pharmaco‐genetic ERK manipulation in HD mouse models A–DAfter operant conditioning, brains from zQ175 females were processed for histological analysis (WT SCR *n* = 8, WT RB5 *n* = 7, zQ175 SCR *n* = 8, zQ175 RB5 *n* = 7). (A, bottom panel) RB5 induced ERK activation in both WT and zQ175 mice. (B, bottom panel) DARPP‐32 levels were unchanged in 7‐months old females. (C, bottom panel) Cleaved caspase‐3 levels were normalized by RB5 treatment in zQ175 females. (D, bottom panel) RB5 treatment did not change mutant huntingtin (mHtt) levels in zQ175 females. (A–D, top panels) Representative images. Blue: NeuN (C), DAPI (D). Red: cleaved caspase‐3 (C), mHtt (D).E–HAfter operant conditioning, brains from Hdh^Q111/+^ mice were processed for histological analysis (WT GFP *n* = 10, WT ERK2 *n* = 7, Hdh^Q111/+^ GFP *n* = 9, Hdh^Q111/+^ ERK2 *n* = 7). (E, bottom panel) ERK2 overexpression upregulated p‐ERK1/2 in both WT and mutant mice. (F, bottom panel) ERK2 overexpression rescued DARPP‐32 levels in Hdh^Q111/+^ mice (G, bottom panel) Cleaved caspase‐3 levels were reduced by ERK2 overexpression. (H, bottom panel) Hdh^Q111/+^ mice injected with ERK2‐GFP showed reduced levels of mHtt in comparison with Hdh^Q111/+^ mice injected with GFP. (E‐H, top panels) Representative images. Blue: NeuN (G), DAPI (H). Red: cleaved caspase‐3 (G), mHtt (H). Data information: Scale bars: 50 μm. Data are expressed as mean ± s.e.m. ^☆^ *P* < 0.0001, ^★★★^ *P* < 0.001, ^★★^ *P* < 0.01, ^★^ *P* < 0.05. A full statistical analysis is reported in Appendix Table S1. Source data are available online for this figure.

To evaluate a potential disease‐modifying effect of our therapeutic approaches, histological analysis was performed on postmortem brains from the same cohorts of Hdh^Q111/+^ and zQ175 mice. We confirmed that pERK1/2 levels, significantly reduced in 7‐months‐old zQ175 females, were enhanced in both zQ175 females and WT controls treated with RB5 for 9 days (Fig 7A), with an effect somewhat more pronounced in the WTs. Considering the early stage of the disease progression, the SPN marker DARPP‐32 was not reduced in the mutant striata (Fig 7B). However, mutant huntingtin (mHtt) aggregates were already present in the mutants (Fig 7D), but undetectable in WT animals. The relatively short RB5 treatment was insufficient to attenuate the mHtt aggregation in the mutants; however, we detected a very significant reduction of the preapoptotic marker caspase‐3 (Fig 7C) in the RB5‐treated mutants, suggesting that a short RB5 treatment had an initial disease‐modifying effect besides the pronounced cognitive enhancement.

Finally, we analyzed 18‐month‐old HdhQ111 brains from mice injected with LVs expressing either ERK2 or a control GFP construct. ERK2 overexpression significantly enhanced pERK1/2 in the striatum of both genotypes, with a stronger effect in the mutants (Fig 7E), which also showed lower pERK levels in comparison with the WTs, an effect also found in the differentiated human IPSCs (see Fig 5E and F). Remarkably, we observed a significant reduction of DARPP‐32 positive cells that was rescued by ERK2 overexpression (Fig 7F). Similarly, caspase‐3 levels in the ERK2‐treated mutants were similar to those observed in WTs (Fig 7G). Finally, ERK2 overexpression reduced mHtt accumulation by 43% in the mutants (Fig 7H).

Altogether, these data establish that ERK‐selective positive modulation in the striatum not only prevents cognitive deterioration in HD mutant mice but may also provide some long term, potentially disease modifying, effects at the neuropathological level.

## Discussion

In patients affected by AD, PD, or HD, the molecular processes may take decades to manifest in the earliest behavioral symptoms, which occur without major anatomical alterations or neuronal loss. Early intervention is the key to preventing synaptic failure associated with cognitive deterioration. This is particularly evident for dementia research; in recent AD trials aimed at preventing αβ amyloid accumulation, molecular outcomes were not matched by attenuation of cognitive symptoms (Sala Frigerio & De Strooper, 2016). Interventions during the phase when prodromal symptoms appear, such as mild cognitive impairment (MCI), have not yet been fully exploited (Morrison & Baxter, 2012).

We provide initial evidence for a strategy targeting a common mechanism implicated in cell survival and cognitive enhancement early in neurodegenerative pathogenesis. With its role in learning and memory (Brambilla *et al*, 1997; Atkins *et al*, 1998), the ERK pathway has been intensely scrutinized as a therapeutic target for psychiatric and neurological disorders, largely via MEK inhibitors and in a few cases, genetic manipulations in the mouse (Fasano & Brambilla, 2011). Both approaches have significant limitations and, in some cases, generate conflicting results. On one hand, MEK inhibitors can only demonstrate a permissive role for the pathway (“is ERK signaling necessary?”) but preclude any investigation of the potential instructive role (“is ERK signaling sufficient?”). On the other hand, genetic models selectively modulate the expression of a key pathway component either early in development (global manipulation) or for an extended period in the adult, and whose effects may be confounded by cellular adaptations and compensatory mechanisms (Vithayathil *et al*, 2017; Eblen, 2018).

Circumventing these limitations, we used two complementary approaches *in vivo* in the adult: selective ERK1 or ERK2 gene inactivation and a novel nuclear ERK‐enhancing cell‐penetrating peptide. First, selective ERK1 and ERK2 knockdowns using viral vectors removed each protein independently in the brain, without developmental effects. Second, the cell‐penetrating peptide allowed us to determine whether a selective nuclear ERK enhancement could impact cell survival, synaptic plasticity, and memory.

We have unraveled the details of the cellular mechanisms by which ERK1 and RB5 regulate access to the nucleus of ERK2 and, by consequence, modulate global nuclear signaling. Our working model indicates that the unique N‐term domain of ERK1 impedes ERK2 binding to KPNA2/7, thus delaying the latter kinase to be transported though the NPC and released into the nucleus, where would exert its function. This is consistent with all our *in vitro* and *in vivo* observations, most notably with the RB5 antagonistic effect on ERK1‐KPNA2 binding, which facilitates ERK2‐KPNA2 interactions.

In recent years, increasing evidence supports the notion that derangements in the nucleocytoplasmic transport machinery and mislocalization of its key components may be implicated in neurodegenerative processes, including HD, PD, and AD (Hutten & Dormann, 2020; Ding & Sepehrimanesh, 2021). In general, it is highly believed that the nuclear barrier in affected neurons becomes dysfunctional largely due to alterations in NPC permeability. However, it has been shown in HD models, that also key components of the Ran‐mediated transport system, including Ran itself and RanGAP, are affected and downregulated in the nucleus, possibly leading to mis‐localization of mHtt and other pathogenic proteins (Liu *et al*, 2015; Gasset‐Rosa *et al*, 2017; Grima *et al*, 2017). Interestingly, in cellular models of AD, KPNA2 has been found to be mislocalized and, among many other proteins, sequestered in amyloid‐like aggregates (Lee *et al*, 2006; Olzscha *et al*, 2011).

KPNA2, as other importinα members, is a multifunctional protein involved in transcriptional and epigenetic regulation, besides its role in nuclear transport (Oka & Yoneda, 2018). Importantly, KPNA2 is upregulated in several tumor cell lines and its levels correlates with the oncogenic potential by promoting proliferation, cell growth, migration, invasion, and tumor formation. On the contrary, down‐regulation of KPNA2 may suppress tumor‐promoting cellular phenotypes (Han & Wang, 2020). Proteomic analysis on lung adenocarcinoma cells overexpressing KPNA2 revealed that an enhanced interaction between vimentin and KPNA2 leads to the recruitment of phosphorylated ERK in the cytoplasm (Wang *et al*, 2015). Whether such mechanism apparently mediated by vimentin either prevents or facilitates ERK nuclear translocation remains unclear. Our own data suggest a significantly different scenario in which the N‐term domain of ERK1 selectively binds to KPNA2. The disruption of that binding facilitates ERK2 interaction with KPNA2, nuclear translocation and enhanced signaling. Interestingly, ERK1 binding to KPNA2 also alters KPNA2 cellular localization, suggesting that this interaction may impact on the functions of KPNA2 in nuclear transport and gene regulation.

The role of KPNA7 in cellular functions is still poorly understood, besides its role in reproduction in mice and other species (Hu *et al*, 2010). However, recent evidence indicates that loss of function mutations of KPNA7 in patients affected by neurodevelopmental disorders significantly reduced the ability of KPNA7 to transport cargoes across the nuclear membrane (Paciorkowski *et al*, 2014; Oostdyk *et al*, 2020).

ERK1 KO mice show potentiated ERK2‐mediated signaling, which enhances neuronal plasticity and memory (Mazzucchelli *et al*, 2002; Silingardi *et al*, 2011). Here, we confirmed that the bidirectional modulation of the ERK2/ERK1 ratio impacts global ERK signaling and alters cell survival in wild‐type mice and neurodegeneration models. Critically, selective activation of nuclear ERK signaling not only enhances memory in wild type rats and mice, but also rescues early cognitive deficits in an AD mouse model and improves cognitive deterioration in two HD models. We believe that the effect on memory is a primary phenomenon, independent from the attenuation of neuronal loss in models of AD, HD, and PD. However, a sustained ERK2 overexpression in the striatum prevents memory decline and attenuates caspase 3 staining and mHtt accumulation. This finding strongly suggests that, at least in HD models, ERK signaling potentiation has dual beneficial long‐term effects. This is consistent with a well‐established role for ERK downstream to BDNF–TrkB receptor activation in the striatum (Cunha *et al*, 2010; Bowles *et al*, 2012) and ERK's neuroprotective role in counteracting mHtt toxicity in HD cellular models. In cell lines expressing inducible mHtt, ERK is activated upon mHtt challenge and elicits pro‐survival responses (Apostol *et al*, 2006). Moreover, in the STHdh^Q111+^ immortalized cellular model of HD, MEK inhibition abolishes the protective effects of BDNF (Gines *et al*, 2010). Finally, the transcription factor Elk‐1, target of ERK1/2, contributes to neuroprotection in STHdhQ^111+^ cells and R6/1 and R6/2 mice (Anglada‐Huguet *et al*, 2012). Thus, evidence from both cultured cells and animal models indicates that ERK signaling, which is also involved in glutamate excitotoxicity, integrates glutamatergic and BDNF signaling (Lievens *et al*, 2005; Huang *et al*, 2010; Ribeiro *et al*, 2011). Our own evidence using RB5 supports and extends this notion and may provide a valid therapeutic approach for HD patients instead of a direct administration of BDNF (or therapeutics modulating its action) which has not been successful yet (Simmons, 2017). It is however important to state that ERK modulation may not be involved in the primary pathological process but is rather a compensatory response to counteract mHtt toxicity. This is also consistent with the notion that basal ERK activity may be different in HD models, depending on the type of mHtt mutation, age, brain region, and cell type considered (Bowles & Jones, 2014). Our own data in two different HD models indicate that indeed basal ERK activity is only moderately reduced, at least at the time points studied, and RB5 treatment or ERK2 overexpression significantly ameliorate cellular and behavioral phenotypes. On the contrary, in our human IPSCs‐based model of terminally differentiated striatal cells, ERK activity appears to be not only significantly downregulated at the basal level but also strongly stimulated by RB5.

An additional interesting aspect to be considered in HD models is that behavioral training or environmental enrichment (EE) can significantly delay cognitive decline (Du *et al*, 2012; Curtin *et al*, 2015; Yhnell *et al*, 2016b), and these effects are sex‐biased, with females better responding to treatment (Du *et al*, 2012). While the sex effect has not been fully confirmed in HD patients, the “therapeutic” effect associated with behavioral training/EE is consistent with a prominent role of epigenetic mechanisms in overcoming the expression of mutant huntingtin. RB5 ability to mimic the same epigenetic mechanisms provides an unprecedented opportunity to develop novel pharmacological approaches for Huntington's disease as well as for the cognitive enhancement of the healthy population.

In AD, early reports indicate that ERK signaling components, including ERK1/2 and MEK1/2, are upregulated in postmortem brains (Ferrer *et al*, 2001a,b; Pei *et al*, 2002). However, later evidence indicates that ERK activation is largely restricted to astroglial cells in the early stages of AD, followed by the loss of active ERK in advanced AD and limited activity in neuronal populations (Webster *et al*, 2006). These findings have fueled speculation that ERK signaling may contribute to pathogenesis via a mechanism in which αβ oligomers activate ERK, leading to tau phosphorylation and further αβ amyloid accumulation (Harris *et al*, 2004; Kirouac *et al*, 2017). However, ERK does not phosphorylate tau *in vivo* (Noel *et al*, 2015). Moreover, intrahippocampal injection of αβ oligomers rapidly downregulates ERK phosphorylation, thus correlating with memory impairments (Faucher *et al*, 2015). Although early evidence suggested that ERK2 activity in the Tg2576 AD model was attenuated only at 20 months of age (Dineley *et al*, 2001), here we show significantly diminished ERK phosphorylation at 7 months of age that is rescued by ERK‐enhancing treatment. Our interpretation is that ERK signaling may respond to αβ amyloid toxicity as a compensatory mechanism rather than being part of the pathogenic process. As an indirect confirmation of our hypothesis, in a recent paper, Evans *et al* observed an improvement in cognitive performance combined with a reduction of Aβ pathology in the hippocampus of aged Tg2576 mice treated with an ACE2 activator. This effect was associated with increased glutamate receptor signaling and ERK potentiation (Evans *et al*, 2020).

Overall, we demonstrated a novel mechanism that may provide both neuroprotection and cognitive enhancement across models of neurodegeneration involving the selective potentiation of nuclear ERK signaling. Potentiation of nuclear ERK signaling in the brain via KPNA2/7 binding has potential benefits in terms of both enhancing cognition in normal aging and providing protection and cognitive enhancement in the context of major disorders such as AD, PD, and HD.

## Materials and Methods

### Animals

All experiments were conducted according to the European Community guidelines (Directive 2010/63/EU) and previously approved by the Institutional Animal Care and Use Committee (IACUC) protocols (# 477) of the IRCCS‐San Raffaele Scientific Institute and the UK Home Office project license to RB (PPL 30/3036) at Cardiff University. 2‐month‐old CD1 male mice (Charles River) were used for immunohistochemical analysis (time course, dose‐response, and mass spectrometry) and the Novel Object Recognition (NOR) test. 2‐month‐old C57BL/6 male mice (Charles River) were used for LV injections, *ex vivo* analysis, immunohistochemistry (IHC), and immunofluorescence (IF). 3‐month‐old C57BL/6 male mice were used for MPTP intoxication. ERK1 KO males and littermate controls, generated as previously described (Mazzucchelli *et al*, 2002), were used for *ex vivo* analysis. Heterozygous Hdh^Q111/+^ knock‐in mice (Jax1, Bar Harbor, Maine, U.S.A.) were bred in‐house on a C57BL/6J background and used for operant conditioning, IHC and IF. Heterozygous 7‐month‐old zQ175 knock‐in mice carrying approximately 180 CAG repeats (CHDI‐ 81003003, Psychogenics, Inc. Tarrytown, NY) and 7‐month‐old Tg2576 transgenic male mice (C57BL6 and SLJ mix background, Taconic Biosciences) were used for IHC, IF, Contextual fear conditioning (CFC), and operant conditioning. Lister‐Hooded male rats (280–350 g) were purchased from Harlan Laboratories for CFC.

### Lentiviral vectors production

Generation and production of Lentiviral (LV) constructs for RNAi of ERK1 and ERK2 (both in shRNA and mir RNA configuration), as well as LV overexpressing ERK1, ERK2, and their swapped counterparts have been previously described (Indrigo *et al*, 2010; Orellana *et al*, 2012). Briefly, third generation nonreplicative LVs were modifications of the originally described backbone (Follenzi *et al*, 2000). LVs have been prepared in HEK 293T cells with a final titer > 10^9^ T.U (transforming units). Cells and mice have been transduced at multiplicity of infection (moi) > 5.

### Mouse embryonic fibroblasts (MEF) preparation and LV infection

MEF cultures were prepared from wild‐type E13.5 embryos obtained from C57Bl/6 mice as previously described (Indrigo *et al*, 2010). 1.25 × 10^5^ cells/well were seeded in 6‐well plates. Cells were fixed 3 or 5 days after LV infection in 4% PFA for 25 min at RT, rinsed in 1× PBS and then stored in 70% ethanol at −20°C.

### Neuronal cultures and LV infection

Neuronal cultures were prepared as previously described (Mazzucchelli *et al*, 2002). Briefly, embryonic cultures (E17) were prepared from cortices of WT mice. 2 × 10^6^ cells/well were plated in 6‐well plates onto poly‐L‐lysine coated glass and kept for 10 days in culture medium. On day 3, cells were infected with LVs of interest (moi = 5) and 3, 5 or 7 days later were fixed in 4% PFA for 25 min at RT, rinsed in 1× PBS, and stored in 70% ethanol at −20°C.

### *In‐vivo* LV injections in C57/Bl6 mice

C57/BL6 adult male mice were deeply anesthetized (i.p. 8% Ketamine (Ketavet 100, Intervet) and 4% Xylazine (Rompun 20 mg/ml, Bayer) and secured on a stereotaxic frame (Kopf). Two bilateral LV injections (2 μl each) were made into the dorsal striatum at the following coordinates: site 1: AP +1; L −2.1; DV −2.6; site 2: AP +0.3; L −2.3; DV −2.4; site 3: AP +1; L +2.1; DV −2.6; site 4: AP +0.3; L +2.3; DV −2.4 (mm from Bregma). In rotational behavior experiments, the LV of interest was injected either into the right or left hemisphere according to a randomized design as control LVs, whereas the contralateral side was injected with control LV to minimize the effect of surgery. In the overexpression and knockdown studies followed by TUNEL staining, the LV of interest was injected in both hemispheres. At the end of the surgical procedure, the skin was closed using surgical staples, and the animal was monitored in a single cage until full awakening. Mice were left undisturbed for 2 weeks to allow transgene expression.

### Spontaneous rotational behavior

Two weeks after the LV injection, spontaneous rotational behavior was observed in a square arena, and the number of 180° rotations to either side were measured. Positive values represent net ipsilateral rotations, while negative values represent net contralateral rotations (the number of 180° rotations toward LV‐ injected side – number of 180° rotations toward control side).

### Imaging of dendritic spines

Mice that underwent rotational behavior were perfused at the end of the behavioral test with 4% PFA. Brains were cut using a vibratome at 250–30 mm thickness. Slices were mounted on slides. The analysis of dendritic spine density was carried out using a two‐photon microscope (Ultima IV, Prairie Technology) equipped with a 10 W laser (Chamaleon Ultra 2, Coherent) tuned at 890 nm that delivered about 30 mW to the sample. Imaging was restricted to well‐resolved dendrites, avoiding fields that were too cluttered. Images were acquired with a water immersion lens (Olympus, 60, numerical aperture: 0.95) at a resolution of 512 × 512 pixels with zoom 4 leading to a field of 50.7 × 50.7 mm and a nominal linear resolution of about 0.1 mm per pixel. Selected dendrites were acquired by collecting a Z‐stack with a vertical resolution of 0.75 μm. Images were analyzed with Fiji‐ImageJ.

### TUNEL assay

The detection of apoptosis on cultured cells or tissue sections was carried out using the DeadEnd™ Colorimetric TUNEL System kit (Promega). Cultured cells were fixed in 4% PFA for 15 min at room temperature before undergoing TUNEL staining. Tissue sections were obtained from mice perfused with 4% PFA. Brains were subsequently stored in 30% sucrose overnight and cut into 35 μm‐thick slices using a freezing microtome. Slices were mounted on slides and processed for TUNEL staining. TUNEL was performed following the manufacturer's instructions. Briefly, slides were immersed in PBS for 5 min RT and then incubated for 25 min in a 20 mg/ml Proteinase K solution. Then, the slides were rinsed in 1× PBS, re‐fixed in 4% PFA for 5 min, and rinsed twice with 1× PBS. One hundred milliliters of equilibration buffer were added to each tissue section for 5–10 min followed by 1 h of incubation at 37°C in a humidified chamber with 100 ml of rTdT reaction mix. After that, slides were immersed in 2× SSC solution in a Coplin jar for 15 min and washed twice in PBS 1× for 5 min. The endogenous peroxidase activity was blocked with 0.3% hydrogen peroxide for 5 min. The slides were rinsed twice in 1× PBS for 5 min and incubated for 30 min with Streptavidin HRP solution and finally DAB substrate was added to react for 10 min. A light microscope was used to acquire four representative fields per slide from cultured cells and 3–4 representative slices per mouse from all experimental groups. The number of TUNEL positive cells was counted using ImageJ software.

### Peptide synthesis

A peptide for inhibiting ERK1 (*Mapk3*) protein kinase signaling was designed around the N‐terminal domain of ERK1 and hereafter named RB5. The RB5 peptide and its ineffective Scramble (SCR) version contain a TAT sequence able to translocate the plasma membrane.

RB5 (GRKKRRQRRRPPQGGGGGEPRRTEGVGPGVPGEVEMVKGQPFDV) and SCRAMBLE (GRKKRRQRRRPPRVGPGVPEGVGVAVFGVKEPGQTGDVGPVGE) peptides were custom synthesized by GENECUST EUROPE (Luxembourg).

For all *in vitro* and *in vivo* experiments, batches of 200 mg peptides were highly purified using high‐performance liquid chromatography (HPLC; ≥ 95%). To enhance stability, RB5 was synthesized containing six D‐amino acid isomers in substitution of the native L‐amino acids (underlined including the C‐terminal amino acid) and the acetylated N‐Terminal amino acid. RB5 and Scramble peptides were dissolved in 1× PBS and injected at the appropriate concentrations.

### *Ex‐vivo* system in acute brain slices

Adult C57Bl/6 mice were decapitated after cervical dislocation and brain slices were freshly prepared as previously described in Orellana *et al* (2012) and Papale *et al* (2016). Briefly, the brains were rapidly removed from the skull, submerged in ice‐cold sucrose‐based dissecting solution, and sliced into 200 μm‐thick slices using a vibratome. Striatal slices were subsequently transferred into the brain slice chamber and allowed to recover for 1 h at 32°C, with a constant perfusion of carboxygenated artificial cerebrospinal fluid in the presence of RB5 peptide (0.5–100 μM for IC50 determination). RB5 or scramble inactive peptide (50 μM) were used in combination with 100 μM glutamate to stimulate brain slices into the chamber for 10 min. After a rapid fixation in 4% PFA, slices were rinsed and cryoprotected overnight at 4°C in 30% sucrose solution. On the following day, slices were cut into 18 μm‐thick slices using a cryostat (Leica CM1850), mounted onto SuperFrost Plus slides (Thermo Scientific) and stored at −20°C until processing for immunohistochemistry or immunofluorescence.

### HEK293 cells culture

For Western blot and immunofluorescence analysis, 0.6 × 10^6^ HEK293 cells were seeded in 6‐well plates in the presence of DMEM High Glucose GlutaMax (Thermo Fisher Scientific) containing 10% Foetal Bovine Serum (FBS, Thermo Fisher Scientific) and 100 U/ml penicillin/streptomycin (Thermo Fisher Scientific). For Western blot analysis to assess ERK1/2 and phospho‐ERK1/2 levels, cells were serum starved for 24 h and incubated with RB5 50 μM or FBS 20% for 15 min at 37°C. To assess JNK and phospho‐JNK levels, cells were incubated with RB5 50 μM or anisomycin 5 μM for 30 min at 37°C. For immunofluorescence analysis, cells were serum‐starved for 24 h and incubated with 50 μM RB5 or 20% FBS for 15 min at 37°C.

### Immunofluorescence on HEK293 cells

After rinsing in PBS, cells were fixed in 4% PFA and permeabilized with increasing concentrations of methanol (33, 50, 75, and 95% in PBS, 2 min each at room temperature), followed by 100% methanol for 10 min at −20°C. After rinsing in PBS, coverslips were blocked in PBS with 5% normal goat serum and 0.3% Triton for 1 h at room temperature and incubated overnight at 4°C with the following primary antibodies: anti‐phospho‐p44/42 MAP kinase (Thr202/Tyr204) (1:200, Cell Signaling, #9101) or anti‐ERK2 (K23) (1:50, Santa Cruz Biotechnology, #sc‐153) in PBS with 0.3% Triton. Coverslips were subsequently incubated with AlexaFluor 488‐conjugated goat anti‐rabbit (1:500, Thermo Fisher Scientific) in PBS with 5% normal goat serum and 0.3% Triton and counterstained with DAPI. Images were acquired from 6 to 8 slides per condition using a confocal microscope (Zeiss LSM710) under a 63× objective. The mean fluorescence intensity in the nucleus was calculated automatically by Fiji‐ImageJ software in 8–10 fields per slide.

### Western blot

Cells were collected in ice‐cold lysis buffer (20 mM Tris HCl pH 7.5, 150 mM NaCl, 1% Triton, 1 mM EDTA, complete EDTA‐free protease inhibitor cocktail (Roche), 1 mM sodium orthovanadate, 1 mM sodium fluoride, 1 mM b‐glycerophosphate). Protein concentration was determined with the Biorad DC protein assay. Equal amounts of proteins (20 mg) were boiled in sample buffer, separated by SDS‐PAGE, and transferred to nitrocellulose membranes (Amersham). Membranes were blocked in 3% BSA in TBS for 1 h at room temperature and incubated overnight at 4°C with the following primary antibodies: anti‐phospho‐p44/42 MAP kinase (Thr202/Tyr204) (1:1,000, Cell Signaling Technology #9101), anti‐ERK2 (K‐23) (1:50, Santa Cruz Biotechnology, #sc‐153), anti‐phospho‐SAPK/JNK (Thr183/Tyr185) (1:1,000, Cell Signaling Technology #9251), anti‐SAPK/JNK (1:1,000, Cell Signaling Technology #9252), anti‐GAPDH (1:20,000, Sigma Aldrich #G9545) in 0.2% TBS‐Tween with 3% BSA.

After rinsing in 0.2% TBS‐Tween, membranes were incubated for 1 h at room temperature with goat anti‐rabbit secondary antibody IRDye 800CW (1:15,000, Licor, #926‐32211) in TBS‐Tween 0.2 with 3% BSA. Band detection and densitometry was performed using Odyssey CLx Imaging system (Licor).

### siRNA treatment on HEK293 cells

After 24 h starvation, cells were incubated with 200 nM siRNA KPNA2 or siRNA IPO7 (Mission esiRNA, Sigma Aldrich) for 24 h at 37°C. Cells were subsequently treated with RB5 50 μM RB5 or 20% FBS for 15 min at 37°C before Western blot or immunofluorescence analysis.

### Automated Western blot

An automated Western blot system (Jess, Simple Western) was used to measure ERK1 and ERK2 levels in mouse striata after shRNAs treatment and to measure KPNA2 and IPO7 levels after siRNAs treatment. We followed the manufacturer's method for 12‐230‐kDa Jess separation module. For ERK1 and ERK2, stacking loading time was set to 16 s, sample loading time to 6 s and separation time to 32 min. For KPNA2 and IPO7, stacking loading time was set to 15 s, sample loading time to 9 s and separation time to 25 min.

Protein lysates were mixed with 0.1× Sample Buffer and Fluorescent 5× Master mix (Protein Simple) to reach a concentration of 1 μg/μl (ERK1/2 and IPO7 analysis) or a concentration of 0.04 μg/μl (KPNA2 analysis). This preparation was denatured at 95°C for 5 min. Ladder (Protein Simple) and proteins were separated in capillaries as they migrated through a separation matrix. A proprietary normalization reagent (Protein Simple) was added for 25 min. After blocking in Milk‐free antibody diluent (Simple Western), anti‐ERK1/2 (#4696, Cell Signaling, 1:50 in Milk‐free antibody diluent), anti‐KPNA2 (#ab84440, Abcam, 1:50 in Milk‐free antibody diluent) and anti‐IPO7 (#ab99273, Abcam, 1:50 Milk‐free antibody diluent) were applied for 1 h (anti‐ERK1/2) or 30 min (anti‐KPNA2 or anti‐IPO7). HRP‐conjugated anti‐mouse or anti‐rabbit secondary antibodies (Protein Simple) were applied for 30 min, followed by peroxide/luminol‐S (Protein Simple) detection. Chemiluminescence intensity was calculated automatically by Compass Simple Western software (version 6.1.0, Protein Simple) and normalized over total protein levels.

### *In vivo* dose response and time course of RB5

For the dose response study, 2‐month‐old CD1 male mice were injected with different doses of RB5 (1, 2.5, 5, 10, and 20 mg/kg, i.p.), whereas the control group was injected with 20 mg/kg (i.p.) of SCR peptide. Mice were perfused 1 h later with 4% PFA. For the time course study, mice were injected i.p. with 20 mg/kg of RB5 and perfused at different time points with 4% PFA (1, 3, 6, and 12 h). The control group was injected i.p. with 20 mg/kg of SCR. Brains were postfixed in 4% PFA for 24 h and cryoprotected overnight in sucrose 30% before immunohistochemistry against phospho‐ERK1/2.

### Liquid chromatography and tandem mass spectrometry (HPLC‐MS/MS)

The bioavailability of RB5 in the mouse brain was determined at different time intervals (1, 3, 6, and 12 h) after a single i.p. administration of 20 mg/kg. RB5 was quantified in the mouse brain using HPLC‐MS/MS. Brain samples were homogenized with 1:4 w/v of 50% acetonitrile 5% TFA in water with a homogenizer ultra‐turrax and then centrifuged at 13,000 rpm for 10 min at 4°C. The supernatant was collected and centrifuged at 15,871 *g* for 2 min at 4°C. The supernatant was then collected on ice, extracted using Sep‐Pak cartridges C18, lyophilized and kept at 4°C before HPLC‐MS/MS analysis. Immediately before the analysis, samples were suspended in 100 μl of 0.1% HCOOH in water/8% acetonitrile in auto‐sampler vials.

HPLC‐MS/MS analysis was performed using a system consisting of an Agilent 1200 series HPLC system coupled with an Agilent 6410 Triple Quadruple mass spectrometer. Mass Hunter Workstation v. B.01.03 software was used for data collection and processing (Agilent Technologies, Santa Clara, California, US). The quantification of brain levels of RB5 and Scramble peptides was carried out using an internal standard curve with peptide concentrations ranging from 0.1 to 4 ng/μl. RB5 and Scramble peptides, and the internal standard were separated at room temperature by injecting 10 μl of extracted sample onto a Jupiter C4 300 A analytical column, 2 × 150 mm, 5 μm particle size (Phenomenex, CA). Gradient elution was used for chromatographic separation, using 0.1% formic acid in water as solvent A and acetonitrile as solvent B at a flow rate of 200 μl/min. The elution started with 92% of eluent A and 8% of eluent B maintained for 1 min, followed by a 4 min linear gradient to 75% of eluent B, a 1 min linear gradient to 99% of eluent B, a 2 min isocratic elution, and a 0.5 min linear gradient to 8% of eluent B, which was maintained for 9.5 min to equilibrate the column. The samples were maintained at 4°C in the autosampler.

Peptides were then detected on an Agilent 6410 QQQ mass spectrometer using the following parameters: positive ion mode, 5 kV capillary voltage, cone voltage 500 V, gas flow rate 8 l/min at 350°C, nebulizer gas pressure 40 PSI at 350°C, well time 75 msec and Q1 and Q3 set to unit resolution.

### Immunohistochemistry

Coronal sections from the SNc (40‐μm thick) of MPTP‐treated mice were cut on a vibratome.

Coronal sections from the striatum of wild‐type, zQ175, and Hdh^Q111/+^ (35 μm‐thick) were cut on a freezing microtome and stored in a cryoprotective solution at −20°C.

Immunohistochemistry was performed as described in Papale *et al* (2016). Briefly, 1 h after blocking in 5% normal goat serum and 0.1% Triton X‐100 solution, free‐floating sections were incubated overnight at 4°C with one of the following primary antibodies: anti‐phospho‐p44/42 MAP kinase (Thr202/Tyr204) (1:200, Cell Signaling Technology #9101), anti‐phospho–Elk‐1 (Ser383) (1:100, Cell Signaling #9181), anti‐phospho‐Kv4.2 (Thr607)‐R (1:100, Santa Cruz sc22254‐R), anti‐phospho‐MSK‐1 (1:100 Cell Signaling Technology #9595), anti‐phospho‐MEK1/2 (Ser218/222) (1:100, sc‐7995 Santa Cruz Biotechnology), c‐Fos (1:100, sc‐52 Santa Cruz Biotechnology), tyrosine Hydroxylase (monoclonal 1:1,000, Sigma Aldrich, Italy) and anti‐DARPP‐32 (H3) (1:100, Santa Cruz Biotechnology, #sc‐271111) overnight at 4°C. Sections were then incubated with biotinylated goat anti‐rabbit IgG (1:200, Vector Laboratories) or biotinylated goat anti‐mouse (1:200, Vector Laboratories) for 2 h. The classic avidin‐peroxidase complex (ABC, Vector, UK) protocol was applied, using 3,3′‐diaminobenzidine (DAB, Sigma) as a chromogen.

### Immunofluorescence

Immunofluorescence was performed as described in Papale *et al* (2016). Briefly, 1 h after blocking in 5% normal goat serum, slices were incubated overnight at 4°C with the following primary antibodies: anti‐phospho (Ser10)‐acetylated (Lys14) histone H3 (1:1,000, Millipore), anti‐phospho‐S6 ribosomal protein (Ser235/236) (1:200, Cell Signaling Technology) anti‐phospho‐S6 ribosomal protein (Ser240/244) (1:200, Cell Signaling), cleaved caspase‐3 (1:200, Cell Signaling Technology), anti‐mutant Huntingtin (1:100, Developmental Studies Hybridoma Bank, University of Iowa, #MW8), and NeuN (1:1,000, Millipore). Corresponding secondary antibodies (Alexa Fluor 546 conjugated anti‐mouse (1:200, Thermo Fisher Scientific), Alexa Fluor 488 conjugated anti‐rabbit (1:500, Thermo Fisher Scientific) and Pacific Blue goat anti‐mouse (1:200, Thermo Fisher Scientific) were incubated at room temperature for 1 h. Sections stained for mutant Huntingtin were incubated with DAPI for 20 min. Sections were subsequently mounted with fluorescent mounting medium (DAKO). Single and double‐labeled images (1,024 × 1,024 μm) were obtained at 40× magnification from the striatum using a laser scanning confocal microscope (Leica SP2) equipped with the corresponding lasers and appropriate filter sets to avoid the crosstalk between the fluorochromes.

### IHC and IF image quantification

For *ex‐vivo* immunohistochemistry experiments, images were acquired from the dorsal striatum using a bright‐field microscope (Macro/Micro Imaging System, Leica) under a 40× magnification. Neuronal quantification was performed with ImageJ software by counting the number of phospho‐ERK positive cells in two sections for each sample in three fields per section. The number of positive cells per mm^2^ in RB5‐treated slices was normalized over the scramble controls. The EC50 for pERK was calculated using GraphPad Prism software.

For *in vivo* immunohistochemistry experiments, images were acquired from the dorsal striatum using a Leica DM IRB microscope under a 20× objective. The number of p‐ERK, p‐MSK‐1, p‐ELK, p‐MEK‐1, p‐VG K+, c‐Fos, and DARPP‐32 positive cells was counted in 2–3 consecutive sections per mouse, bilaterally and averaged across the sections using the ImageJ software by a blind investigator to the condition. Estimation of nigral cell loss was assessed by quantifying the number of TH‐immunopositive neurons of the SNc in 360 μm‐spaced sections for each animal.

In *ex‐vivo* and *in‐vivo* immunofluorescence experiments, the number of p‐S6 or p‐AcH3 immunoreactive neurons among NeuN‐positive neurons was counted in 2–3 consecutive striatal sections per mouse in four fields per section. Caspase 3‐positive cells were quantified among NeuN‐positive neurons in each slide and expressed as percentage (ratio between caspase‐3‐positive cells and total NeuN‐positive neurons) for each field acquired through the slide. The number of cells showing mutant Huntingtin inclusions were expressed as a percentage of mutant Huntingtin‐positive cells among DAPI‐positive cells.

### Proximity ligation assay (PLA)

1.5 × 10^6^ HEK293 cells were seeded onto coverslips in 60 mm dishes. 24 h later, transfection was carried out using calcium phosphate method. 0.5 μg of pCMVTNT‐T7‐KPNA1 (Addgene, #26677), 0.5 μg of pCMVTNT‐T7‐KPNA2 (Addgene, #26678), 0.5 μg of pCMVTNT‐T7‐KPNA3 (Addgene, #26679), 0.5 μg of pCMVTNT‐T7‐KPNA4 (Addgene, #26680), 0.5 μg of pCMVTNT‐T7‐KPNA5 (Addgene, #26681), 1.5 μg of pcDNA3.1+/C‐(K)‐DYK‐KPNA6 (Genscript, #OHu29951D), 0.5 μg of pCMVTNT‐T7‐KPNA7 (Addgene, #26683), 5 μg of pcDNA3.1 +/C‐(K)‐DYK‐IPO7 (Genscript, #OHu11630), 0.5 μg pCMV5‐3HA‐RanBP5 (MRC PPU, University of Dundee, #DU7262), 0.5 μg pCMV5‐3HA‐ KPNB1 (MRC PPU, University of Dundee, #DU5332) were mixed with one of the following plasmids: 0.01 μg of 277.pCCLsin.PPT.hPGK.GFP.PRE, 0.25 μg of 277.pCCLsin.PPT.hPGK.ERK1‐GFP.PRE or 0.25 μg of 277.pCCLsin.PPT.hPGK.ERK2‐GFP.PRE. After approximately 16 h, cell culture medium was replaced with fresh medium. On the following day, cells were washed three times in PBS and fixed in 4% PFA for 15 min. Cells transfected with T7‐KPNAs were permeabilized for 10 min at RT with 1% Triton in PBS, whereas cells transfected with C‐(K)‐DYK‐IPO7, HA‐RanBP5 and HA‐KPNB1 were permeabilized with 0.1% Triton in PBS for 10 min. After blocking for 1 h at room temperature with blocking buffer provided with Duolink in situ red Mouse/Rabbit kit (Sigma Aldrich, #DUO92101), cells were incubated overnight at 4°C with anti‐GFP antibody (1:500, Thermo Fisher Scientific, #A11122) and the appropriate anti‐tag antibody: anti‐T7 (1:100, Novagen, #69522), anti‐DYKDDDDK flag (1:200, Cell Signaling, #81465) or anti‐HA (12CA5) (Roche, #11583816001) in the antibody reagent provided with the kit.

The proximity ligation assay was carried out following the manufacturer's instructions. Briefly, PLA probes were applied to the coverslips for 1 h at 37°C. After rinsing in Buffer A, coverslips were incubated with ligase solution for 30 min at 37°C and rinsed again with Buffer A. The polymerase solution was applied to the coverslips for 100 min at 37°C. Coverslips were subsequently washed with Buffer B and mounted with the DAPI mounting medium provided with the kit. Images were acquired from six slides per condition using a confocal microscope (Zeiss LSM710) under a 63× magnification. The number of GFP‐positive cells, DAPI‐stained cells, and red dots was counted manually using Fiji‐ImageJ software.

### *In vivo* administration of drugs for histochemical analysis

3‐NP (3‐Nitropropionic acid) (N5636, Sigma) was dissolved in distilled water to a concentration of 50 mg/ml (pH 7.4) and passed through a 0.2‐μm filter and kept at −80°C until use. The 3‐NP solution was administered i.p. once daily at a dose of 50 mg/kg either for 21 consecutive days in the LV‐injected mice or for 7 days in combination with RB5.

1‐methyl‐4‐phenyl‐1,2,3,6‐tetrahydropyridine‐hydrochloride (MPTP‐HCl, Sigma, Italy) was dissolved in saline and administered i.p. once daily at a dose of 20 mg/kg for 4 days. RB5 (20 mg/kg i.p.) was injected daily 30 min before MPTP. Mice were sacrificed 24 h after the last MPTP injection.

Tg2576 mice were administered with RB5 or Scramble (20 mg/kg i.p.) for either 7 days, for IHC analysis, or with a single injection (20 mg/kg i.p.), for behavioral analysis.

### Stereological analysis of TH positive cells in MPTP‐treated mice

Immuno‐labeled sections were visualized under a Leica DM/RBE light microscope and an Olympus BX50 light microscope with Visiopharm Integrator System software (version 4.4.6.9). TH‐positive cells were counted at 200‐μm intervals. The total number of cells (C) for each animal was calculated using the following formula: C = ∑c × (∑A/∑a) × f, where c is the number of cells in each sampling frame, A is the inclusion area for each section, a is the area of the sampling frame for each animal, and f is the frequency of sectioning.

### Human HD IPSCs cultures

The human induced pluripotent stem cell line CS09iHD109‐n1 (herein referred to as Q109N1) was originally generated from a human fibroblast line ND39258 (RRID:CVCL_ZC78) with an expanded *HTT* allele initially containing 109 pure CAG trinucleotides, and the isogenic control lines used were derived by CRISPR gene‐edit to reduce the CAG repeat length to 20 repeats (Mattis *et al*, 2015). IPSCS were cultured on hESC‐qualified Matrigel (Corning) in E8‐Flex medium (Thermo Fisher) and passaged with RelesR (Stem Cell Technologies) according to the manufacturer's instructions. For neural differentiation iPSCS were grown to 70% confluency and then treated with 10 μM Y‐27632 (Tocris) for 1 h before harvesting by dissociation to a single cell suspension using accutase (Sigma). IPSCs were replated onto fresh Matrigel‐coated plates at a split ratio of 1:2, in E8‐Flex medium containing 10 μM Y‐27632. After overnight culture, IPSCs at 80–90% confluency were washed with PBS pH 7.4 (Gibco) and then switched to neural induction medium (Advanced DMEM/F12 (1:1) supplemented with 2 mM GlutamaxTM (Gibco), 2% Neurobrew without vitamin A (Miltenyi), 10 μM SB431542, 1 μM LDN 193189 (both Stem Cell Technologies), and 1.5 μM IWR1 (Tocris)). Medium was changed daily until day 8, when cells were passaged 1:2 by cell dissociation with Accutase for 5 min at 37°C and replated on Matrigel® coated plates in LIA medium containing (Advanced DMEM/F12 (1:1) supplemented with 2 mM GlutamaxTM, 2% Neurobrew without vitamin A, 0.2 μM LDN 193189, 1.5 μM IWR1, and 20 ng/ml Activin A (Peprotech)). At day 16, cells were plated for neuronal differentiation according to (Telezhkin *et al*, 2016). Cells were plated at a density of 100,000/cm^2^ on nitric acid washed coverslips (Thermo Fisher) treated with 0.1 mg/ml poly‐D‐lysine (Sigma) and Matrigel in SCM1 medium (SCM1 contained: Advanced DMEM/F12 (1:1) supplemented with 2 mM GlutamaxTM, 2% Neurobrew (Miltenyi), 10 μM DAPT, 10 μM Forskolin, 300 μM GABA, 3 μM CHIR99021, 2 μM PD 0332991 (all Tocris), 200 μM ascorbic acid (Sigma‐Aldrich), 10 ng/ml BDNF (Peprotech), and with CaCl_2_ adjusted to a final medium concentration of 1.8 mM). Fifty percent of SCM1 medium was changed every 2–3 days. On day 23, medium was changed to SCM2 medium (SCM2 contained: Advanced DMEM/F12 (1:1): Neurobasal A (Gibco) (50:50), adjusted to 1.8 mM CaCl_2_^−^ and supplemented with 2 mM Glutamax, 2% Neurobrew, 3 μM CHIR99021, 2 μM PD 0332991, 200 μM ascorbic acid, 10 ng/ml BDNF). Fifty percent medium changes were made every 2–3 days until day 37.

RB5 or scrambled inactive peptide was applied for 30 min. After rinsing in PBS, cells were fixed in 4% PFA and permeabilized with increasing concentrations of methanol (33, 50, 75, and 95% in PBS, 2 min each at room temperature) followed by 100% methanol for 10 min at −20°C and decreasing concentrations of methanol (95, 75, 50, 33%, 2 min each at room temperature). After rinsing in PBS, coverslips were blocked in PBS with 5% normal goat serum and 0.3% Triton for 1 h at room temperature and incubated overnight at 4°C with the following primary antibodies: anti‐phospho‐p44/42 MAP kinase (Thr202/Tyr204) (1:200, Cell Signaling, #9101) together with anti‐DARPP‐32 (H3) (1:100, Santa Cruz Biotechnology, # sc‐271111) or anti‐CTIP2 (1:500, Abcam, #ab1876). Coverslips were subsequently incubated with AlexaFluor 488 goat anti‐rabbit or anti‐mouse (1:500, Thermo Fisher Scientific), AlexaFluor 555‐conjugated goat anti rabbit or AlexaFluor555 goat anti‐rat (1:200, Thermo Fisher Scientific) in PBS with 5% normal goat serum and 0.3% Triton and counterstained with DAPI.

Images were acquired using a confocal microscope (Zeiss LSM710) under a 20× objective and analyzed with ImageJ software.

### Electrophysiology (hippocampus)

#### Slice preparations

Acute transverse hippocampal slices (400 μm thick) were prepared from adult male Sprague–Dawley rats (p60‐p90) after a lethal dose of isoflurane inhalation in accordance with the Home Office guidelines and as directed by the Home Office Licensing Team at Cardiff University, as previously described (Glebov *et al*, 2015). The hippocampi were dissected in an ice‐cold slicing solution containing (in mM) 110 choline chloride, 25 glucose, 25 NaHCO_3_, 2.5 KCl, 1.25 NaH_2_PO_4_, 0.5 CaCl_2_, 7 MgCl_2_, 11.6 Na ascorbate, and 3.1 Na pyruvate, then mounted on agar and cut using a Microm HM 650 V vibratome (Thermo Scientific). Slices were incubated in artificial cerebrospinal fluid (aCSF) containing (in mM) 119 NaCl, 10 glucose, 26.1 NaHCO_3_, 2.5 KCl, 1, NaH_2_PO_4_, 2.5 CaCl_2_, and 1.3 MgCl_2_ at 36°C for 30 min, then stored at room temperature until use. All solutions were equilibrated with 95% CO_2_ and 5% O_2_ and had an osmolarity of 300–310 mOsm. Slices were then cut between CA3 and CA1 before being transferred to the recording chamber.

#### Patch‐clamp recordings

Whole‐cell patch clamp recordings were made from CA1 pyramidal neurons visualized under differential interference contrast (Olympus BX51 WI) in a submerged recording chamber perfused with aCSF (~2 ml/min) at 35°C supplemented with 50 μM picrotoxin to block GABA_A_ receptors. Patch electrodes (3–5 MΩ) were pulled from borosilicate filamented glass capillaries on a P‐1000 Flaming‐Brown micropipette puller (Sutter Instruments) and filled with intracellular solution (in mM): 117 KMeSO 3, 8 NaCl, 1 MgCl_2_, 10 HEPES, 0.2 EGTA, 4 MgATP, and 0.3 Na2GTP, pH 7.2, 280 mOsm. For experiments using RB peptides, the intracellular solution was supplemented freshly with either RB5 or the control peptide (Scr) at a final concentration of 50 μM and the pH was re‐adjusted with 1 N KOH. Cells were voltage‐clamped at −70 mV. Recordings were made with a Multiclamp 700B amplifier (Molecular Probes), analog filtered at 10 kHz and digitized at 50 kHz using an Axon Digidata 1,550 acquisition system and Clampex software. Recordings were initiated immediately after establishing whole‐cell configuration. Synaptic responses were evoked by delivering 0.1–1 ms square pulses at 0.1 Hz through a bipolar tungsten electrode inserted in *stratum radiatum* (Digitimer Constant Current Stimulus Isolation unit). Consecutive EPSCs were averaged offline every minute, and the amplitude was normalized to the average of the first minute of recording. Series resistance (Rs) was monitored throughout, and cells with Rs > 30 MΩ or with over 20% change in Rs were discarded.

### Electrophysiology (striatum)

#### Slice preparation

Brain slices containing both the striatum and the cortex were prepared as described (Trusel *et al*, 2015). Mice were anesthetized by isoflurane and decapitated, and their brains were rapidly transferred to ice‐cold dissecting aCSF containing (in mM): 110 Choline‐Cl, 2.5 KCl, 1.25 NaH_2_PO_4_, 7 MgCl_2_6H_2_O, 0.5 CaCl_2_, 25 NaHCO_3_, 25 D‐glucose, 11.6 ascorbic acid, saturated with 95% O_2_ and 5% CO_2_. Horizontal corticostriatal slices (270 μm thick; patch‐clamp recordings) were cut in the dissecting aCSF using a Vibratome 1000S slicer (Leica, Italy), then transferred to normal aCSF containing (in mM): 115 NaCl, 3.5 KCl, 1.2 NaH_2_PO_4_, 1.3 MgCl_2_6H_2_O, 2 CaCl_2_, 25 NaHCO_3_, and 25 D‐glucose and aerated with 95% O_2_ and 5% CO_2_. Following 20 min of incubation at 32°C, slices were kept at RT. During experiments, slices were continuously perfused with aCFS at a rate of 2 ml/min at 28°C.

#### Identification of dSPNs and iSPNs

To identify SPNs of the direct (dSPN) and indirect (iSPN) pathways, neurons were filled with Neurobiotin (0.5 mg/ml, DBA Italia, Segrate, Italy) during recordings and subsequently processed for immunostaining of the A2A receptor (marker of iSPNs) and substance P (marker of dSPNs; Trusel *et al*, 2015). After recording, slices were fixed with 4% PFA in 0.1 M PB (pH 7.4) overnight at 4°C and then incubated with primary antibodies. Rabbit polyclonal antibody to A2A (1:250, Enzo Life Sciences, Farmingdale, New York) and rat monoclonal antibody to substance P (1:200, Millipore, Billerica, Massachusetts) were diluted in 0.1 M PB containing 0.3% Triton X‐100. Sections were subsequently incubated with Alexa 647‐ or Alexa 488‐conjugated secondary antibodies (1:200) and Alexa 568‐conjugated streptavidin (1:1,000) (Invitrogen, Carlsbad, California), mounted on glass slides, and coverslipped. Images were acquired with an inverted Leica TCS SP5 AOBS TANDEM confocal microscope.

#### Patch‐clamp recordings

Whole‐cell recordings were made under direct IR‐DIC (infrared‐differential interference contrast) visualization of neurons in the dorsolateral striatum. Current clamp experiments were performed by using borosilicate patch pipettes (4–6 MW) filled with a potassium‐methyl sulfate‐based internal solution containing (in mM): 135 KMeSO_4_, 10 KCl, 10 HEPES, 1 MgCl_2_, 2 Na_2_‐ATP, and 0.4 Na_3_‐GTP (pH 7.2–7.3, 280–290 mOsm/kg), completed at the beginning of each experimental day with the RB5 peptide or its scrambled‐peptide control, which were diluted at the final concentration of 50 μM. Since, each peptide was dissolved in acetic acid to have a 2.1 mM stock, the pH of the potassium‐methyl sulfate‐based internal solution was adjusted with NaOH every time after adding peptides.

SPNs were clamped at a holding membrane potential of −80 mV. Excitatory postsynaptic potentials (EPSPs) were evoked in the presence of the GABA_A_ receptor antagonist gabazine (10 μM) by cortical stimulation from the somatosensory cortex layer 5 by using a concentric bipolar electrode (80–200 μs, 0.9–1.6 mA, CBAPB75, FHC, Bowdoin, ME) connected to a constant‐current isolation unit (Digitimer LTD, Model DS3) and acquired every 10 s. During HFS‐plasticity induction (4 × 1 s long 100‐Hz trains, repeated every 10 s), the postsynaptic cell was depolarized from −80 to −50 mV. Signals were sampled at 20 kHz filtered to 10 kHz. During the experiments, only cells with a stable resting membrane potential ≤ −78 mV were included in the analysis. Series resistance (range 15–25 MW) was monitored at regular intervals throughout the recording and presented minimal variations (≤20%) in the analyzed cells. The data are reported without corrections for liquid junction potentials. Data were acquired using a Multiclamp 700B amplifier controlled by pClamp 10 software (Molecular Device), and a Digidata 1322 (Molecular Device).

#### Data analysis

The occurrence and magnitude of synaptic plasticity was evaluated by comparing the normalized EPSP amplitudes from the last 5 min of baseline recordings with the corresponding values at 20–30 min after HFS (HFS‐LTD). LTD plots were generated by averaging the peak amplitude of individual EPSPs in 1‐min bins.

### Novel object recognition test (NOR)

NOR was performed as previously described (d'Isa *et al*, 2014). Briefly, the test was performed in an open square box placed in a quiet room with dim light. The objects used were parallelepipeds in metal and glass vials filled with water. The protocol required 3 days and it was performed as follows.

Day 1: mice were individually placed in the empty arena for 5 min to familiarize with it and to measure their anxiety (thigmotaxis trial). The percentage of thigmotaxis is calculated as the time spent in the peripheral zone out of the total time spent in the arena (300 s.). Animals showing a thigmotaxis > 90% are discarded from the sample because considered biased for anxiety. Day 2: mice were placed into the arena for 10 min, where they were allowed to explore two identical objects (training trial). The amount of time spent by mice exploring each object was scored. Day 3: one of the two identical objects (parallelepiped) was changed with a new object (glass vial) and the mice were allowed to explore them for 10 min (test trial). Times of exploration for the familiar object and for the novel object were recorded. The discrimination index (D.I.), defined as the difference between the exploration time for the novel object and the one for the familiar object, divided by total exploration time, was calculated. The sessions were recorded with the video tracking software SMART (Panlab, Barcelona, Spain).

RB5 or Scramble peptides (20 mg/kg, i.p.) were administered 1 h before the training session on day 2 and then long‐term memory was tested in independent groups at different time points: 24, 48, 72, 120, and 168 h post injection.

### Contextual fear conditioning test (CFC)

Lister Hooded male rats (280–350 g) were housed in pairs, in holding rooms maintained at 21°C on a reversed‐light cycle (12 h light/dark; lights on at 10:00 P.M.). All experiments were conducted in the dark period of the rats. RB5 and Scramble peptides were infused into dorsal hippocampus.

#### Surgery and microinfusions into the dorsal hippocampus

Steel double guide cannulae aimed at the dorsal hippocampus (AP ‐3.50, relative to bregma) were surgical implanted under anesthesia at least 1 week prior to behavioral training and microinfusions. Bilateral infusions with either 2 mg/ml Scramble or RB5, 20 min prior to conditioning (pH 7.0, 1.0 μl/side, rate = 0.5 ml/min) via the chronically indwelling cannula were carried out in awake rats using a syringe pump, connected to injectors (28 gauge, projecting 1 mm beyond the guide cannulae) by polyethylene tubing.

#### CFC rat protocol

Conditioning was performed in one of two distinct contexts. These contexts were designed to differ in a number of distinctive characteristics including size, spatial location, odor, and lighting. During the 3 min conditioning training trial, rats received a single scrambled footshock (0.5 mA for 2 s) 2 min after being placed into one of the conditioning contexts. All rats were returned to the home cages after conditioning. Retrieval tests 3 h (postretrieval short‐term memory, STM), or 2 days (long‐term memory, LTM) after recall again consisted of exposing the rat to the conditioning context for 2 min not delivering a foot shock.

#### CFC mice protocol

7‐month‐old Tg2576 mice and WT mice were treated with either Scramble or RB5 (20 mg/kg, i.p.) 1 h before CFC. Mice were individually placed in the conditioning chamber for 120 s of free exploration followed by five foot‐shocks (0.7 mA, 2‐s duration, separated by 60‐s intervals) delivered through the grid floor. Context Fear Memory was assessed 24 h later by returning mice for 5 min to the conditioning chamber and not delivering a foot shock.

For all protocols, freezing behavior served as a measure of conditioned fear to the context during the conditioning and retrieval tests.

### LV injections in Hdh^Q111/+^ mice

Two‐month old Hdh^Q111/+^ knock‐in mice were deeply anesthetized with Isoflurane (Piramal Critical Care) and secured on a stereotaxic frame (Stoelting). Two bilateral LV injections (1 μl each) were performed made into the dorsal striatum at the following coordinates: site 1: AP +1.2, L −1.4, DV −3, site 2: AP +0.3, L −2.3, DV −2.4, site 3: AP +1.2, L +1.4, DV −3, site 4: AP +0.3, L +2.3, and DV −2.4. Sixteen months after injections, mice were subjected to 9‐hole operant testing.

### 9‐hole operant boxes test

Operant testing was conducted in 16 9‐hole operant boxes as previously described (Yhnell *et al*, 2016a). Each operant box contains a horizontal array of nine holes with infrared beams localized to the front of each hole to detect nose pokes. A peristaltic pump delivers liquid reinforcement (strawberry milk) into a magazine at the front of the box.

A week before starting the training, mice underwent water restriction for 18 h/day and were kept under this regimen throughout the experimental procedures. Mice were taught to nose poke on a simple fixed ratio (FR1) schedule of reinforcement: to obtain reward, mice were required to respond to a stimulus light in the central hole via a single nose poke. zQ175 mice were trained daily on this program for 20‐min sessions for 10 days (training phase). Once trained, animals were subdivided into four groups and injected with RB5 or scramble (20 mg/kg, i.p.) peptides 1 h before being tested on the FR1 schedule for the other 9 days. At the end of the behavioral tests, mice were perfused with 4% PFA, and brains were processed for IHC or IF.

### Statistical analysis

Statistical analysis was performed with GraphPad Prism. Data following a normal distribution were analyzed by *t* test or ANOVAs. Nonparametric tests were applied when the data did not follow a normal distribution. Posthoc analyses were only performed for ANOVAs that yielded significant main effects. A full statistical analysis is shown in Appendix Table S1.

Cell cultures and mice were randomly assigned to the treatments. Investigators were blinded to the treatments and genotypes during data acquisition and analysis.

## Author contributions

**Riccardo Brambilla:** Conceptualization; funding acquisition; writing – original draft; writing – review and editing. **Marzia Indrigo:** Data curation. **Ilaria Morella:** Conceptualization; data curation; formal analysis; writing – original draft; writing – review and editing. **Raffaele d'Isa:** Conceptualization; data curation; formal analysis; writing – review and editing. **Alessandro Papale:** Conceptualization; data curation; formal analysis; writing – review and editing. **Riccardo Parra:** Data curation; formal analysis. **Antonia Gurgone:** Data curation; formal analysis. **Daniela Lecca:** Data curation; formal analysis. **Anna Cavaccini:** Data curation; formal analysis. **Cezar M Tigaret:** Conceptualization; data curation; formal analysis; writing – review and editing. **Alfredo Cagnotto:** Conceptualization; data curation. **Kimberley Jones:** Data curation. **Simon Brooks:** Conceptualization; data curation; formal analysis; writing – review and editing. **Gian Michele Ratto:** Conceptualization; data curation; formal analysis; writing – review and editing. **Nicholas D Allen:** Formal analysis; writing – review and editing. **Silvia Middei:** Conceptualization; data curation; formal analysis; writing – review and editing. **Maurizio Giustetto:** Conceptualization; data curation; formal analysis; writing – review and editing. **Anna R Carta:** Data curation; formal analysis; writing – original draft; writing – review and editing. **Raffaella Tonini:** Conceptualization; data curation; formal analysis. **Mario Salmona:** Data curation; formal analysis; writing – review and editing. **Jeremy Hall:** Funding acquisition; writing – review and editing. **Kerrie Thomas:** Conceptualization; data curation; formal analysis; writing – review and editing. **Stefania Fasano:** Conceptualization; data curation; formal analysis; funding acquisition; writing – original draft; writing – review and editing. **Mariah J Lelos:** Data curation; writing – review and editing. **Daniel Orellana:** Data curation; formal analysis.

## Disclosure and competing interests statement

MI, AP, RB, and SF declare a patent application for RB5 (WO/2019/102201).

## Supporting information



Appendix S1

Expanded View Figures PDF

Source Data for Expanded View

PDF+

Source Data for Figure 1

Source Data for Figure 2

Source Data for Figure 3

Source Data for Figure 4

Source Data for Figure 5

Source Data for Figure 6

Source Data for Figure 7

## Data Availability

Raw data is available in a separate file. This study includes no data deposited in external repositories.
