# Supplementary material for: Nuclear ERK1/2 signaling potentiation enhances neuroprotection and cognition via Importinα1/KPNA2
Source: EMBO Mol Med. 2023 Oct 4;15(11):e15984. doi: 10.15252/emmm.202215984 (PMC10630888; doi:10.15252/emmm.202215984)
Supplement: Supplementary file 1 — Appendix S1 [file EMMM-15-e15984-s006.pdf]

Table of content of the *Appendix*

- **Appendix Table S1**

Appendix Table S1: statistical tests with F, p and t values are indicated for each figure, with the number of biological replicates

Figure	Test/post-hoc	Test's results	Post-hoc comparisons	Number of replicates
1A: Day 3	One-way ANOVA, Bonferroni's post-hoc	$F_{4,80}$=178.493 P<0.0001	shRNA ctr vs. shRNA ERK1, P<0.0001<br><br>shRNA ctr vs. shRNAmir-ERK1, P<0.0001<br><br>shRNA ctr vs. shRNA ERK2, P<0.0001<br><br>shRNA ctr vs. shRNAmir-ERK2, P<0.0001	shRNA ctr, n=17<br><br>shRNA ERK1, n=18<br><br>shRNAmir-ERK1, n=18<br><br>shRNA ERK2, n=14<br><br>shRNAmir-ERK2, n=18
1A: Day 5	One-way ANOVA, Bonferroni's post-hoc	$F_{4,107}$=281.083 P<0.0001	shRNA ctr vs. shRNA ERK1, P<0.0001<br><br>shRNA ctr vs. shRNAmir-ERK1, P<0.0001<br><br>shRNA ctr vs. shRNA ERK2, P<0.05<br><br>shRNA ctr vs. shRNAmir-ERK2, P<0.0001.	shRNA ctr, n=25<br><br>shRNA ERK1, n=27<br><br>shRNAmir-ERK1, n=25<br><br>shRNA ERK2, n=17<br><br>shRNAmir-ERK2, n=18
1B: Day 3	One-way ANOVA, Bonferroni's post-hoc	$F_{4,45}$=80.188 P<0.0001	eGFP vs. ERK1, P<0.0001<br><br>eGFP vs. ERK2>1, P<0.0001	n=10 per group
1B: Day 5	One-way ANOVA, Bonferroni's post-hoc	$F_{4,50}$=53.009 P<0.0001	eGFP vs. ERK1, P<0.0001<br><br>eGFP vs. ERK2>1, P<0.0001	eGFP, n=12<br><br>ERK1, n=11<br><br>ERK2>1, n=9<br><br>ERK2, n=12<br><br>ERK1>2, n=12
1C: Day 5	One-way ANOVA, Bonferroni's post-hoc	$F_{2,15}$=29.551 P<0.0001	shRNA ctr vs. shRNA<br><br>ERK2, P<0.0001	n=6 per group
1C: Day 7	One-way ANOVA, Bonferroni's post-hoc	$F_{2,15}$=77.076 P<0.0001	shRNA ctr vs. shRNA ERK2, P<0.0001<br><br>shRNA ctr vs. shRNA ERK1, P<0.01	n=6 per group
1D	One-way ANOVA, Bonferroni's post-hoc	$F_{4,15}$=28.104 P<0.0001	eGFP vs. ERK1, P<0.0001<br><br>eGFP vs. ERK2>1, P<0.0001	n=4 per group

1E: shRNA	One-way ANOVA, Bonferroni's post-hoc	$F_{2,15}$=314.541 P<0.0001	shRNA ctr vs. shRNA ERK2, P<0.0001<br><br>shRNA ctr vs. shRNA ERK1, P<0.05	n=6 per group
1E: shRNAmir	One-way ANOVA, Bonferroni's post-hoc	$F_{2,15}$=57.596 P<0.0001	shRNAmir-ctr vs. shRNAmir-ERK2, P<0.0001<br><br>shRNAmir-ctr vs. shRNAmir-ERK1, P<0.05	n=6 per group
EV1A: ERK2 levels	One-way ANOVA, Bonferroni's post-hoc	$F_{2,12}$=13.76 P<0.001	CTR vs. shERK2, P<0.01<br><br>shERK1 vs. shERK2, P<0.01	n=5 per group
EV1A: ERK1 levels	One-way ANOVA, Bonferroni's post-hoc	$F_{2,12}$=38.01 P<0.0001	CTR vs. shERK1, P<0.0001<br><br>CTR vs. shERK2, P=0.5694<br><br>shERK1 vs. shERK2, P<0.0001	n=5 per group
EV1B	Repeated measures Two-way ANOVA, Bonferroni's post-hoc	time x virus interaction: $F_{8.497, 276.163}$=10.911 P<0.0001	shRNA ctr vs. shRNA ERK2, each day, P<0.0001<br><br>shRNA ctr vs. shRNA ERK1, day 5-6, P<0.05<br><br>shRNA ctr vs. shRNA ERK1, day 7-8, P<0.01<br><br>shRNA ctr vs. shRNA ERK1, day 9-10,P<0.0001	shRNA ctr, n=26<br><br>shRNA ERK2, n=13<br><br>shRNA ERK1, n=29
EV1C	Repeated measures Two-way ANOVA, Bonferroni's post-hoc	time x virus interaction: $F_{10,190}$=8.362 P<0.0001	ctr vs. ERK1, each day, P<0.0001<br><br>ctr vs. ERK2>1, day 1-2, P<0.01<br><br>ctr vs. ERK2>1, day 3-10, P<0.0001	ctr, n=14<br><br>ERK1, n=13<br><br>ERK2>1, n=14

EV1D	Repeated measures Two-way ANOVA, Bonferroni's post-hoc	time x virus interaction $F_{10,190}$=2.832 P<0.01	ctr vs. ERK1>2, day 1-2, P<0.05<br><br>ctr vs. ERK1>2, day 3-4, P<0.01<br><br>ctr vs. ERK1>2, day 5-6, P<0.05<br><br>ctr vs. ERK1>2, day 7-8, P<0.01<br><br>ctr vs. ERK1>2, day 9-10, P<0.0001<br><br>ctr vs. ERK2, day 1-2, P<0.01<br><br>ctr vs. ERK2, day 3-4, P<0.0001<br><br>ctr vs. ERK2, day 5-6 P<0.01<br><br>ctr vs. ERK2, day 7-10, P<0.0001	ctr, n=14<br><br>ERK2, n=14<br><br>ERK1>2, n=14
EV1E	One-way ANOVA, Games-Howell's post hoc	Welch's $F_{2,12.411}$=491.540 P<0.0001	shRNA ctr vs. shRNA ERK1, P<0.0001<br><br>shRNA ctr vs. shRNA ERK2, P<0.0001	n=10 (days) per group
EV1F	One-way ANOVA, Bonferroni's post-hoc	$F_{2,24}$=312.600 P<0.0001	ctr vs. ERK2>1, P<0.0001<br><br>ctr vs. ERK1, P<0.0001	n=10 (days) per group
EV1G	One-way ANOVA, Bonferroni's post-hoc	$F_{2,24}$=181.727 P<0.0001	ctr vs. ERK1>2, P<0.0001<br><br>ctr vs. ERK2, P<0.0001	n=10 (days) per group
EV1I	Kolmogorov-Smirnov Test	Control vs. shRNA ERK1, P<0.001<br><br>Control vs. ERK1, P<0.001<br><br>Control vs. ERK2, P=0.4<br><br>Control vs. ERK1>2, P<0.001<br><br>Control vs. ERK2>1, P<0.01		Control, n=9 (mice), 105 (spines)<br><br>shRNA ERK1, n=3,56<br><br>ERK1, n=3,51<br><br>ERK2, n=3,46<br><br>ERK1>2, n=3,47<br><br>ERK2>1, n=3,40
2B	One-way ANOVA, Bonferroni's post-hoc	$F_{2,19}$=13.96 P<0.001	CTR vs. RB5, P<0.01<br><br>CTR vs. FBS, P<0.001<br><br>RB5 vs. FBS p=0.1579	CTR, n=8<br><br>RB5, n=8<br><br>FBS, n=6

2C	Kruskal-Wallis test,<br>Dunn's post-hoc	H(2)=19.96<br>P<0.0001	CTR vs. RB5, P<0.05<br><br>CTR vs. FBS, P<0.0001<br><br>RB5 vs. FBS P=0.1579	CTR, n=9<br><br>RB5, n=9<br><br>FBS, n=6
2D	Two-way ANOVA, Bonferroni's post-hoc	peptide x treatment interaction:<br>$F_{1,59}$=73.314<br>P<0.0001<br>effect of glutamate:<br>$F_{1,59}$=119.285<br>P<0.0001	SCR GLU vs. RB5 GLU, P<0.0001	SCR Veh, n=13<br><br>SCR GLU, n=13<br><br>RB5 Veh, n=18<br><br>RB5 GLU, n=19
2E	Two-way ANOVA, Bonferroni's post-hoc	peptide x treatment interaction:<br>$F_{1,45}$=40.970<br>P<0.0001<br>effect of glutamate:<br>$F_{1,45}$=61.369<br>P<0.0001	SCR Veh vs. SCR GLU, P<0.0001<br><br>RB5 Veh vs. RB5 GLU, P=1.000	SCR Veh, n=9<br><br>SCR GLU, n=12<br><br>RB5 Veh, n=14<br><br>RB5 GLU, n=14
2F	Two-way ANOVA, Bonferroni's post-hoc	genotype x treatment interaction:<br>$F_{1,41}$=5.448<br>P<0.05<br>effect of glutamate:<br>$F_{1,41}$=9.617<br>P<0.01	ERK1-/- GLU vs. ERK1+/+ GLU, P<0.01	ERK1+/+ Veh, n=11<br><br>ERK1+/+ GLU, n=11<br><br>ERK1-/- Veh, n=12<br><br>ERK1-/- GLU, n=11
2G	Two-way ANOVA, Bonferroni's post-hoc	genotype x treatment interaction:<br>$F_{1,43}$=9.900<br>P<0.01<br>effect of glutamate:<br>$F_{1,43}$=11.315<br>P<0.01	ERK1+/+ GLU vs ERK1-/- GLU, P<0.01<br><br>ERK1-/- Veh vs. ERK1-/- GLU, P=0.701	ERK1+/+ Veh, n=14<br><br>ERK1+/+ GLU, n=15<br><br>ERK1-/- Veh, n=9<br><br>ERK1-/- GLU, n=8
EV2A: pERK1 levels	Kruskal-Wallis test,<br>Dunn's post-hoc	H(2)= 25<br>P<0.0001	CTR vs. RB5, P<0.01<br><br>CTR vs. FBS, P<0.0001<br><br>RB5 vs. FBS, P=0.2147	n=12 per group
EV2A: pERK2 levels	Kruskal-Wallis test,<br>Dunn's post-hoc	H(2)=26.8<br>P<0.0001	CTR vs. RB5, P<0.01<br><br>CTR vs. FBS, P<0.0001<br><br>RB5 vs. FBS, P=0.1511	n=12 per group
EV2B: ERK1 levels	Kruskal-Wallis test	H(2)=3.893<br>P=0.1427		n=12 per group
EV2B: ERK2 levels	Kruskal-Wallis test	H(2)= 3.299<br>P=0.1922		n=12 per group
EV2C: p-p54 levels	Kruskal-Wallis test,<br>Dunn's post-hoc	H(2)= 24.61<br>P<0.0001	CTR vs. RB5, P=0.7834<br><br>CTR vs. AN, P<0.0001<br><br>RB5 vs. AN, P<0.001	n=12 per group

EV2C: p-p46 levels	Kruskal-Wallis test,<br>Dunn's post-hoc	H(2)= 24.15<br>P<0.0001	CTR vs. RB5, P>0.9999<br><br>CTR vs. AN, P<0.0001<br><br>RB5 vs. AN, P<0.001	n=12 per group
EV2D: p54 levels	Kruskal-Wallis test	H(2)=1.155<br>P=0.5614		n=12 per group
EV2D: p46 levels	One-way ANOVA	$F_{2,33}$=1.797<br>P=0.1817		n=12 per group
3A	One-way ANOVA, Bonferroni's post-hoc	$F_{5,24}$=10.942<br>P<0.0001	SCR vs. RB5 1h, P<0.0001<br><br>SCR vs. RB5 6h, P<0.0001<br><br>SCR vs. RB5 3h, P<0.001	n=5 per group
3B	One-way ANOVA, Bonferroni's post-hoc	$F_{4,18}$=17.469<br>P<0.0001	SCR vs. RB5 1h, P<0.01<br><br>SCR vs. RB5 3h, P<0.0001<br><br>SCR vs. RB5 6h, P<0.05	SCR, n=3<br><br>RB5 1h, n=5<br><br>RB5 3h, n=5<br><br>RB5 6h, n=5<br><br>RB5 12h, n=5
3C	One-way ANOVA, Bonferroni's post-hoc	$F_{5,24}$=14.292<br>P<0.0001	SCR vs. RB5 10mg/kg, P<0.0001<br><br>SCR vs. RB5 20mg/kg, P<0.0001	n=5 per group
3D	Independent sample t-test	t_{28}= -5.340<br>P<0.0001		n=15 per group
3E	Independent sample t-test	t_{29}= -2.808<br>P<0.01		SCR, n=16<br><br>RB5, n=15
3F	Independent sample t-test	t_{28}= -5.404<br>P<0.0001		SCR, n=16<br><br>RB5, n=14
3G	Independent sample t-test	t_{29}=-12.106<br>P<0.0001		SCR, n=16<br><br>RB5, n=15
3H	Independent sample t-test	t_{29}=-12.743<br>P<0.0001		SCR, n=16<br><br>RB5, n=15
3I	Independent sample t-test	t_{28}= 0.609<br>P=0.547		n=15 per group
3J	Independent sample t-test	t_{28}= 0.618<br>P=0.541		SCR, n=16<br><br>RB5, n=14
3K	Independent sample t-test	t_{29}= 2.191<br>P<0.05		SCR, n=16<br><br>RB5, n=15
3L	Independent sample t-test	t_{28}= 1.878<br>P=0.071		SCR, n=16<br><br>RB5, n=15

3M	Two-way ANOVA, Bonferroni's post-hoc	genotype x peptide interaction: $F_{1,30}$=17.494 P<0.0001<br><br>effect of peptide: $F_{1,30}$=37.505 P<0.0001	ERK1+/+ SCR vs. ERK1+/+ RB5, P<0.0001	ERK1+/+ SCR, n=10<br><br>ERK1+/+ RB5, n=8<br><br>ERK1 -/- SCR, n=12<br><br>ERK1 -/- RB5, n=10
3N	Two-way ANOVA, Bonferroni's post-hoc	genotype x peptide interaction: $F_{1,30}$=17.494 P<0.0001<br><br>effect of peptide: $F_{1,30}$=37.505 P<0.0001	ERK1+/+ SCR vs. ERK1+/+ RB5, P<0.0001	ERK1+/+ SCR, n=8<br><br>ERK1+/+ RB5, n=8<br><br>ERK1-/- SCR, n=9<br><br>ERK1-/- RB5, n=9
3O	Two-way ANOVA	genotype x peptide interaction: $F_{1,31}$=2.601 P=0.117<br><br>effect of peptide: $F_{1,31}$=3.266 P=0.080<br><br>effect of genotype: $F_{1,31}$=1.185 P=0.285		ERK1+/+ SCR, n=9<br><br>ERK1+/+ RB5, n=10<br><br>ERK1-/- SCR, n=8<br><br>ERK1-/- RB5, n=8
4A: IPO7	One-way ANOVA, Games Howell's post-hoc	Welch's $F_{2,6.667}$=12.717 P<0.01	GFP vs. ERK1, P<0.05<br><br>GFP vs. ERK2, P<0.05<br><br>ERK1 vs. ERK2, P=0.98	GFP, n=5<br><br>ERK1, n=6<br><br>ERK2, n=6
4A: KPNB1	One-way ANOVA	$F_{2,16}$=2.644 P=0.106		GFP, n=6<br><br>ERK1, n=5<br><br>ERK2, n=6
4A: RanBP5	Kruskal-Wallis test, Dunn's post-hoc	H(2)=8.430 P <0.05	GFP vs. ERK1, P=0.279<br><br>GFP vs. ERK2, P<0.01<br><br>ERK1 vs. ERK2, P=0.083	n=6 per group
4B: KPNA2	One-way ANOVA, Games Howell's post-hoc	Welch's $F_{2,11.339}$=25.5, P<0.001	GFP vs. ERK1, P<0.0001<br><br>GFP vs. ERK2, P<0.05<br><br>ERK1 vs. ERK2, P<0.01	GFP, n=8<br><br>ERK1, n=9<br><br>ERK2, n=8
4B: KPNA7	One-way ANOVA, Games Howell's post-hoc	Welch's $F_{2,8.735}$=29.603 P<0.001	GFP vs. ERK1, P<0.01<br><br>GFP vs. ERK2, P<0.001<br><br>ERK1 vs. ERK2, P<0.01	n=6 per group

4C: KPNA3	One-way ANOVA, Games Howell's post-hoc	Welch's $F_{2,5.445}$=14.557 P<0.01	GFP vs. ERK1, P=0.208<br><br>GFP vs. ERK2, P<0.05<br><br>ERK1 vs. ERK2, P>0.999	n=5 per group
4C: KPNA4	Kruskal-Wallis test, Dunn's post-hoc	H(2)=9.609 P<0.01	GFP vs. ERK1, P<0.05<br><br>GFP vs. ERK2, P<0.05<br><br>ERK1 vs. ERK2, P=0.607	GFP, n=5<br><br>ERK1, n=6<br><br>ERK2, n=6
4D: KPNA1	One-way ANOVA, Games Howell's post-hoc	Welch's $F_{2,6.186}$=13.558 P<0.01	GFP vs. ERK1, P<0.05<br><br>GFP vs. ERK2, P<0.05<br><br>ERK1 vs. ERK2, P=0.925	GFP, n=5<br><br>ERK1, n=6<br><br>ERK2, n=5
4D: KPNA5	One-way ANOVA, Games Howell's post-hoc	Welch's $F_{2,11.261}$=21.022 P<0.001	GFP vs. ERK1, P<0.01<br><br>GFP vs. ERK2, P<0.01<br><br>ERK1 vs. ERK2, P=0.989	n=9 per group
4D: KPNA6	One-way ANOVA, Bonferroni's post-hoc	$F_{2,17}$=5.088 P<0.05	GFP vs. ERK1, P>0.999<br><br>GFP vs. ERK2, P<0.05<br><br>ERK1 vs. ERK2, P=0.068	n=6 per group
4E: KPNA2	Two-way ANOVA, Bonferroni's post-hoc	transfection x peptide interaction: $F_{2,30}$=67.20 P<0.0001<br><br>effect of transfection: $F_{2,30}$=36.16 P<0.0001<br><br>effect of peptide: $F_{1,30}$=0.601 P=0.441	GFP SCR vs. ERK1 SCR, P<0.0001<br><br>GFP SCR vs. ERK2 SCR, P>0.999<br><br>ERK1 SCR vs. ERK2 SCR, P<0.0001<br><br>GFP SCR vs. GFP RB5, P>0.999<br><br>ERK1 SCR vs. ERK1 RB5, P<0.0001<br><br>ERK2 SCR vs. ERK2 RB5, P<0.0001	n=6 per group

4E: KPNA5	Two-way ANOVA, Bonferroni's post-hoc	transfection x peptide interaction: $F_{2,29}$=0.2903 P=0.7502<br><br>effect of transfection: $F_{2,29}$=39.03 P<0.0001<br><br>effect of treatment: $F_{1,29}$=1.191 P=0.2841	GFP SCR vs. ERK1 SCR, P<0.0001<br><br>GFP SCR vs. ERK2 SCR, P<0.0001<br><br>ERK1 SCR vs. ERK2 SCR, P>0.999<br><br>GFP RB5 vs. ERK1 RB5, P<0.0001<br><br>GFP RB5 vs. ERK2 RB5, P<0.001<br><br>ERK1 RB5 vs. ERK2 RB5, P>0.999<br><br>GFP SCR vs. GFP RB5, P>0.999<br><br>ERK1 SCR vs. ERK1 RB5, P>0.999<br><br>ERK2 SCR vs. ERK2 RB5, P>0.999	GFP SCR, n=6<br><br>GFP RB5, n=6<br><br>ERK1 SCR, n=5<br><br>ERK1 RB5, n=6<br><br>ERK2 SCR, n=6<br><br>ERK2 RB5, n=6
4F: siRNA KPNA2	Independent samples t test	t_4=4.552 P<0.05		n=3 per group
4F: siRNA IPO7	Independent samples t test	t_4=3.109 P<0.05		n=3 per group
4G	Two-way ANOVA, Bonferroni's post-hoc	siRNA x peptide interaction: $F_{2,24}$=30 P<0.0001<br><br>effect of siRNA: $F_{2,24}$=13.41 P<0.001<br><br>effect of peptide: $F_{1,24}$=26.26 P<0.0001	CTR SCR vs. siRNA KPNA2 SCR, P=0.1093<br><br>CTR SCR vs. CTR siRNA IPO7, P>0.999<br><br>CTR siRNA KPNA2 vs. CTR siRNA IPO7, P=0.3976<br><br>CTR RB5 vs. siRNA KPNA2 RB5, P<0.0001<br><br>CTR RB5 vs. siRNA IPO7 RB5, P<0.0001<br><br>siRNA KPNA2 RB5 vs. siRNA IPO7 RB5, P=0.8481<br><br>CTR SCR vs. CTR RB5, P<0.0001<br><br>siRNA KPNA2 SCR vs. siRNA KPNA2 RB5, P=0.4905<br><br>siRNA IPO7 SCR vs. siRNA IPO7 RB5, P=0.7046	n=5 per group

4H	Mann-Whitney test	CTR SCR vs. CTR RB5, $P<0.01$<br><br>siRNA KPNA2 CTR vs. siKPNA2 RB5, $P=0.1143$<br><br>siRNA IPO7 SCR vs siIPO7 RB5, $P=0.4286$<br><br>siRNA KPNA2 SCR vs. CTR SCR, $P<0.01$<br><br>siIPO7 SCR vs. CTR SCR, $P<0.05$<br><br>siKPNA2 RB5 vs. CTR RB5, $P<0.05$<br><br>siRNA IPO7 RB5 vs. CTR RB5, $P=0.0556$<br><br>siRNA IPO7 SCR vs. siRNA KPNA2 SCR, $P<0.01$<br><br>siRNA IPO7 RB5 vs. siRNA KPNA2 RB5, $P<0.05$		CTR SCR, n=5<br><br>CTR RB5, n=5<br><br>siRNA KPNA2 SCR, n=6<br><br>siRNA KPNA2 RB5, n=4<br><br>siRNA IPO7 SCR, n=4<br><br>siRNA IPO7 RB5, n=4
EV3	One-way ANOVA, Bonferroni's post-hoc	$F_{2,254}=36.86$ $P<0.0001$	GFP vs. ERK1, $P<0.0001$<br>GFP vs. ERK2, $P>0.999$<br>ERK1 vs. ERK2, $P<0.0001$	GFP, n=84<br><br>ERK1, n=83<br><br>ERK2, n=90
5A	Two-way ANOVA, Bonferroni's post-hoc	peptide x toxin interaction: $F_{1,33}=13.232$ $P<0.001$<br><br>effect of peptide: $F_{1,33}=44.250$ $P<0.0001$	SCR 3-NP vs. RB5 3-NP, $P<0.0001$	SCR saline, n=9<br><br>RB5 saline, n=9<br><br>SCR 3-NP, n=9<br><br>RB5 3-NP, n=10
5B	Two-way ANOVA, Bonferroni's post-hoc	peptide x toxin interaction: $F_{1,26}=12.700$ $P<0.001$<br><br>effect of peptide: $F_{1,26}=30.556$ $P<0.001$	SCR 3-NP vs. RB5 3-NP, $P<0.0001$	SCR saline, n=7<br><br>RB5 saline, n=7<br><br>SCR 3-NP, n=9<br><br>RB5 3-NP, n=7

5C	Two-way ANOVA, Bonferroni's post-hoc	peptide x genotype interaction: $F_{1,16}$=7.125 P<0.05<br><br>effect of peptide: $F_{1,16}$=31.354 P<0.0001	Hdh$^{Q111/+}$ SCR vs. Hdh $^{Q111/+}$ RB5, P<0.0001	n=5 per group
5D	Two-way ANOVA, Bonferroni's post-hoc	peptide x genotype interaction: $F_{1,29}$=15.851 P<0.0001<br><br>effect of genotype: $F_{1,29}$=18.605 P<0.0001	Hdh$^{Q111/+}$ RB5 vs. Hdh$^{Q111/+}$ SCR, P<0.0001<br><br>WT RB5 vs. Hdh$^{Q111/+}$ RB5, P<0.01<br><br>WT SCR vs. WT RB5, P<0.01	WT SCR, n=7<br><br>WT RB5, n=8<br><br>Hdh$^{Q111/+}$ SCR, n=9<br><br>Hdh$^{Q111/+}$ RB5, n=9
5E	Two-way ANOVA, Bonferroni's post-hoc	genotype x treatment interaction: $F_{1,18}$=1.796 P=0.1969<br><br>effect of genotype: $F_{1,18}$=10.18 P<0.01<br><br>effect of treatment $F_{1,18}$=30.90 P<0.0001	CTR SCR vs. CTR HD, P<0.01<br><br>CTR SCR vs. CTR RB5, P<0.05<br><br>HD SCR vs. HD RB5, P<0.001	CTR SCR, n=6<br><br>CTR RB5, n=5<br><br>HD SCR, n=5<br><br>HD RB5, n=6
5F	Two-way ANOVA, Bonferroni's post-hoc	genotype x treatment interaction: $F_{1,20}$=8.923 P<0.01<br><br>effect of genotype: $F_{1,20}$=5.653 P<0.05<br><br>effect of treatment: $F_{1,20}$=78.66 P<0.0001	CTR SCR vs. CTR HD, P<0.01<br><br>CTR SCR vs. CTR RB5, P<0.01<br><br>HD SCR vs HD RB5, P<0.0001	n=6 per group
5G	One-way ANOVA, Bonferroni's post-hoc	$F_{2,32}$=6.501 P<0.01	SAL vs. SCR MPTP P<0.05<br><br>SAL vs. RB5 MPTP, P>0.999<br><br>SCR MPTP vs. RB5 MPTP, P<0.05	SAL, n=8<br><br>SCR MPTP, n=15<br><br>RB5 MPTP, n=14
5H	Independent samples t test	t_{27}=3.168 P<0.01		SCR MPTP, n=15<br><br>RB5 MPTP, n=14

5I	One-way ANOVA, Bonferroni's post-hoc	$F_{2,34}$=15.54 P<0.0001	SAL vs. SCR MPTP, P<0.05<br><br>SAL vs. RB5 MPTP, P<0.0001<br><br>SCR MPTP vs. RB5 MPTP, P<0.05	SAL, n=8<br><br>SCR MPTP, n=15<br><br>RB5 MPTP, n=14
5J	Two-way ANOVA, Bonferroni's post-hoc	peptide x genotype interaction: $F_{1,23}$=18.245 P<.0001<br><br>effect of peptide: $F_{1,23}$=90.240 P<0.0001	Tg2576 RB5 vs. Tg2576 SCR, P<0.0001<br><br>WT RB5 vs. WT SCR, P<0.01	WT SCR, n=6<br><br>WT RB5, n=7<br><br>Tg2576 SCR, n=6<br><br>Tg2576 RB5, n=8
5K	Two-way ANOVA, Bonferroni's post-hoc	peptide x genotype interaction: $F_{1,23}$=12.099 P<0.05<br><br>effect of peptide: $F_{1,23}$=28.431 P<0.0001	Tg2576 RB5 vs. Tg2576 SCR, P<0.0001	WT SCR, n=7<br><br>WT RB5, n=7<br><br>Tg2576 SCR, n=6<br><br>Tg2576 RB5, n=7
5L	Two-way ANOVA, Bonferroni's post-hoc	effect of peptide: $F1_{,26}$=14.088 P<0.001<br><br>effect of genotype: $F_{1,26}$=21.857 P<0.0001	Tg2576 SCR vs. Tg2576 RB5, P<0.05<br><br>WT SCR vs Tg2576 SCR, P<0.01	WT SCR, n=7<br><br>WT RB5, n=9<br><br>Tg2576 SCR, n=7<br><br>Tg2576 RB5, n=7
EV4A	Two-way ANOVA, Bonferroni's post-hoc	virus x treatment interaction: $F_{1,15}$=4.953 P<0.05<br><br>effect of virus: $F_{1,15}$=21.222 P<.0001	shRNA ctr 3-NP vs. shRNA ERK1 3-NP, P<0.0001	shRNA ctr saline, n=4<br><br>shRNA ERK1 Saline, n=5<br><br>shRNA ctr 3-NP, n=5<br><br>shRNA ERK1 3-NP, n=5
EV4B	Two-way ANOVA, Bonferroni's post-hoc	virus x treatment interaction: $F_{2,33}$=66.320 P<0.0001<br><br>effect of the viruses: $F_{2,33}$=194.003 P<0.0001	ctr 3-NP vs. ERK2 3-NP, P<0.0001<br><br>ctr 3-NP vs. ERK1>2 3-NP, P<0.0001	ctr saline, n=8<br><br>ctr 3-NP, n=10<br><br>ERK2 saline, n=5<br><br>ERK2 3-NP, n=6<br><br>ERK1>2 saline, n=5<br><br>ERK1>2 3-NP, n=5

EV4C	Two-way ANOVA, Bonferroni's post-hoc	genotype x treatment interaction: $F_{1,41}$=20.629 P<0.0001<br><br>effect of genotype: $F_{1,41}$=44.647 P<0.0001	ERK1+/+ 3-NP vs. ERK1-/- 3-NP, P<0.0001	ERK1+/+ saline, n=11<br><br>ERK1-/- saline, n=9<br><br>ERK1+/+ 3-NP, n=13<br><br>ERK1-/- 3-NP, n=12
6A	Independent samples t test, assuming unequal variances	$t_{16,46}$= 3.65 P<0.01		RB5, n=12 cells, 4 mice<br><br>SCR, n=8 cells, 4 mice
6B	SCR and RB5: Repeated measures one-way ANOVA, Tukey's post-hoc<br><br>dSPN_RB5 vs. dSPN_SCR: Mann Whitney test	RB5: $F_{6,44}$=0.8, P=0.45<br><br>SCR: $F_{4,44}$=4.2 P<0.05	SCR: P<0.05<br><br>dSPN_RB5 vs. dSPN_SCR: P<0.01	RB5, n=7 cells<br>SCR, n=5 cells
6C: 24 h	Independent sample t-test	t_{26}= 3.32 P<0.01		SCR, n=14<br><br>RB5, n=14
6C: 48 h	Independent sample t-test	t_{38}= 4.210 P<0.001		SCR, n=18<br><br>RB5, n=22
6C: 48 h	One sample t-test	SCR: t_{17}= 0.566 P=0.579<br><br>RB5: t_{21}= 8.077 P<0.0001		SCR, n=18<br><br>RB5, n=22
6C: 72 h	Independent sample t-test	t_{14}= 7.899 P<0.0001		SCR, n=7<br><br>RB5, n=9
6C: 72 h	One sample t-test	RB5: t_6= 12.341 P<0.0001<br><br>SCR: t_6= 9.073 P<0.0001		SCR, n=7<br><br>RB5, n=9
6C: 120 h	Independent sample t-test	t_{23}= 4.290 P<0.001		SCR, n=10<br><br>RB5, n=15
6C: 120 h	One sample t-test	RB5: t_{14}= 9.143 P<0.0001<br><br>SCR: t_9= 2.230 P=0.053		SCR, n=10<br><br>RB5, n=15
6C: 168 h	Independent sample t-test	t_{18}= 0.777 P=0.447		SCR, n=10<br><br>RB5, n=10

6D	Repeated Measures Two-way ANOVA, post-hoc t tests	test x group interaction; $F_{3,27}$=9.439 P<0.001<br><br>group effect: $F_{1,9}$=23.171 P<0.001	PostUS: P<0.01<br><br>STM: P<0.01<br><br>LTM: P<0.05	SCR, n=6<br><br>RB5, n=5
6E	Two-way ANOVA, Bonferroni's post-hoc	genotype x treatment interaction: $F_{1,26}$= 19.583 P<0.0001	Tg2576 SCR vs. Tg2576 RB5, P<0.0001	WT SCR, n=7<br><br>Tg2576 SCR, n=7<br><br>WT RB5, n=8<br><br>Tg2576 RB5, n=8
6F-top panel	Repeated Measures Three-way ANOVA, Bonferroni's post hoc	time effect: $F_{2.367,61.550}$=8.054 P<0.0001<br><br>genotype effect: $F_{1,26}$=90.156 P<0.0001<br><br>treatment effect: $F_{1,26}$=45.390 P<0.0001	zQ175 RB5 vs. zQ175 SCR: day 11-12, P<0.05<br>day 13-17, P<0.0001<br>day 18-19, P<0.01<br><br>zQ175 SCR vs. WT SCR: day 11-19, P<0.0001<br><br>WT SCR vs. WT RB5:<br>day 11-16, P<0.01<br>day 17, P=0.077<br>day 18-19, P<0.01	zQ175 RB5, n=7<br><br>zQ175 SCR, n=8<br><br>WT RB5, n=7<br><br>WT SCR, n=8
6F-bottom panel	Two-way ANOVA, Bonferroni's post-hoc	group effect (training phase): $F_{1,36}$=0.1127 P=0.739<br><br>genotype effect (training phase): $F_{1,36}$=14.54 P<0.001<br><br>group x genotype interaction (training phase): $F_{1,36}$=0.014 P=0.9059<br><br>group effect (treatment phase): $F_{1,32}$=208.9 P<0.0001<br><br>genotype effect (treatment phase): $F_{1,32}$=710.1 P<0.0001<br><br>group x genotype interaction (treatment phase): $F_{1,32}$=2.91 P=0.097	WT SCR vs. zQ175 SCR, P<0.05 (training phase)<br><br>WT RB5 vs. zQ175 RB5, P<0.05 (training phase)<br><br>WT SCR vs. zQ175 SCR P<0.0001 (treatment phase)<br><br>WT RB5 vs. zQ175 RB5 P<0.0001 (treatment phase)<br><br>WT SCR vs. WT RB5 P<0.0001 (treatment phase)<br><br>zQ175 SCR vs. zQ175 RB5 P<0.0001 (treatment phase)	n=10 (days) per group (training phase)<br><br>n=9 (days) per group (treatment phase)

6G-top panel	Repeated Measures Three-way ANOVA, Bonferroni's post hoc	time effect: $F_{3.309,105.898}$=71.004 P<0.0001<br><br>genotype effect: $F_{1,32}$=22.684 P<0.0001<br><br>treatment effect: $F_{1,32}$=6.658 P<0.05	$Hdh^{Q111/+}$ GFP vs. $Hdh^{Q111/+}$ ERK2 OE:<br>day 12-16, P<0.05<br>day 17-18, P<0.01<br>day 19, P<0.05<br>day 20-25. P<0.01<br>day 26 P<0.0001<br><br>$Hdh^{Q111/+}$ GFP vs. WT GFP:<br>day 8-11, P<0.01<br>day 12-26, P<0.0001	WT GFP, n=10<br><br>WT ERK2 OE, n=9<br><br>$Hdh^{Q111/+}$ GFP, n=9<br><br>$Hdh^{Q111/+}$ERK2 OE, n=8
6G-bottom panel	Two-way ANOVA, Bonferroni's post-hoc	treatment effect: $F_{1,100}$=6.48 P<0.05<br><br>genotype effect: $F_{1,100}$=63.06 P<0.0001<br><br>genotype x treatment interaction: $F_{1,100}$=17.25 P<0.0001	WT GFP vs $Hdh^{Q111/+}$ GFP, P<0.0001<br><br>WT ERK2 OE vs $Hdh^{Q111/+}$ ERK2 OE, P<0.05<br><br>WT GFP vs WT ERK2 OE, P=0.5163<br><br>$Hdh^{Q111/+}$ GFP vs $Hdh^{Q111/+}$ ERK2OE, P<0.0001	n=26 (days) per group
EV5-top panel	Repeated Measures Three-way ANOVA	genotype effect: $F_{1,31}$=22.372 P<0.0001<br><br>treatment effect: $F_{1,31}$=0.004 P=0.950		WT SCR, n=8<br><br>WT RB5, n=8<br><br>zQ175 SCR, n=10<br><br>zQ175 RB5, n=9

EV5-bottom panel	Two-way ANOVA, Bonferroni's post-hoc	group effect (training phase): $F_{1,36}$=0.032 P=0.857<br><br>genotype effect (training phase): $F_{1,36}$=16.86 P<0.001<br><br>Group x genotype effect interaction (training phase): $F_{1,36}$=0.005, P=0.943<br><br>group effect (treatment phase): $F_{1,32}$=2.386, P=0.132<br><br>genotype effect (treatment phase): $F_{1,32}$=4101 P<0.0001<br><br>group x genotype interaction (treatment phase): $F_{1,32}$=10.28 P<0.01	WT SCR vs. zQ175 SCR (training phase), P<0.05<br><br>WT RB5 vs. zQ175 RB5 (training phase), P<0.05<br><br>WT SCR vs. zQ175 SCR (treatment phase), P<0.0001<br><br>WT RB5 vs. zQ175 RB5 (treatment phase), P<0.0001<br><br>WT SCR vs. WT RB5 (treatment phase),P<0.01<br><br>zQ175 SCR vs. zQ175 RB5 (treatment phase), P=0.497	n=10 (days) per group (training phase)<br><br>n=9 (days) per group (treatment phase)
7A	Two-way ANOVA, Fisher's LSD post-hoc	genotype x treatment interaction: $F_{1,26}$=2.042 P=0.1649<br><br>treatment effect: $F_{1,26}$=138.7 P<0.0001<br><br>genotype effect: $F_{1,26}$=20.19 P<0.001	WT SCR vs. zQ175 SCR, P<0.05<br><br>WT RB5 vs. zQ175 RB5, P<0.001<br><br>WT SCR vs. WT RB5 P<0.0001<br><br>zQ175 SCR vs. zQ175 RB5, P<0.0001	WT SCR, n=8<br><br>WT RB5, n=7<br><br>zQ175 SCR, n=8,<br><br>zQ175 RB5 n=7
7B	Two-way ANOVA	genotype x treatment interaction: $F_{1,26}$=0.9124 P=0.3483<br><br>treatment effect: $F_{1,26}$=0.0122 P=0.9129<br><br>genotype effect: $F_{1,26}$=0.0085, P=0.9270		WT SCR, n=8<br><br>WT RB5, n=7<br><br>zQ175 SCR, n=8,<br><br>zQ175 RB5 n=7

7C	Two-way ANOVA, Fisher's LSD post-hoc	genotype x treatment interaction: $F_{1,26}$=9.093 P<0.01<br><br>treatment effect: $F_{1,26}$=5.911 P<0.05<br><br>genotype effect: $F_{1,26}$=6.934 P<0.05	WT SCR vs. WT RB5, P=0.7891<br><br>zQ175 WT vs. zQ175 RB5 P<0.001<br><br>WT SCR vs. zQ175 SCR, P<0.001<br><br>WT RB5 vs zQ175 RB5 P=0.6925	WT SCR, n=8<br><br>WT RB5, n=7<br><br>zQ175 SCR, n=8,<br><br>zQ175 RB5 n=7
7D	Independent samples t test	t_{13}=0.069 P=0.9458		zQ175 SCR, n=8,<br><br>zQ175 RB5 n=7
7E	Two-way ANOVA, Fisher's LSD post-hoc	genotype x treatment interaction: $F_{1,29}$=2.469 P=0.1270<br><br>genotype effect: $F_{1,29}$=0.0573 P=0.8124<br><br>treatment effect: $F_{1,29}$=33.82 P<0.0001	WT GFP vs. WT ERK2, p<0.01<br><br>$Hdh^{Q111/+}$ GFP vs $Hdh^{Q111/+}$ ERK2, P<0.0001	WT GFP, n=10<br><br>WT ERK2, n=7<br><br>HdhQ111/+ GFP, n=9<br><br>HdhQ111/+ ERK2, n=7
7F	Two-way ANOVA, Fisher's LSD post-hoc	genotype x treatment interaction: $F_{1,29}$=4.831 P<0.05<br><br>treatment effect: $F_{1,29}$=11.26 P<0.01<br><br>genotype effect: $F_{1,29}$=0.5528 P=0.4632	WT GFP vs $Hdh^{Q111/+}$ GFP, P<0.05<br><br>$Hdh^{Q111/+}$ GFP vs. $Hdh^{Q111/+}$ ERK2, P<0.001	WT GFP, n=10<br><br>WT ERK2, n=7<br><br>HdhQ111/+ GFP, n=9<br><br>HdhQ111/+ ERK2, n=7
7G	Two-way ANOVA, Fisher's LSD post-hoc	genotype x treatment interaction: $F_{1,29}$=2.658 P=0.1138<br><br>genotype effect: $F_{1,29}$=10.73 P<0.01<br><br>treatment effect: $F_{1,29}$=2.815 P=0.1041	WT GFP vs. $Hdh^{Q111/+}$ GFP, P<0.001<br><br>$Hdh^{Q111/+}$ GFP vs. $Hdh^{Q111/+}$ ERK2, P<0.05	WT GFP, n=10<br><br>WT ERK2, n=7<br><br>HdhQ111/+ GFP, n=9<br><br>HdhQ111/+ ERK2, n=7
7H	Independent samples t test	t_{14}=14.71 P<0.0001		HdhQ111/+ GFP, n=9<br><br>HdhQ111/+ ERK2, n=7
