# Supplementary material for: Nuclear ERK1/2 signaling potentiation enhances neuroprotection and cognition via Importinα1/KPNA2
Source: EMBO Mol Med. 2023 Oct 4;15(11):e15984. doi: 10.15252/emmm.202215984 (PMC10630888; doi:10.15252/emmm.202215984)
Supplement: Supplementary file 2 — Expanded View Figures PDF [file EMMM-15-e15984-s009.pdf]

Expanded View Figures

Figure EV1. ERK1/ERK2 MAP kinase ratio controls rotational behavior and striatal spine density.

A Western blot analysis of striatal extracts obtained from adult mice after knockdown of ERK1 or ERK2. shRNA ERK1 and shRNA ERK2 specifically reduced ERK1 and ERK2 protein levels, respectively (*n* = 5 mice per group).

B–G Spontaneous rotational behavior measured after knockdown or overexpression of ERK1 and ERK2, or their chimeric constructs, 2 weeks (2w) post-LV injection (*n* = 13–29 mice per group). (B) Control mice (bilateral striatal injection of ctr shRNA) showed an equal number of 180° rotations to either side. Unilateral expression of shRNA ERK1 (combined with contralateral ctr shRNA injection) induced net contralateral rotations (negative values), whereas shRNA ERK2 induced increased ipsilateral rotations (positive values). (E) Area under the curve (AUC) analysis over 10 days. (C) Mice unilaterally overexpressing ERK1 or ERK2 > 1 showed increased ipsilateral rotations (positive values). (F) AUC analysis over 10 days. (D) Mice overexpressing ERK2 or ERK1 > 2 showed net contralateral rotations (negative values) compared to control mice (G) AUC analysis over 10 days.

H Representative images of dendritic spines on striatal neurons after knockdown or overexpression of ERK1, ERK2, or their chimeric constructs.

I Neurons of mice overexpressing ERK1 or ERK2 > 1 showed decreased spine density, while mice overexpressing ERK1 > 2 and mice with ERK1 downregulated showed increased spine density. Data were obtained from 3 to 9 mice per group. The mean for each group is represented by the solid diamonds, the median by the horizontal bars in the box plot, the box upper and lower edges are the 75% limits and the whiskers are the 90% limits.

Data information: Results show mean ± s.e.m. ☆$P < 0.0001$, ★★★$P < 0.001$, ★★$P < 0.01$, ★$P < 0.05$. A full statistical analysis is reported in Appendix Table S1. Scale bar: 5 µm.
Source data are available online for this figure.

 © 2023 The Authors

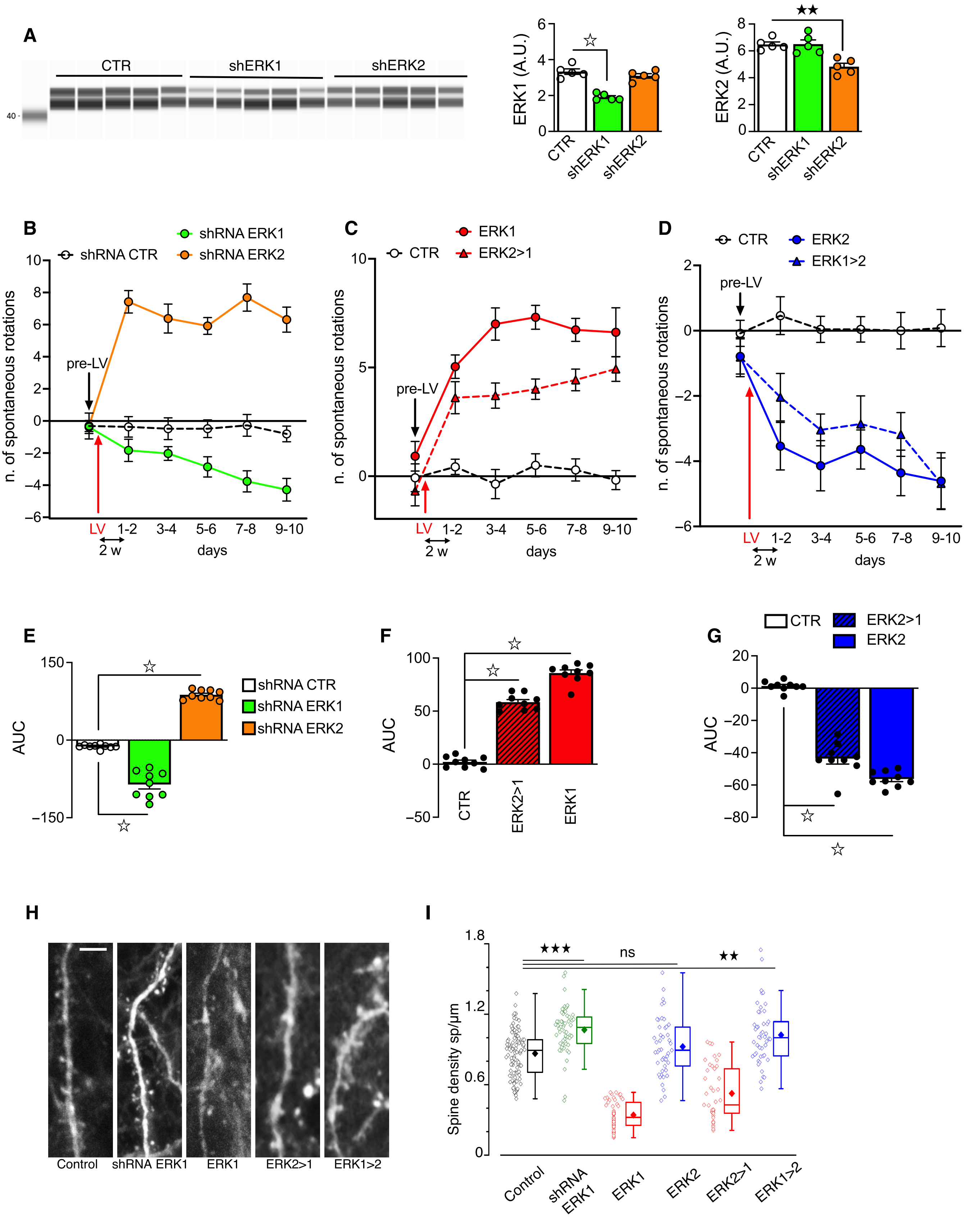
A
CTR
shERK1
shERK2
40
ERK1 (A.U.)
ERK2 (A.U.)
CTR
shERK1
shERK2
B
shRNA CTR
shRNA ERK1
shRNA ERK2
n. of spontaneous rotations
pre-LV
LV
1-2
3-4
5-6
7-8
9-10
2 w
days
C
CTR
ERK1
ERK2>1
D
CTR
ERK2
ERK1>2
E
AUC
shRNA CTR
shRNA ERK1
shRNA ERK2
F
CTR
ERK2>1
ERK1
G
CTR
ERK2>1
ERK2
H
Control
shRNA ERK1
ERK1
ERK2>1
ERK1>2
I
Spine density sp/µm
ns
Control
shRNA ERK1
ERK1
ERK2
ERK2>1
ERK1>2

Figure EV1.

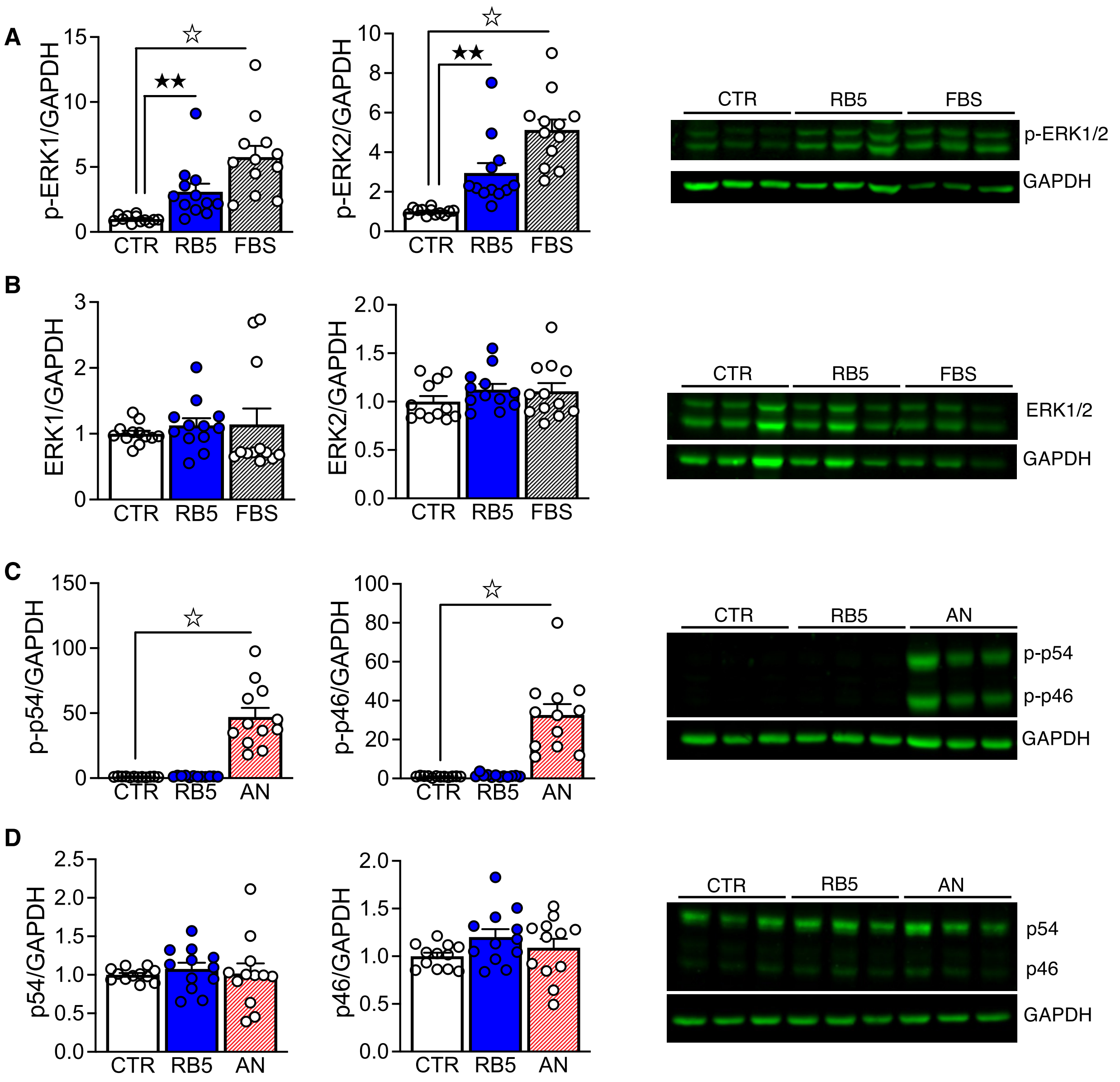

Figure EV2. RB5 specifically activates ERK1 and ERK2 in HEK293 cells.

A, B HEK293 cells were treated with RB5 50 μM, or FBS 20% (positive control) for 15 min, or untreated (CTR, negative control). Data were obtained from two independent experiments (n = 12 independent samples per group). RB5 treatment significantly increased ERK1 and ERK2 phosphorylation (A, left panel) with no effect on total ERK1 and ERK2 levels (B, left panel). (A, B, right panels) Representative Western blots of pERK1/2 and total ERK1/2.

C, D HEK293 cells were treated with RB5 50 μM or anisomycin 5 μM (AN) as a positive control for 30 min or untreated cells (CTR, negative control). Data were obtained from two independent experiments (n = 12 independent samples per group). RB5 treatment did not affect either phospho-JNK p54 and p46 (C, left panel) or total JNK p54 and p46 levels (D, left panel). (C, D, right panels) Representative Western blots of phospho-JNKs and total JNKs.

Data information: Results show mean ± s.e.m. $^{*}P < 0.0001$, $^{**}P < 0.01$. A full statistical analysis is reported in Appendix Table S1.
Source data are available online for this figure.

© 2023 The Authors

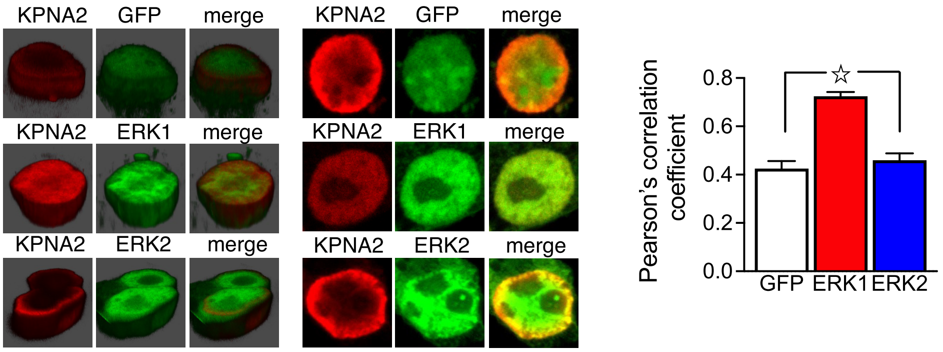

Figure EV3. KPNA2 preferentially colocalizes with ERK1.

HEK293 cells were transfected with GFP, ERK1-GFP, or ERK2-GFP together with KPNA2-T7 (left panel). Representative tridimensional and bidimensional images (right panel). Pearson's correlation coefficient was increased in ERK1-transfected cells. Data were obtained from n = 83–90 cells per group. Results show a mean ± s.e.m. *P < 0.0001. A full statistical analysis is reported in Appendix Table S1.

Source data are available online for this figure.

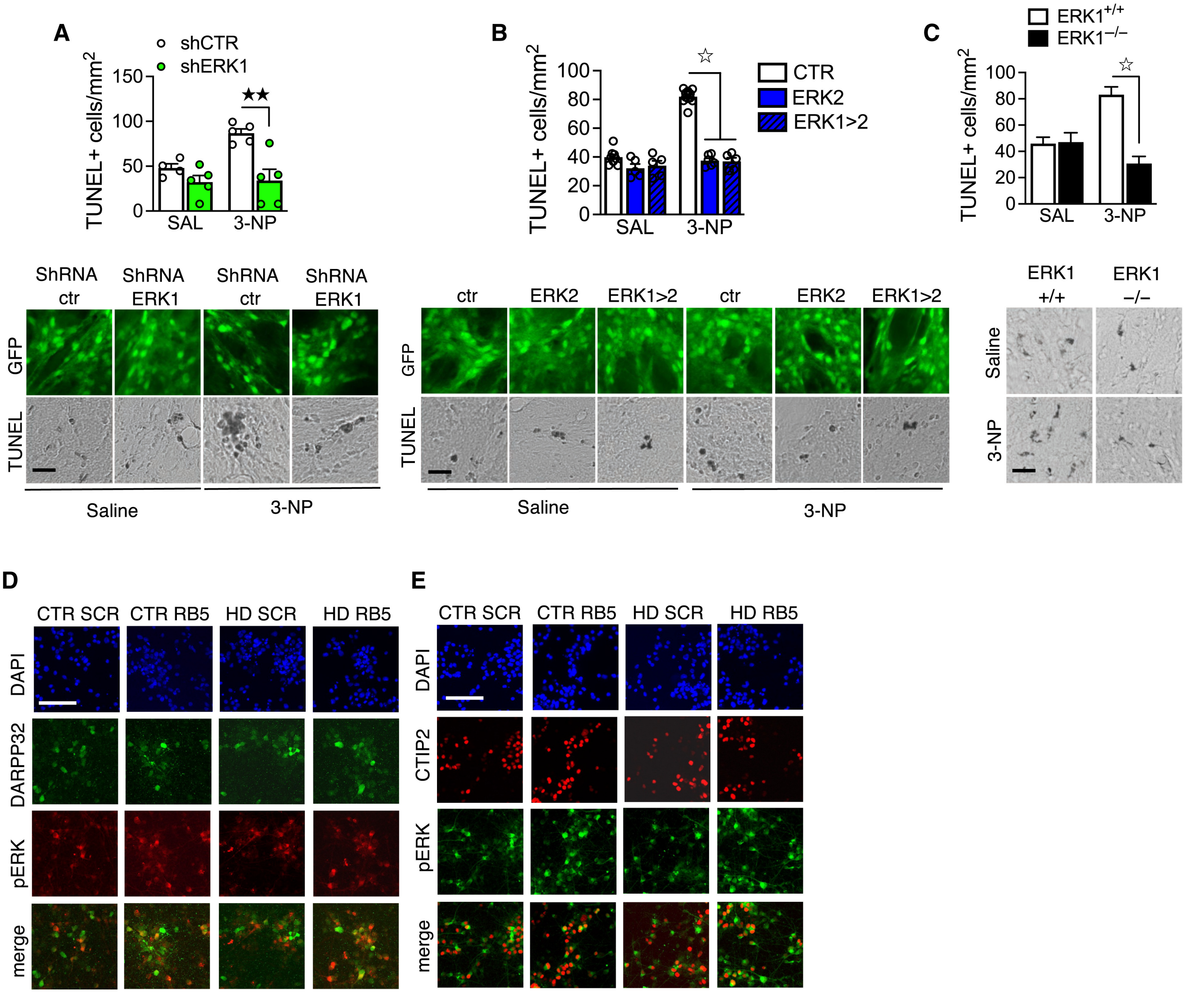

Figure EV4. ERK1 loss and ERK2 overexpression protect striatal neurons from 3-NP-induced apoptosis.

A, B (Bottom panels) Representative images of the TUNEL assay in mice with *in vivo* striatal knockdown of ERK1 (A) or overexpression of ERK2 and ERK1 > 2 (B). (top panels) TUNEL assay performed after 21 days of sub chronic treatment with 3-NP (50 mg/kg, i.p. once a day) or saline. shRNA ERK1 protected striatal neurons from 3-NP-induced apoptosis (A, top panel). Similarly, ERK2 or ERK1 > 2 overexpression protected against 3-NP-induced apoptosis (B, top panel). Data were obtained from $n = 4$–10 mice per group.

C (Bottom panel) Representative images of the TUNEL assay in the striatum of ERK1$^{-/-}$ mice injected either with saline or 3-NP (50 mg/kg, i.p. once a day) for 21 days. (top panel) ERK1$^{-/-}$ mice were protected against the 3-NP-induced striatal neurotoxicity. Data were obtained from $n = 9$–13 mice per group.

D Representative images of pERK1/2 and DARRP-32 immunofluorescence in HD lines and isogenic controls.

E Representative images of pERK1/2 and CTIP-2 in HD lines and isogenic controls.

Data information: Scale bars: 50 µm. Results show a mean ± s.e.m. $^{*}P < 0.0001$, $^{**}P < 0.01$. A full statistical analysis is reported in Appendix Table S1.
Source data are available online for this figure.

© 2023 The Authors

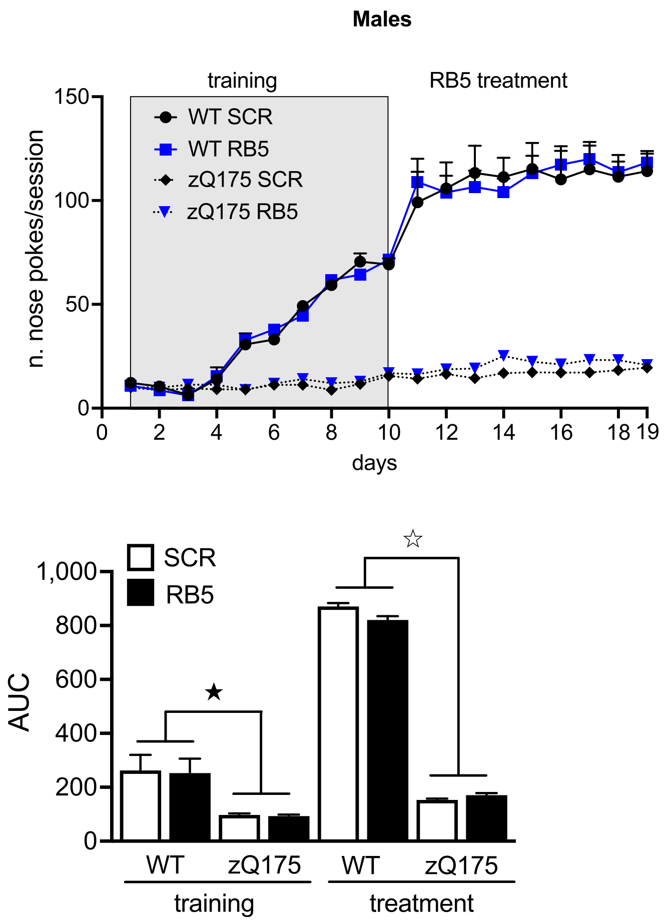

Figure EV5. RB5 administration does not counteract learning deficits in zQ175 males.

(Top panel) A clear impairment in zQ175 male mice was detected from day 1 to day 10 of the FR1 training. However, RB5 administration did not show any effect on learning. (bottom panel) AUC analysis. WT SCR $n = 8$; WT RB5 $n = 8$; zQ175 SCR $n = 10$; zQ175 RB5 $n = 9$.

Data information: Results show a mean ± s.e.m. ☆$P < 0.0001$, ★$P < 0.05$. A full statistical analysis is reported in Appendix Table S1.
Source data are available online for this figure.
