# Supplementary material for: Nuclear ERK1/2 signaling potentiation enhances neuroprotection and cognition via Importinα1/KPNA2
Source: EMBO Mol Med. 2023 Oct 4;15(11):e15984. doi: 10.15252/emmm.202215984 (PMC10630888; doi:10.15252/emmm.202215984)
Supplement: Supplementary file 12 [file EMMM-15-e15984-s012.pdf]

EMBO Molecular Medicine

Nuclear ERK1/2 signaling potentiation enhances neuroprotection and cognition via Importin a1/KPNA2

Riccardo Brambilla, Marzia Indrigo, Ilaria Morella, Raffaele Disa, Alessandro Papale, Riccardo Parra, Antonia Gurgone, Daniela Lecca, Anna Cavaccini, Cezar Tigaret, Alfredo Cagnotto, KImberly Jones, Simon Brooks, Gian Michele Ratto, Nicholas Allen, Silvia Middei, Maurizio Giustetto, Anna Carta, Raffaella Tonini, Mario Salmona, Jeremy Hall, Kerrie Thomas, Stefania Fasano, Mariah Lelos, and Daniel Orellana
DOI: 10.15252/emmm.202215984

Corresponding authors: Riccardo Brambilla (brambillar@cf.ac.uk) , Stefania Fasano (fasanos@cardiff.ac.uk)

Review Timeline:

Submission Date:	10th Mar 22
Editorial Decision:	4th Apr 22
Revision Received:	13th Jul 23
Editorial Decision:	25th Jul 23
Revision Received:	6th Sep 23
Accepted:	7th Sep 23

Editor: Zeljko Durdevic

Transaction Report:

(Note: With the exception of the correction of typographical or spelling errors that could be a source of ambiguity, letters and reports are not edited. Depending on transfer agreements, referee reports obtained elsewhere may or may not be included in this compilation. Referee reports are anonymous unless the Referee chooses to sign their reports.)

4th Apr 2022

Dear Prof. Brambilla,

Thank you for the submission of your manuscript to EMBO Molecular Medicine. We have now received feedback from the two reviewers who agreed to evaluate your manuscript. As you will see from the reports below, the referees acknowledge the interest of the study but also raise important concerns that should be addressed in a major revision.

Further consideration of a revision that addresses reviewers' concerns in full will entail a second round of review. EMBO Molecular Medicine encourages a single round of revision only and therefore, acceptance or rejection of the manuscript will depend on the completeness of your responses included in the next, final version of the manuscript. For this reason, and to save you from any frustrations in the end, I would strongly advise against returning an incomplete revision.

We would welcome the submission of a revised version within six months for further consideration. Please let us know if you require longer to complete the revision.

I look forward to receiving your revised manuscript.

Yours sincerely,

Zeljko Durdevic

Zeljko Durdevic
Editor
EMBO Molecular Medicine

*****

When submitting your revised manuscript, please carefully review the instructions that follow below. We perform an initial quality control of all revised manuscripts before re-review; failure to include requested items will delay the evaluation of your revision.

We require:

1) A .docx formatted version of the manuscript text (including legends for main figures, EV figures and tables). Please make sure that the changes are highlighted to be clearly visible.

2) Individual production quality figure files as .eps, .tif, .jpg (one file per figure). For guidance, download the 'Figure Guide PDF': (https://www.embopress.org/page/journal/17574684/authorguide#figureformat).

3) A .docx formatted letter INCLUDING the reviewers' reports and your detailed point-by-point responses to their comments. As part of the EMBO Press transparent editorial process, the point-by-point response is part of the Review Process File (RPF), which will be published alongside your paper.

4) A complete author checklist, which you can download from our author guidelines (https://www.embopress.org/page/journal/17574684/authorguide#submissionofrevisions). Please insert information in the checklist that is also reflected in the manuscript. The completed author checklist will also be part of the RPF.

5) Please note that all corresponding authors are required to supply an ORCID ID for their name upon submission of a revised manuscript.

6) It is mandatory to include a 'Data Availability' section after the Materials and Methods. Before submitting your revision, primary datasets produced in this study need to be deposited in an appropriate public database, and the accession numbers and database listed under 'Data Availability'. Please remember to provide a reviewer password if the datasets are not yet public (see

https://www.embopress.org/page/journal/17574684/authorguide#dataavailability).

In case you have no data that requires deposition in a public database, please state so in this section. Note that the Data Availability Section is restricted to new primary data that are part of this study.

7) For data quantification: please specify the name of the statistical test used to generate error bars and P values, the number (n) of independent experiments (specify technical or biological replicates) underlying each data point and the test used to calculate p-values in each figure legend. The figure legends should contain a basic description of n, P and the test applied. Graphs must include a description of the bars and the error bars (s.d., s.e.m.). See also 'Figure Legend' guidelines: https://www.embopress.org/page/journal/17574684/authorguide#figureformat

8) We would also encourage you to include the source data for figure panels that show essential data. Numerical data should be provided as individual .xls or .csv files (including a tab describing the data). For blots or microscopy, uncropped images should be submitted (using a zip archive if multiple images need to be supplied for one panel). Additional information on source data and instruction on how to label the files are available at

.

9) Our journal encourages inclusion of *data citations in the reference list* to directly cite datasets that were re-used and obtained from public databases. Data citations in the article text are distinct from normal bibliographical citations and should directly link to the database records from which the data can be accessed. In the main text, data citations are formatted as follows: "Data ref: Smith et al, 2001" or "Data ref: NCBI Sequence Read Archive PRJNA342805, 2017". In the Reference list, data citations must be labeled with "[DATASET]". A data reference must provide the database name, accession number/identifiers and a resolvable link to the landing page from which the data can be accessed at the end of the reference. Further instructions are available at .

10) We replaced Supplementary Information with Expanded View (EV) Figures and Tables that are collapsible/expandable online. A maximum of 5 EV Figures can be typeset. EV Figures should be cited as 'Figure EV1, Figure EV2" etc... in the text and their respective legends should be included in the main text after the legends of regular figures.

- For the figures that you do NOT wish to display as Expanded View figures, they should be bundled together with their legends in a single PDF file called *Appendix*, which should start with a short Table of Content. Appendix figures should be referred to in the main text as: "Appendix Figure S1, Appendix Figure S2" etc.

- Additional Tables/Datasets should be labeled and referred to as Table EV1, Dataset EV1, etc. Legends have to be provided in a separate tab in case of .xls files. Alternatively, the legend can be supplied as a separate text file (README) and zipped together with the Table/Dataset file.

See detailed instructions here:

.

11) The paper explained: EMBO Molecular Medicine articles are accompanied by a summary of the articles to emphasize the major findings in the paper and their medical implications for the non-specialist reader. Please provide a draft summary of your article highlighting

- the medical issue you are addressing,

- the results obtained and

- their clinical impact.

This may be edited to ensure that readers understand the significance and context of the research. Please refer to any of our published articles for an example.

12) For more information: There is space at the end of each article to list relevant web links for further consultation by our readers. Could you identify some relevant ones and provide such information as well? Some examples are patient associations, relevant databases, OMIM/proteins/genes links, author's websites, etc...

13) Author contributions: the contribution of every author must be detailed in a separate section (before the acknowledgments).

14) A Conflict of Interest statement should be provided in the main text.

15) Every published paper now includes a 'Synopsis' to further enhance discoverability. Synopses are displayed on the journal webpage and are freely accessible to all readers. They include a short stand first (maximum of 300 characters, including space) as well as 2-5 one-sentences bullet points that summarizes the paper. Please write the bullet points to summarize the key NEW findings. They should be designed to be complementary to the abstract - i.e. not repeat the same text. We encourage inclusion of key acronyms and quantitative information (maximum of 30 words / bullet point). Please use the passive voice. Please attach these in a separate file or send them by email, we will incorporate them accordingly.

Please also suggest a striking image or visual abstract to illustrate your article as a PNG file 550 px wide x 300-600 px high.

EMBO Molecular Medicine has a "scooping protection" policy, whereby similar findings that are published by others during review or revision are not a criterion for rejection. Should you decide to submit a revised version, I do ask that you get in touch after three months if you have not completed it, to update us on the status.

***** Reviewer's comments *****

Referee #1 (Remarks for Author):

Indrigo et al. study the role of Erk1/2 signaling in neuronal plasticity and survival. They report RB5, a peptide that represents AA 7-39 of Erk1 and thus affects the translocation of Erk1/2 to the nucleus via a mechanism that depends on binding of RB5 to the importin KPNA2, which eventually leads to enhanced ERK2 nuclear levels. They also provide data on various models for neurodegenerative diseases.
Overall this is a very interesting and timely study that goes a long way to design and test a novel approach to modulate Erk signaling to treat neurodegeneration. The authors report a large amount of data, which in the other hand is also a bit of a problem. The figures and thus also its legends are enormous and sometimes difficult to digest. The way the paper is currently written sometimes appears a bit like a mixture of preliminary results. I think, the manuscript would befit also from restructuring by preparing for example some of the data as EV figures and at the same time strengthening the key findings.

Major points.

1. Fig. 1. Althought the authors refer to previous publications in which the employed shRNAs/shRNAmirs targeting specifically Erk1 or Erk2 have been used, it would be important to show that these constructs work and are expressed as expected in the various in vitro and in vivo systems employed in this study.

2. Call out for Fig 1D is missing from the figure legend. Moreover, it would be nice to show representative images of the TUNEL staining for the striatal data. Specifically, the protective effect of Erk1 knock down is difficult to appreciate, which is of course not too surprising taken into account that healthy wild type mice are analyzed.

3. Fig 1 F-K. How many cells are affected after viral injection? Also, since behavioral alterations are already observed 1-2 days after injection for most conditions, are 1-2 days really sufficient to establish proper expression of the corresponding constructs? This seems to be in contrast to the in vitro data from cortical neurons in which no effect was observed after 3 days.

4. Generally, the spontaneous rotation and spine density experiments shown in Fig 1 need to be explained better. The figure legends are extensive and while its important to communicate the detailed statistics, other key information on the experiments is missing. For example, the methods section states that behavior was assayed 2 weeks after injection but the figures indicate that the measurements took place from 1-10 days post-injection. As for the spine density its stated in the methods that the experiments were performed 4 weeks post-injection, which does not match the behavioral analysis? I could also not find the details on how spines were visualized? Additionally, it would be interesting to match the in vitro and in vivo data and perform spine analysis in 10 day old cortical neurons treated with the various constructs 3 days after treatment, since apoptosis is not yet detected.

5. Fig 2 H-K. please provide a better legend to understand what the red, green and merged stainings indicate. Also, a biochemical approach to compare for example the nuclear vs. cytoplasmatic Erk1/2 levels along with the nuclear targets would certainly improve the message to be communicated by these experiments.

6. Fig. 3. Why did the authors selected 20mg/kg for the analysis in Fig 3A-B, when Fig.3C suggest that a dose of 10mk/kg appears to be equally effective?

7. Fig 3 F, K, L. Please provide color key for the stainings.

8. It appears that, the data shown in Fig. 4 depend on the overexpression in Erk1/2 construction in HEK cells. It would be interesting to show that the suggesting effect of RB5 on Erk1 and Erk2 binding to KPNA2 and IPO7 also occurs in the relevant

tissues/cells under physiological conditions.

9. Fig.5.it is stated that Erk1 KO mice are analyzed but I think the authors show the shRNA mediated knock down of Erk1.

10. The 3-NP experiments may need some more explanation. I believe these represent viral-mediated expressions of the constructs. How many cells are affected? I guess only the cells that express shRNA for Erk1 should be protected but how does this relate then to Tunnel staining? Are we indeed looking at neurons? Moreover, a highly mosaic expression of the constructs would affect the results and I don't believe that the whole striatum is affected by viral injection. Morover, most of the data relies on semiquantitative analysis when it comes to the disease models, while functional analysis of synaptic plasticity is the carried out in healthy mice?

11. All of the effects in the neurodegenerative models are difficult to compare and interpret since - as far as I understand - treatment with viral constructs were carried out before 3-NP or MPTP injection and the tissues were analyzed 21 days later, while RB5 was injected for 7days in the transgenic mouse models.

12. Along the same line as already addressed in points 10-11, after studying various degeneration and WT models using different paradigms its somewhat surprising to see that one single i.p. injection of RB5 improves associative fear learning in TG2576 mice? How does this observation relate to the other phenotypes described before? This cannot be linked to neuroprotection but may reflect altered synaptic plasticity, which is however only studied in WT mice.

Minor points

1. I would not call the TG2576 mice a model for MCI.

Referee #2 (Comments on Novelty/Model System for Author):

Models are ok but there are some issues with their introduction (see general comments)

Referee #2 (Remarks for Author):

Indrigo et al. proposes a mechanism by which Importina1/KPNA2-mediated nuclear ERK1/2 signalling potentiation would prevent neurodegeneration and facilitate cognitive enhancement. This manuscript contains a massive amount of experiments and data. Although it is complex to go through all experiments, authors should be commended for their efforts in making them understandable, not neglecting the fine details while keeping in mind the big picture. The key demonstration is that disrupting ERK1 binding to KPNA2 using a competing peptide allows nuclear translocation of ERK2, a move that would be the key to the subsequent cascade of transcriptomic events leading to neuroprotection and electrical activity preservation/restoration in various settings. Such shared mechanisms (as are anyway the MAPK cascades) would thus be beneficial to a number of neurological conditions.

Overall this reviewer wishes to express only minor concerns although the use of non-neuronal cell lines or primary cultures in some experiments are disappointing in such a beautifully constructed work (this is especially true for instance for interacting studies where KPNA2 is identified as a partner in HEK293 cells).

Some limitations related to the chosen models or at least to the way they are introduced and some others related to the rather wide understanding of "cognition"... One of the most interesting results is the demonstration of enhanced performance in the NOR test of wild-type normal mice exposed to RB5... Although extremely interesting and exciting, this result is far from demonstrating an improvement of "cognition" overall. Spatial memory maybe...Same issue with AD Tg2576 mice tested in one test, the contextual fear conditioning, and yet authors claim about an improvement of MCI...

Figures are a bit crowed at the expense of readability. "representative " examples are unreadable at printed size and must be magnified on a screen to benefit from their representativeness. Would it be possible to move to supplementary data the representative examples?

N of in vitro cultures are not particularly telling. To what do they refer to? Number of slides? How were controlled the number of MEF or cortical primary neurons on the slides?

Doubtful that in vivo LV injections were of 2 ml.... (page 14)

Methodology for counting tunel+ cells in vivo is not described. Did authors check if the volume of transfection were comparable between groups?

Dose response of RB5 is not mentioned in the methods, only in the results section.

PLA should ideally be accompanied by biochemistry (e.g. co-IP) for ascertaining protein-protein interactions.

Animal models of neurodegenerative diseases would benefit from a more honest presentation. Not that they are not relevant for they are not models of HD, PD or AD. They model certain aspects of these conditions. The distinction between pathocopic (e.g. neurotoxins) and etiogenic (e.g. transgenics) models of HD and PD should for instance be made. 3-NP and MPTP models are in no way models of PD (regimen of intoxication are by the way not conventional). They model neurotoxin-induced MSN or DA cell death (which certainly occur in these diseases but unlikely through that mechanisms). More than model of diseases they model loss of neurons also observed in these diseases. Such a distinction would NOT diminish the impact of the work but would be more honest in presenting the putative relevance to these diseases etiology.

The quantification methodology is unclear and the possibility of a rostro-caudal effect within the striatum is not even envisioned. In the SNc, full stereology should be applied as it should be for striatum as well. Focusing on TUNEL+ cells is understood but should be accompanied by MSN (striatum) and DA neurons (SNc) countings (Fig 5L is meaningless).

Tg2576 mice overexpressing human APP695 with the "Swedish" mutation develop memory deficits and plaques with age, making them popular for examining the relationship between Aβ and memory. Tg2576 mice show rapid increases in Aβ starting at ~6 months and amyloid plaques beginning at 9-12 months. Therefore why choosing 7-months of age in some behavioral experiments (note that age is not mentioned at all in experiments presented on figure 5) ?

Response to Reviewers

Referee #1:

Indrigo et al. study the role of Erk1/2 signaling in neuronal plasticity and survival. They report RB5, a peptide that represents AA 7-39 of Erk1 and thus affects the translocation of Erk1/2 to the nucleus via a mechanism that depends on binding of RB5 to the importin KPNA2, which eventually leads to enhanced ERK2 nuclear levels. They also provide data on various models for neurodegenerative diseases.
Overall, this is a very interesting and timely study that goes a long way to design and test a novel approach to modulate Erk signaling to treat neurodegeneration. The authors report a large amount of data, which in the other hand is also a bit of a problem. The figures and thus also its legends are enormous and sometimes difficult to digest. The way the paper is currently written sometimes appears a bit like a mixture of preliminary results. I think, the manuscript would befit also from restructuring by preparing for example some of the data as EV figures and at the same time strengthening the key findings.

We thank Referee #1 for the very positive comments. We agree that the original version of the manuscript was difficult to follow, also considering the amount of data provided in the main figures. We have significantly changed the format, by introducing EV figures and moving most of the detailed statistical analysis in the appendix. We hope that this will increase the readability and improve the communication of the key messages of the paper.

1. Fig. 1. Although the authors refer to previous publications in which the employed shRNAs/shRNAmirs targeting specifically Erk1 or Erk2 have been used, it would be important to show that these constructs work and are expressed as expected in the various in vitro and in vivo systems employed in this study.

The reviewer requested to provide evidence that the shRNAs used in our study do indeed specifically downregulate ERK1 or ERK2 proteins in vivo, as originally published in cell culture (Indrigo et al, 2010). We performed the requested control study by injecting lentiviral vectors expressing selective small hairping RNAs (LV-shRNA) in the mouse striatum to quantify the extent of protein downregulation, for both ERK1 and ERK2, measured by western blotting (EV1). As expected, we observed a significant reduction of $p44^{ERK1}$ and $p42^{ERK2}$ bands but only in the presence of their specific LV-shRNA. That experiment confirms that the knockdown approach, albeit less efficient than in cell culture, is still working and generating opposite changes in cell survival in vivo (Fig.1). The observed in vivo reduction in p44 and p42 levels is likely to be an underestimation due to the significant presence of non-infected cells in the tissue samples and the use of western blotting, the only method able to distinguish the two bands, as previously reported in other occasions (Fig. 1C and Bido et al, 2015). The “apparent” reduced “efficiency” in reducing ERK2 level is also likely to be due to a negative selection associated to the pro-apoptotic effect of this manipulation. We believe these additional control data will satisfy the reviewer.

2. Call out for Fig 1D is missing from the figure legend. Moreover, it would be nice to show representative images of the TUNEL staining for the striatal data. Specifically, the protective effect of Erk1 knock down is difficult to appreciate, which is of course not too surprising taken into account that healthy wild type mice are analysed.

We do apologise the incomplete Fig 1D and E. We have corrected the error and provided the representative figures of the staining for both experiments. We do agree that the neuroprotective effect of ERK1 knock-down is not very pronounced but still statistically significant, so we believe it is important to show it in contrast to the opposite effect of the ERK2 knock-down.

3. Fig 1 F-K. How many cells are affected after viral injection? Also, since behavioral alterations are already observed 1-2 days after injection for most conditions, are 1-2 days really sufficient to establish proper expression of the corresponding constructs? This seems to be in contrast to the in vitro data from cortical neurons in which no effect was observed after 3 days.

We do apologise for the confusion. We have amended the figure and better explained the experimental procedure in the material and methods section on how we generated data in the original Fig. 1 F-K (rotational behaviour) and Fig.1 L-M (striatal spine density). In that experiment,

behavioural observation started 2 weeks after LV injection, to allow full construct expression. At the end of the 10 days observation, animals were perfused and striata analysed for spine imaging. All those data are now in EV1. The experimental timeframe has been now better clarified in the methods and in figure EV1.

4. Generally, the spontaneous rotation and spine density experiments shown in Fig 1 need to be explained better. The figure legends are extensive and while its important to communicate the detailed statistics, other key information on the experiments is missing. For example, the methods section states that behavior was assayed 2 weeks after injection but the figures indicate that the measurements took place from 1-10 days post-injection. As for the spine density its stated in the methods that the experiments were performed 4 weeks post-injection, which does not match the behavioral analysis? I could also not find the details on how spines were visualized? Additionally, it would be interesting to match the in vitro and in vivo data and perform spine analysis in 10 day old cortical neurons treated with the various constructs 3 days after treatment, since apoptosis is not yet detected.

Please see also the response to point 3. We believe we have clarified the procedure for LV infection, behavioural analysis, and spine measurements in the striatum. Concerning the spine analysis of cortical neurons, we agree that is an interesting experiment, but we also believe that is not directly related to the observed rotational behaviour. To avoid further confusion, we included two paragraphs in the material and methods section, one for the LV injection for rotational behaviour (Fig. 1EV) and overexpression/knockdown studies for TUNEL assay (Fig. EV5 A-B), another one for ERK2 overexpression in the HdhQ111 mice (Fig.6).

5. Fig 2 H-K. please provide a better legend to understand what the red, green and merged stainings indicate. Also, a biochemical approach to compare for example the nuclear vs. cytoplasmatic Erk1/2 levels along with the nuclear targets would certainly improve the message to be communicated by these experiments.

We apologise for the missing information. We have now specified in the figure legend what the colours indicate. Regarding ERK1/2 distribution, data showed in Figure 2B and 2C demonstrate that ERK1/2 relocates to the nucleus after RB5 treatment. We do believe that biochemical approaches are unlikely to provide a complete separation between the nuclear and cytoplasmic compartments. That is also particularly relevant considering the transient and dynamic interactions between importins and ERK1/2 that can be more easily monitored by measuring compartment specific kinase targets and confocal microscopy.

6. Fig. 3. Why did the authors selected 20mg/kg for the analysis in Fig 3A-B, when Fig.3C suggest that a dose of 10mk/kg appears to be equally effective?

We decided to use the second lowest most effective dose tested throughout the manuscript, also to be consistent with the behavioural experiments.

7. Fig 3 F, K, L. Please provide color key for the stainings.

We have now specified the colours of the staining in the figure legend.

8. It appears that, the data shown in Fig. 4 depend on the overexpression in Erk1/2 construction in HEK cells. It would be interesting to show that the suggesting effect of RB5 on Erk1 and Erk2 binding to KPNA2 and IPO7 also occurs in the relevant tissues/cells under physiological conditions.

Referee #1 here raised concerns about the physiological relevance of the KPNA2 and Erk1/Erk2 interactions, considering that all experiments were performed in tissue culture cells using a mild overexpression of epitope-tagged constructs (see Fig. 4). First, we would like to reiterate the importance to use tagged constructs for the comparative analysis since this is the only method to quantitatively assess protein binding with techniques such as proximity ligation assay. Assessing native protein interactions, as suggested by the Referee #1, is not feasible due to the current lack of selective antibodies for Erk1 and Erk2 for immunostaining studies. In addition, using different antibodies for each native importin could hamper a comparative assessment due to potential differences in detection sensitivity. However, we acknowledge the referee’s suggestion to support our binding data with additional functional data and we propose another approach to address this

point. We did manage to validate antibodies working in cells against KPNA2 using western blotting (Fig. 4F). However, the signal produced by KPNA2 antibody using immunofluorescence techniques in HEK293 cells is too weak to be quantified. Therefore, we decided to quantify the nuclear localisation and activation in tissue culture cells by measuring the nuclear localisation of ERK1/2 kinases by knocking down KPNA2 and IPO7 (Fig. 4F), the two major importins involved in the process. As shown in Fig. 4G, knockdown of either KPNA2 and IPO7 results in a complete downregulation of phosphorylated ERK signal in the nucleus, consistent with their role. RB5 treatment, did not exert any stimulatory effect on pERK nuclear localisation, as expected. However, we found a very significant difference in the intensity of total ERK1/2 signal between KPNA2 and IPO7. In fact, KPNA2 knockdown led to a RB5 independent accumulation of ERK signal in the nucleus. This latter evidence, strongly indicate that KPNA2 is not only essential for nuclear entering of ERKs but also for their exit, a process currently still poorly understood. On the contrary, IPO7, does not seem to be implicated in nuclear exit since its knockdown does not impact of total nuclear ERK levels.

9. Fig.5.it is stated that Erk1 KO mice are analyzed but I think the authors show the shRNA mediated knock down of Erk1.

We do apologise for not having been clearer. Three sets of experiments have been performed with 3-NP. In Fig. 5A we knock-down ERK1 with LVs, in Fig. 5B we overexpressed ERK2 and ERK1>2, showing a similar neuroprotective effect and final Fig. 5C was done on ERK1 KO mice. Altogether those results show the same neuroprotective effect toward 3-NP. We have now rephrased the text and the figure legend, and all those data have been moved in EV5 while the data on RB5 remained in Fig.5.

10. The 3-NP experiments may need some more explanation. I believe these represent viral-mediated expressions of the constructs. How many cells are affected? I guess only the cells that express shRNA for Erk1 should be protected but how does this relate then to Tunnel staining? Are we indeed looking at neurons? Moreover, a highly mosaic expression of the constructs would affect the results and I don't believe that the whole striatum is affected by viral injection. Morover, most of the data relies on semiquantitative analysis when it comes to the disease models, while functional analysis of synaptic plasticity is the carried out in healthy mice?

We believe that the issue has been resolved addressing point 9. Data are all consistent with a neuroprotective effect of any treatment either reducing ERK1 or increasing ERK2 levels, including the RB5 administration. 3-NP preferentially affect neurons (Fernagut et al, 2002) and we and others have shown that third generation lentiviral vectors have a marked tropisms for neurons (Papale et al, 2009, Indrigo et al, 2010, Orellana et al 2012, Bido et al, 2015). Those papers are in text cited.

11. All of the effects in the neurodegenerative models are difficult to compare and interpret since - as far as I understand - treatment with viral constructs were carried out before 3-NP or MPTP injection and the tissues were analyzed 21 days later, while RB5 was injected for 7 days in the transgenic mouse models.

Please also refer to point 9 and 10. We acknowledged that different experimental models have been subjected to different treatments, but we also believe that ALL data are consistent with a neuroprotective effect by altering the ERK1/ERK2 ratio, either by genetic intervention (with lentiviral vectors) or by a pharmacological approach (with RB5). We respectfully do believe that converging evidence significantly strengthens the conclusions of the manuscript.

12. Along the same line as already addressed in points 10-11, after studying various degeneration and WT models using different paradigms its somewhat surprising to see that one single i.p. injection of RB5 improves associative fear learning in TG2576 mice? How does this observation relate to the other phenotypes described before? This cannot be linked to neuroprotection but may reflect altered synaptic plasticity, which is however only studied in WT mice.

We acknowledge that the observation may be surprising, and we thank the reviewer for having highlighted it. However, as also pointed out by the reviewer, RB5 rapidly improves memory in both WT mice and in rats and enhances synaptic plasticity. Therefore, we believe that a single administration of RB5 to TG2576 mice acts through the same mechanism, considering that those mice are still at an early stage of the disease. We have added to the main text an additional sentence to clarify this important point. To draw a parallel to this, we also believe that the memory

enhancing effect of RB5 in the zQ175 HD mice and their controls (Fig. 6F) follows a similar mechanism.

Minor points

1. I would not call the TG2576 mice a model for MCI.

We have removed MCI from the text and used instead “early cognitive impairments”.

Referee #2:

Indrigo et al. proposes a mechanism by which Importina1/KPNA2-mediated nuclear ERK1/2 signalling potentiation would prevent neurodegeneration and facilitate cognitive enhancement. This manuscript contains a massive amount of experiments and data. Although it is complex to go through all experiments, authors should be commended for their efforts in making them understandable, not neglecting the fine details while keeping in mind the big picture. The key demonstration is that disrupting ERK1 binding to KPNA2 using a competing peptide allows nuclear translocation of ERK2, a move that would be the key to the subsequent cascade of transcriptomic events leading to neuroprotection and electrical activity preservation/restoration in various settings. Such shared mechanisms (as are anyway the MAPK cascades) would thus be beneficial to a number of neurological conditions.

Overall this reviewer wishes to express only minor concerns although the use of non-neuronal cell lines or primary cultures in some experiments are disappointing in such a beautifully constructed work (this is especially true for instance for interacting studies where KPNA2 is identified as a partner in HEK293 cells).

We would like to take here the opportunity to thank the reviewer for the extremely positive comments on the manuscript.

Some limitations related to the chosen models or at least to the way they are introduced and some others related to the rather wide understanding of "cognition"... One of the most interesting results is the demonstration of enhanced performance in the NOR test of wild-type normal mice exposed to RB5... Although extremely interesting and exciting, this result is far from demonstrating an improvement of "cognition" overall. Spatial memory maybe...Same issue with AD Tg2576 mice tested in one test, the contextual fear conditioning, and yet authors claim about an improvement of MCI...

We do agree with the reviewer that we cannot generalise the learning and memory effect observed in response to RB5. Accordingly, we did tone down the claims made in the text, both for WT mice and rats and for the Tg2576 model.

Figures are a bit crowed at the expense of readability. "representative " examples are unreadable at printed size and must be magnified on a screen to benefit from their representativeness. Would it be possible to move to supplementary data the representative examples?

We have taken advantage of the EV figures and move a significantly portion of the data in those supplementary figures. We believe that now readability has increased, thank you to the reviewer’s suggestion.

N of in vitro cultures are not particularly telling. To what do they refer to? Number of slides? How were controlled the number of MEF or cortical primary neurons on the slides?

We thank the reviewer for pointing this out. The N refers to the number of slides, as we have now clarified in the figure legends. MEF cultures have been previously described in Indrigo et al, 2010 and Orellana et al. 2012, while cortical primary neuronal cultures have been described in Mazzucchelli et al 2002. More specifically, 1.25 x 10^5 MEFs/well and 2 x 10^6 cortical neurons/well were seeded in 6 well plates. These details have now been added in the methods.

Doubtful that in vivo LV injections were of 2 ml.... (page 14)

We do apologise for the gross typo. It has now been corrected.

Methodology for counting tunel+ cells in vivo is not described. Did authors check if the volume of transfection were comparable between groups?

We apologise for this missing information. We have now clarified the technical details in the methods section for in vivo TUNEL staining. 3-4 slices per mouse were stained and TUNEL positive cells were analysed using ImageJ software. Mice were injected with 2 μl of LV per injection site, that gave comparable levels of transduction across the experimental groups.

Dose response of RB5 is not mentioned in the methods, only in the results section.

We have now added a brief paragraph in the methods about that point.

PLA should ideally be accompanied by biochemistry (e.g. co-IP) for ascertaining protein-protein interactions.

We agreed with the reviewer that co-IP could be a way forward to demonstrate protein-protein interactions. Unfortunately, we tried hard in the past and not reliable co-IP have been observed. Our conclusion is that the interactions between ERK1/2 and importins are transient, and that is consistent somehow with the dynamic process of cytoplasmic-nuclear shuttling. Despite the limitation of the approach taken, we believe that the observed differences among KPNA2, IPO 7 and other importins are genuine and we have also included in the revised version additional experiments using a knockdown strategy (see point 8 response to Reviewer #1)

Animal models of neurodegenerative diseases would benefit from a more honest presentation. Not that they are not relevant for they are not models of HD, PD or AD. They model certain aspects of these conditions. The distinction between pathocopic (e.g. neurotoxins) and etiogenic (e.g. transgenics) models of HD and PD should for instance be made. 3-NP and MPTP models are in no way models of PD (regimen of intoxication are by the way not conventional). They model neurotoxin-induced MSN or DA cell death (which certainly occur in these diseases but unlikely through that mechanisms). More than model of diseases they model loss of neurons also observed in these diseases. Such a distinction would NOT diminish the impact of the work but would be more honest in presenting the putative relevance to these diseases etiology.

We agree with the comment made by the reviewer. We have modified the text accordingly, stating that the neurotoxin drugs used in this work, 3-NP and MPTP, are simply modelling striatal and nigral degeneration, respectively.

The quantification methodology is unclear and the possibility of a rostro-caudal effect within the striatum is not even envisioned. In the SNc, full stereology should be applied as it should be for striatum as well. Focusing on TUNEL+ cells is understood but should be accompanied by MSN (striatum) and DA neurons (SNc) countings (Fig 5L is meaningless).

Referee #2 manifested here concerns about the TH staining quantification in SN of mice treated with MPTP and RB5 (Figure 5L). In the original experiment, we did not perform a full stereology counting due to the low numbers of TH positive cells in the control samples, a condition not suitable for automatic sampling by the stereology software. Thus, we decided to count all cells instead. We agree with Referee #2 that this is not optimal and thus we decided to repeat the whole experiment, including additional controls. As shown in Fig. 5G-I, MPTP treatment did reduce TH staining in the SN and that effect was fully reversed by the RB5 treatment (Fig. 5G). Importantly, neuronal cell loss in the same structure, as measured by cleaved caspase-3 staining, was also strongly attenuated by the RB5 treatment (Fig. 5H). Finally, and importantly, pERK1/2 levels were found increased in the MPTP treated samples and further enhanced by RB5, suggesting that ERK activation may indeed play a role in the cellular responses to the neurotoxic insult to MPTP, a situation previously reported also in the MPTP Non-Human Primate model (see Bezard et al, 2005, quoted in the main text). Altogether, we believe that the new MPTP data confirm a neuroprotective role of RB5 and generally ERK signalling enhancement also in the model of neurodegeneration. We believe that the stereology counting now provides convincing evidence of the RB5 neuroprotective effect on SNc neurons.

Tg2576 mice overexpressing human APP695 with the "Swedish" mutation develop memory deficits and plaques with age, making them popular for examining the relationship between Aβ and memory. Tg2576 mice show rapid increases in Aβ starting at ~6 months and amyloid plaques beginning at 9-12 months. Therefore why choosing 7-months of age in some behavioral experiments (note that age is not mentioned at all in experiments presented on figure 5)?

The scope of our experiment with the Tg2576 mouse was indeed to show whether RB5 could ameliorate early cognitive deficits at 7 months of age, upon a single drug administration. Also, we wished to determine whether the initial cell loss measured using caspase-3 would be impacted by a 7-day treatment. We agree with the reviewer that a follow up study addressing the potential impact on Aβ plaque deposition would have been important. However, we preferred to focus more on this aspect using the HD models.

25th Jul 2023

Dear Prof. Brambilla,

Thank you for the submission of your revised manuscript to EMBO Molecular Medicine. I am pleased to inform you that we will be able to accept your manuscript pending the following final amendments:

1) Authors: E-mail correspondence to Gian Michele Ratto could not be delivered. Please update his e-mail address and make sure to enter correct e-mail addresses for all authors in our submission system.
2) Figures: Please submit main and EV figures as separate high-resolution files. Renumber EV figures Figure EV1-5. Remove numbering of panels in all figures where only one panel is presented and update callouts in the main manuscript text. Please follow our figure formatting guidelines:
https://www.embopress.org/page/journal/17574684/authorguide#figureformat
3) In the main manuscript file, please do the following:
- Correct/answer the track changes suggested by our data editors by working from the attached document.
- In M&M, add statistical paragraph that should reflect all information that you have filled in the Authors Checklist, especially regarding randomization, blinding, replication.
- In data availability statement, if no data are deposited in public repositories, please add the sentence: "This study includes no data deposited in external repositories". Data availability statement should be moved to the end of M&M section.

Please check "Author Guidelines" for more information.
https://www.embopress.org/page/journal/17574684/authorguide#availabilityofpublishedmaterial
4) Appendix: Please report p-values and statistical tests used in form of a table. The table should be named Appendix Table 1 with appropriate citation in the main manuscript (e.g. figure legends). Also, please add table of content on the title page of Appendix.
5) Funding: Please make sure that information about all sources of funding are complete in both our submission system and in the manuscript. Currently information about Albero di Greta ONLUS, International Foundation for CDKL5 Research, Lou Lou Foundation and Fondazione CRT are missing in our submission system.
6) Synopsis: Every published paper now includes a 'Synopsis' to further enhance discoverability. Synopses are displayed on the journal webpage and are freely accessible to all readers. They include separate synopsis image and synopsis text.
- Synopsis image: Please provide a striking image or visual abstract as a high-resolution jpeg file 550 px-wide x (250-400)-px high to illustrate your article.
- Synopsis text: Please provide a short standfirst (maximum of 300 characters, including space) as well as 2-5 one sentence bullet points that summarise the paper as a .doc file. Please write the bullet points to summarise the key NEW findings. They should be designed to be complementary to the abstract - i.e. not repeat the same text. We encourage inclusion of key acronyms and quantitative information (maximum of 30 words / bullet point). Please use the passive voice.
- Please check your synopsis text and image before submission with your revised manuscript. Please be aware that in the proof stage minor corrections only are allowed (e.g., typos).
7) For more information: This space should be used to list relevant web links for further consultation by our readers. Could you identify some relevant ones and provide such information as well? Some examples are patient associations, relevant databases, OMIM/proteins/genes links, author's websites, etc...
8) Source data: Please upload one file per figure. Numerical data should be provided as individual .xls or .csv files (including a tab describing the data). For blots or microscopy, uncropped images should be submitted (using a zip archive if multiple images need to be supplied for one panel). Please check "Author Guidelines" for more information.
https://www.embopress.org/page/journal/17574684/authorguide#sourcedata
9) As part of the EMBO Publications transparent editorial process initiative (see our Editorial at http://embomolmed.embopress.org/content/2/9/329), EMBO Molecular Medicine will publish online a Review Process File (RPF) to accompany accepted manuscripts. This file will be published in conjunction with your paper and will include the anonymous referee reports, your point-by-point response and all pertinent correspondence relating to the manuscript. Let us know whether you agree with the publication of the RPF and as here, if you want to remove or not any figures from it prior to publication. Please note that the Authors checklist will be published at the end of the RPF.
10) Please provide a point-by-point letter INCLUDING my comments as well as the reviewer's reports and your detailed responses (as Word file).

I look forward to reading a new revised version of your manuscript as soon as possible.

Yours sincerely,

Zeljko Durdevic

Zeljko Durdevic
Editor
EMBO Molecular Medicine

*** Instructions to submit your revised manuscript ***

*** PLEASE NOTE *** As part of the EMBO Publications transparent editorial process initiative (see our Editorial at https://www.embopress.org/doi/pdf/10.1002/emmm.201000094), EMBO Molecular Medicine will publish online a Review Process File to accompany accepted manuscripts.

In the event of acceptance, this file will be published in conjunction with your paper and will include the anonymous referee reports, your point-by-point response and all pertinent correspondence relating to the manuscript. If you do NOT want this file to be published, please inform the editorial office at contact@embomolmed.org.

When submitting your revised manuscript, please include:

1) a .docx formatted version of the manuscript text (including Figure legends and tables)

2) Separate figure files*

3) supplemental information as Expanded View and/or Appendix. Please carefully check the authors guidelines for formatting Expanded view and Appendix figures and tables at
https://www.embopress.org/page/journal/17574684/authorguide#expandedview

4) a letter INCLUDING the reviewer's reports and your detailed responses to their comments (as Word file).

5) The paper explained: EMBO Molecular Medicine articles are accompanied by a summary of the articles to emphasize the major findings in the paper and their medical implications for the non-specialist reader. Please provide a draft summary of your article highlighting
- the medical issue you are addressing,
- the results obtained and
- their clinical impact.
This may be edited to ensure that readers understand the significance and context of the research.
Please refer to any of our published articles for an example.

6) For more information: There is space at the end of each article to list relevant web links for further consultation by our readers. Could you identify some relevant ones and provide such information as well? Some examples are patient associations, relevant databases, OMIM/proteins/genes links, author's websites, etc...

7) Author contributions: the contribution of every author must be detailed in a separate section.

8) EMBO Molecular Medicine now requires a complete author checklist (https://www.embopress.org/page/journal/17574684/authorguide) to be submitted with all revised manuscripts. Please use the checklist as guideline for the sort of information we need WITHIN the manuscript. The checklist should only be filled with page numbers were the information can be found. This is particularly important for animal reporting, antibody dilutions (missing) and exact values and n that should be indicted instead of a range.

9) Every published paper now includes a 'Synopsis' to further enhance discoverability. Synopses are displayed on the journal webpage and are freely accessible to all readers. They include a short stand first (maximum of 300 characters, including space) as well as 2-5 one sentence bullet points that summarise the paper. Please write the bullet points to summarise the key NEW findings. They should be designed to be complementary to the abstract - i.e. not repeat the same text. We encourage inclusion of key acronyms and quantitative information (maximum of 30 words / bullet point). Please use the passive voice. Please attach these in a separate file or send them by email, we will incorporate them accordingly.

You are also welcome to suggest a striking image or visual abstract to illustrate your article. If you do please provide a jpeg file

550 px-wide x 400-px high.

10) A Conflict of Interest statement should be provided in the main text

11) Please note that we now mandate that all corresponding authors list an ORCID digital identifier. This takes <90 seconds to complete. We encourage all authors to supply an ORCID identifier, which will be linked to their name for unambiguous name identification.

Currently, our records indicate that the ORCID for your account is 0000-0003-3569-5706.

Please click the link below to modify this ORCID:
Link Not Available

12) The system will prompt you to fill in your funding and payment information. This will allow Wiley to send you a quote for the article processing charge (APC) in case of acceptance. This quote takes into account any reduction or fee waivers that you may be eligible for. Authors do not need to pay any fees before their manuscript is accepted and transferred to our publisher.

*Additional important information regarding Figures

Each figure should be given in a separate file and should have the following resolution:
Graphs 800-1,200 DPI
Photos 400-800 DPI
Colour (only CMYK) 300-400 DPI"

Figures are not edited by the production team. All lettering should be the same size and style; figure panels should be indicated by capital letters (A, B, C etc). Gridlines are not allowed except for log plots. Figures should be numbered in the order of their appearance in the text with Arabic numerals. Each Figure must have a separate legend and a caption is needed for each panel.

*Additional important information regarding figures and illustrations can be found at https://bit.ly/EMBOPressFigurePreparationGuideline. See also figure legend preparation guidelines: https://www.embopress.org/page/journal/17574684/authorguide#figureformat

The system will prompt you to fill in your funding and payment information. This will allow Wiley to send you a quote for the article processing charge (APC) in case of acceptance. This quote takes into account any reduction or fee waivers that you may be eligible for. Authors do not need to pay any fees before their manuscript is accepted and transferred to our publisher.

***** Reviewer's comments *****

Referee #2 (Comments on Novelty/Model System for Author):

Authors addressed my concerns

Referee #2 (Remarks for Author):

The authors satisfactorily addressed this reviewer's concerns.

The authors addressed the remaining editorial issues.

7th Sep 2023

Dear Prof. Brambilla,

We are pleased to inform you that your manuscript is accepted for publication and is now being sent to our publisher to be included in the next available issue of EMBO Molecular Medicine.

Please read below for additional IMPORTANT information regarding your article, its publication and the production process.

Congratulations on your interesting work,

Zeljko Durdevic

Zeljko Durdevic
Editor
EMBO Molecular Medicine

Follow us on Twitter @EmboMolMed
Sign up for eTOCs at embopress.org/alertsfeeds

*** *** *** IMPORTANT INFORMATION *** *** ***

SPEED OF PUBLICATION
The journal aims for rapid publication of papers, using using the advance online publication "Early View" to expedite the process: A properly copy-edited and formatted version will be published as "Early View" after the proofs have been corrected. Please help the Editors and publisher avoid delays by providing e-mail address(es), telephone and fax numbers at which author(s) can be contacted.

Should you be planning a Press Release on your article, please get in contact with embomolmed@wiley.com as early as possible, in order to coordinate publication and release dates.

LICENSE AND PAYMENT:

All articles published in EMBO Molecular Medicine are fully open access: immediately and freely available to read, download and share.

EMBO Molecular Medicine charges an article processing charge (APC) to cover the publication costs. You, as the corresponding author for this manuscript, should have already received a quote with the article processing fee separately. Please let us know in case this quote has not been received.

Once your article is at Wiley for editorial production you will receive an email from Wiley's Author Services system, which will ask you to log in and will present you with the publication license form for completion. Within the same system the publication fee can be paid by credit card, an invoice, pro forma invoice or purchase order can be requested.

Payment of the publication charge and the signed Open Access Agreement form must be received before the article can be published online.

PROOFS

You will receive the proofs by e-mail approximately 2 weeks after all relevant files have been sent o our Production Office. Please return them within 48 hours and if there should be any problems, please contact the production office at embopressproduction@wiley.com.

Please inform us if there is likely to be any difficulty in reaching you at the above address at that time. Failure to meet our

deadlines may result in a delay of publication.

All further communications concerning your paper proofs should quote reference number EMM-2022-15984-V3 and be directed to the production office at embopressproduction@wiley.com.

Thank you,

Zeljko Durdevic
Editor
EMBO Molecular Medicine

EMBO Press Author Checklist

Corresponding Author Name: Riccardo Brambilla	
Journal Submitted to: EMBO Molecular Medicine	
Manuscript Number: EMM-2022-15984-V2	

USEFUL LINKS FOR COMPLETING THIS FORM
The EMBO Journal - Author Guidelines
EMBO Reports - Author Guidelines
Molecular Systems Biology - Author Guidelines
EMBO Molecular Medicine - Author Guidelines

Reporting Checklist for Life Science Articles (updated January
This checklist is adapted from Materials Design Analysis Reporting (MDAR) Checklist for Authors. MDAR establishes a minimum set of requirements in transparent reporting in the life sciences (see Statement of Task: 10.31222/osf.io/9sm4x). Please follow the journal's guidelines in preparing your
Please note that a copy of this checklist will be published alongside your article.

Abridged guidelines for figures
1. Data
The data shown in figures should satisfy the following conditions:

- the data were obtained and processed according to the field's best practice and are presented to reflect the results of the experiments in an accurate and unbiased manner.
- ideally, figure panels should include only measurements that are directly comparable to each other and obtained with the same assay.
- plots include clearly labeled error bars for independent experiments and sample sizes. Unless justified, error bars should not be shown for technical
- if n<5, the individual data points from each experiment should be plotted. Any statistical test employed should be justified.
- Source Data should be included to report the data underlying figures according to the guidelines set out in the authorship guidelines on Data

2. Captions
Each figure caption should contain the following information, for each panel where they are relevant:

- a specification of the experimental system investigated (eg cell line, species name).
- the assay(s) and method(s) used to carry out the reported observations and measurements.
- an explicit mention of the biological and chemical entity(ies) that are being measured.
- an explicit mention of the biological and chemical entity(ies) that are altered/varied/perturbed in a controlled manner.
- the exact sample size (n) for each experimental group/condition, given as a number, not a range;
- a description of the sample collection allowing the reader to understand whether the samples represent technical or biological replicates (including how many animals, litters, cultures, etc.).
- a statement of how many times the experiment shown was independently replicated in the laboratory.
- definitions of statistical methods and measures:
  - common tests, such as t-test (please specify whether paired vs. unpaired), simple χ2 tests, Wilcoxon and Mann-Whitney tests, can be unambiguously identified by name only, but more complex techniques should be described in the methods section;
  - are tests one-sided or two-sided?
  - are there adjustments for multiple comparisons?
  - exact statistical test results, e.g., P values = x but not P values < x;
  - definition of 'center values' as median or average;
  - definition of error bars as s.d. or s.e.m.

Please complete ALL of the questions below.
Select "Not Applicable" only when the requested information is not relevant for your study.

Materials

Newly Created Materials	Information included in the manuscript?	In which section is the information available? (Reagents and Tools Table, Materials and Methods, Figures, Data Availability Section)
New materials and reagents need to be available; do any restrictions apply?	Yes	Material and Methods

Antibodies	Information included in the manuscript?	In which section is the information available? (Reagents and Tools Table, Materials and Methods, Figures, Data Availability Section)
For **antibodies** provide the following information:<br>- Commercial antibodies: RRID (if possible) or supplier name, catalogue number and or/clone number<br>- Non-commercial: RRID or citation	Yes	Material and Methods

DNA and RNA sequences	Information included in the manuscript?	In which section is the information available? (Reagents and Tools Table, Materials and Methods, Figures, Data Availability Section)
**Short novel DNA or RNA including primers, probes**: provide the sequences.	Yes	Material and Methods

Cell materials	Information included in the manuscript?	In which section is the information available? (Reagents and Tools Table, Materials and Methods, Figures, Data Availability Section)
**Cell lines:** Provide species information, strain. Provide accession number in repository **OR** supplier name, catalog number, clone number, and/**OR** RRID.	Yes	Material and Methods
**Primary cultures:** Provide species, strain, sex of origin, genetic modification status.	Yes	Material and Methods
Report if the cell lines were recently **authenticated** (e.g., by STR profiling) and tested for mycoplasma contamination.	Not Applicable	

Experimental animals	Information included in the manuscript?	In which section is the information available? (Reagents and Tools Table, Materials and Methods, Figures, Data Availability Section)
**Laboratory animals or Model organisms:** Provide species, strain, sex, age, genetic modification status. Provide accession number in repository **OR** supplier name, catalog number, clone number, **OR** RRID.	Yes	Material and Methods
**Animal observed in or captured from the field:** Provide species, sex, and age where possible**.**	Not Applicable	
Please detail **housing and husbandry conditions**.	Yes	Material and Methods

Plants and microbes	Information included in the manuscript?	In which section is the information available? (Reagents and Tools Table, Materials and Methods, Figures, Data Availability Section)
**Plants:** provide species and strain, ecotype and cultivar where relevant, unique accession number if available, and source (including location for collected wild specimens).	Not Applicable	
**Microbes:** provide species and strain, unique accession number if available, and source.	Not Applicable	

Human research participants	Information included in the manuscript?	In which section is the information available? (Reagents and Tools Table, Materials and Methods, Figures, Data Availability Section)
If collected and within the bounds of privacy constraints report on age, sex and gender or ethnicity for all study participants.	Not Applicable	

Core facilities	Information included in the manuscript?	In which section is the information available? (Reagents and Tools Table, Materials and Methods, Figures, Data Availability Section)
If your work benefited from core facilities, was their service mentioned in the acknowledgments section?	Not Applicable	

Design

Study protocol	Information included in the manuscript?	In which section is the information available? (Reagents and Tools Table, Materials and Methods, Figures, Data Availability Section)
If study protocol has been **pre-registered, provide DOI in the manuscript**. For clinical trials, provide the trial registration number **OR** cite DOI.	Not Applicable	
Report the **clinical trial registration number** (at ClinicalTrials.gov or equivalent), where applicable.	Not Applicable	

Laboratory protocol	Information included in the manuscript?	In which section is the information available? (Reagents and Tools Table, Materials and Methods, Figures, Data Availability Section)
Provide DOI OR other citation details if **external detailed step-by-step protocols** are available.	Not Applicable	

Experimental study design and statistics	Information included in the manuscript?	In which section is the information available? (Reagents and Tools Table, Materials and Methods, Figures, Data Availability Section)
Include a statement about **sample size** estimate even if no statistical methods were used.	Yes	Material and Methods, Figures
Were any steps taken to minimize the effects of subjective bias when allocating animals/samples to treatment (e.g. **randomization procedure**)? If yes, have they been described?	Yes	Material and Methods
Include a statement about **blinding** even if no blinding was done.	Yes	Material and Methods
Describe **inclusion/exclusion criteria** if samples or animals were excluded from the analysis. Were the criteria pre-established?<br><br>If sample or data points were omitted from analysis, report if this was due to attrition or intentional exclusion and provide justification.	Not Applicable	
For every figure, are **statistical tests** justified as appropriate? Do the data meet the assumptions of the tests (e.g., normal distribution)? Describe any methods used to assess it. Is there an estimate of variation within each group of data? Is the variance similar between the groups that are being statistically compared?	Yes	Appendix

Sample definition and in-laboratory replication	Information included in the manuscript?	In which section is the information available? (Reagents and Tools Table, Materials and Methods, Figures, Data Availability Section)
In the figure legends: state number of times the experiment was **replicated** in laboratory.	Yes	Figure legends
In the figure legends: define whether data describe **technical or biological replicates**.	Yes	Figure legends

Ethics

Ethics	Information included in the manuscript?	In which section is the information available? (Reagents and Tools Table, Materials and Methods, Figures, Data Availability Section)
Studies involving **human participants**: State details of **authority granting ethics approval** (IRB or equivalent committee(s), provide reference number for approval.	Not Applicable	
Studies involving **human participants**: Include a statement confirming that **informed consent** was obtained from all subjects and that the experiments conformed to the principles set out in the WMA Declaration of Helsinki and the Department of Health and Human Services Belmont Report.	Not Applicable	
Studies involving **human participants: For publication of patient photos**, include a statement confirming that consent to publish was obtained.	Not Applicable	
Studies involving experimental **animals**: State details of **authority granting ethics approval** (IRB or equivalent committee(s), provide reference number for approval. Include a statement of compliance with ethical regulations.	Yes	Material and Methods
Studies involving **specimen and field samples:** State if relevant **permits** obtained, provide details of authority approving study; if none were required, explain why.	Not Applicable	

Dual Use Research of Concern (DURC)	Information included in the manuscript?	In which section is the information available? (Reagents and Tools Table, Materials and Methods, Figures, Data Availability Section)
Could your study fall under dual use research restrictions? Please check biosecurity documents and list of **select agents and toxins** (CDC): https://www.selectagents.gov/sat/list.htm	Not Applicable	
If you used a select agent, is the security level of the lab appropriate and reported in the manuscript?	Not Applicable	
If a study is subject to dual use research of concern regulations, is the name of the **authority granting approval and reference number** for the regulatory approval provided in the manuscript?	Not Applicable	

Reporting

The MDAR framework recommends adoption of discipline-specific guidelines, established and endorsed through community initiatives. Journals have their own policy about requiring specific guidelines and recommendations to complement MDAR.

Adherence to community standards	Information included in the manuscript?	In which section is the information available? (Reagents and Tools Table, Materials and Methods, Figures, Data Availability Section)
State if relevant guidelines or checklists (e.g., **ICMJE, MIBBI, ARRIVE, PRISMA**) have been followed or provided.	Not Applicable	
For **tumor marker prognostic studies,** we recommend that you follow the **REMARK** reporting guidelines (see link list at top right). See author guidelines, under 'Reporting Guidelines'. Please confirm you have followed these guidelines.	Not Applicable	
For **phase II and III randomized controlled trials**, please refer to the **CONSORT** flow diagram (see link list at top right) and submit the CONSORT checklist (see link list at top right) with your submission. See author guidelines, under 'Reporting Guidelines'. Please confirm you have submitted this list.	Not Applicable	

Data Availability

Data availability	Information included in the manuscript?	In which section is the information available? (Reagents and Tools Table, Materials and Methods, Figures, Data Availability Section)
Have **primary datasets** been deposited according to the journal's guidelines (see 'Data Deposition' section) and the respective accession numbers provided in the Data Availability Section?	Not Applicable	
Were **human clinical and genomic datasets** deposited in a public access-controlled repository in accordance to ethical obligations to the patients and to the applicable consent agreement?	Not Applicable	
Are **computational models** that are central and integral to a study available without restrictions in a machine-readable form? Were the relevant accession numbers or links provided?	Not Applicable	
If publicly available data were reused, provide the respective **data citations in the reference list**.	Not Applicable	
