# Supplementary material for: Nuclear ERK1/2 signaling potentiation enhances neuroprotection and cognition via Importinα1/KPNA2
Source: EMBO Mol Med. 2023 Oct 4;15(11):e15984. doi: 10.15252/emmm.202215984 (PMC10630888; doi:10.15252/emmm.202215984)
Supplement: Supplementary file 3 — Source Data for Expanded View [file EMMM-15-e15984-s011.zip › Figure EV1.zip!/Figure EV1/Fig_EV1_-_panel_A.pdf]

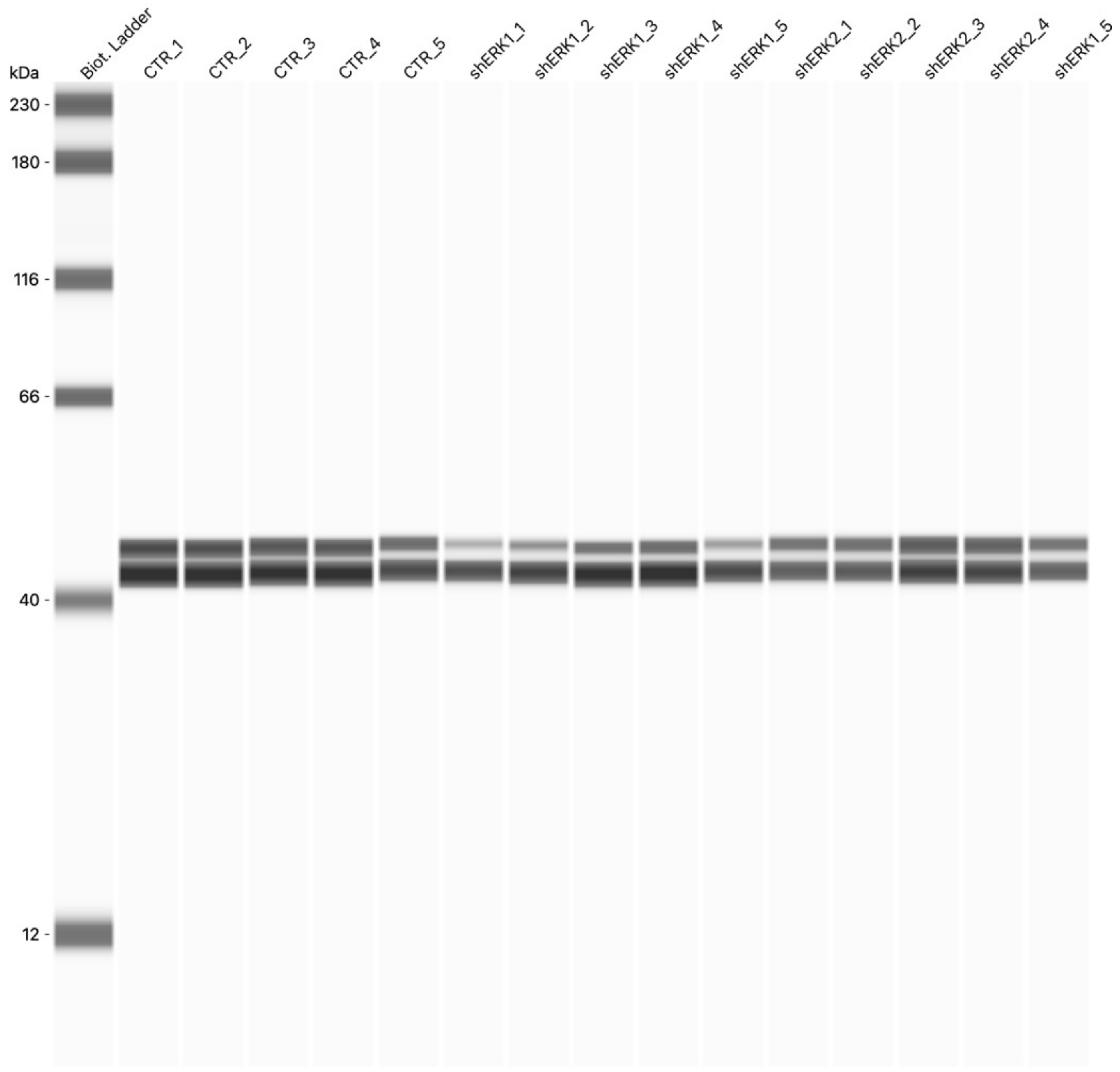
kDa
230
180
116
66
40
12
Biot. Ladder
CTR_1
CTR_2
CTR_3
CTR_4
CTR_5
shERK1_1
shERK1_2
shERK1_3
shERK1_4
shERK1_5
shERK2_1
shERK2_2
shERK2_3
shERK2_4
shERK1_5
