# Supplementary material for: Nuclear ERK1/2 signaling potentiation enhances neuroprotection and cognition via Importinα1/KPNA2
Source: EMBO Mol Med. 2023 Oct 4;15(11):e15984. doi: 10.15252/emmm.202215984 (PMC10630888; doi:10.15252/emmm.202215984)
Supplement: Supplementary file 8 — Source Data for Figure 4 [file EMMM-15-e15984-s002.zip › Figure 4/FIG_4_-_panel_f.zip!/FIG 4 - panel f/uncropped western blot_panel f.pdf]

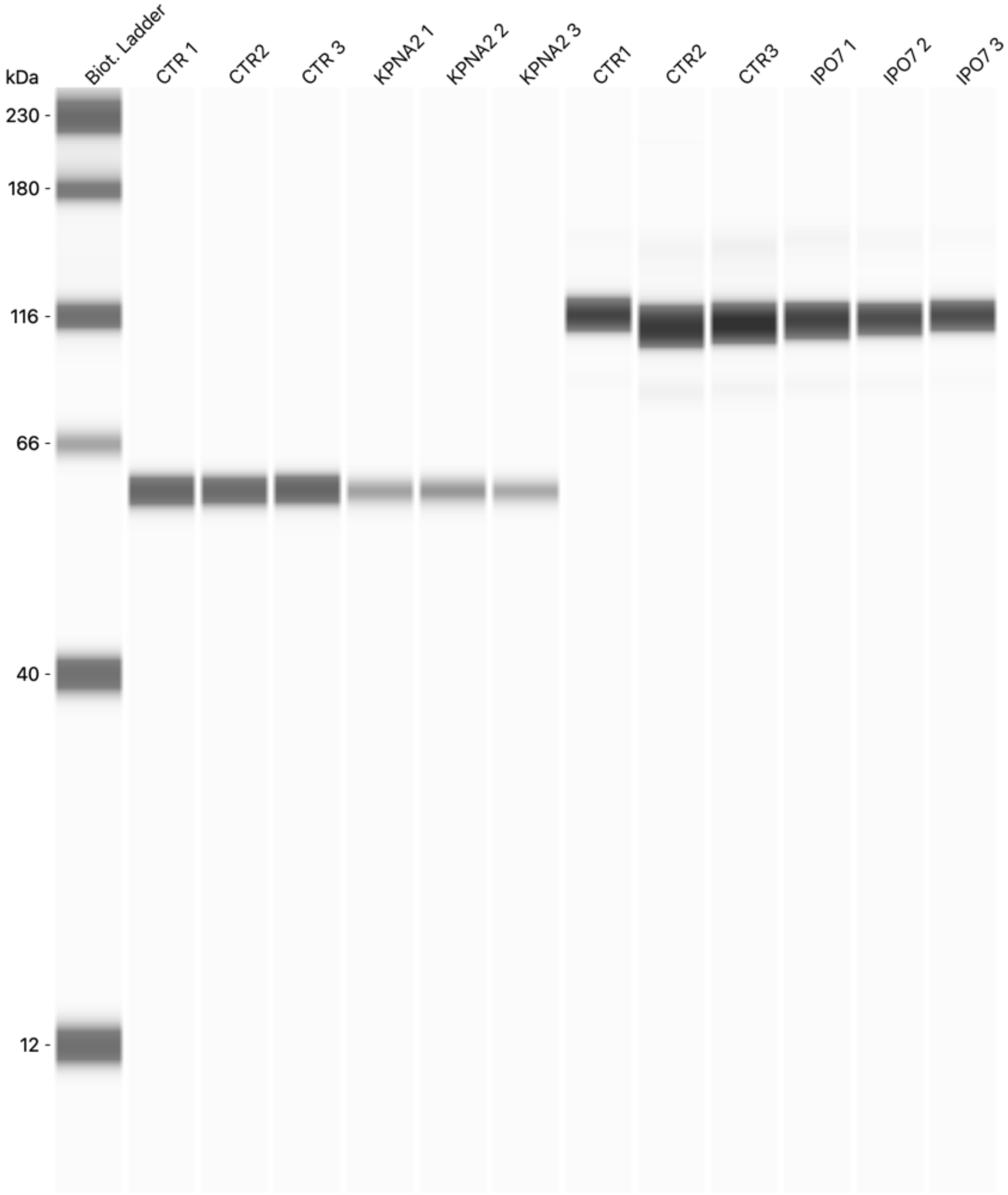
kDa
Biot. Ladder
CTR 1
CTR2
CTR 3
KPNA2 1
KPNA2 2
KPNA2 3
CTR1
CTR2
CTR3
IPO7 1
IPO7 2
IPO7 3
230
180
116
66
40
12
